# Multiscale low-dimensional motor cortical state dynamics predict naturalistic reach-and-grasp behavior

**DOI:** 10.1038/s41467-020-20197-x

**Published:** 2021-01-27

**Authors:** Hamidreza Abbaspourazad, Mahdi Choudhury, Yan T. Wong, Bijan Pesaran, Maryam M. Shanechi

**Affiliations:** 1 Ming Hsieh Department of Electrical and Computer Engineering, Viterbi School of Engineering, University of Southern California, Los Angeles, CA 90089 USA; 2 Center for Neural Science, New York University, New York City, NY 10003 USA; 3 Neuroscience Graduate Program, University of Southern California, Los Angeles, CA 90089 USA; 4 Department of Biomedical Engineering, University of Southern California, Los Angeles, CA 90089 USA; 5 Present Address: Department of Physiology, and Electrical and Computer Systems Engineering, Monash University, Melbourne, VIC 3800 Australia

**Keywords:** Dynamical systems, Neural encoding, Motor cortex

## Abstract

Motor function depends on neural dynamics spanning multiple spatiotemporal scales of population activity, from spiking of neurons to larger-scale local field potentials (LFP). How multiple scales of low-dimensional population dynamics are related in control of movements remains unknown. Multiscale neural dynamics are especially important to study in naturalistic reach-and-grasp movements, which are relatively under-explored. We learn novel multiscale dynamical models for spike-LFP network activity in monkeys performing naturalistic reach-and-grasps. We show low-dimensional dynamics of spiking and LFP activity exhibited several principal modes, each with a unique decay-frequency characteristic. One principal mode dominantly predicted movements. Despite distinct principal modes existing at the two scales, this predictive mode was multiscale and shared between scales, and was shared across sessions and monkeys, yet did not simply replicate behavioral modes. Further, this multiscale mode’s decay-frequency explained behavior. We propose that multiscale, low-dimensional motor cortical state dynamics reflect the neural control of naturalistic reach-and-grasp behaviors.

## Abstract (web summary)

Motor control involves neural dynamics at multiple spatiotemporal scales. Here the authors show that a multiscale, low-dimensional dynamical structure that is shared between scales and subjects reflects naturalistic reach-and-grasp movements in macaques.

## Introduction

Populations of motor cortical neurons control voluntary movement, but how they do so remains controversial. Early studies looked for direct relationships between neuronal firing rates and movement parameters to emphasize how individual neurons encode movement parameters such as velocity or direction^1,2^. The heterogeneous representation of movement parameters across a population of neurons and variations of movement modulation in individual neurons across conditions^3,4^ motivated later studies to explore how neural activity at the population level encodes movements^5–16^. To extract explanatory features, these studies used dimensionality reduction to learn how the activity of the neural population as a whole evolves in time during movements, i.e., to learn low-dimensional neural population dynamics^17^. These low-dimensional dynamics reflect coordinated population activity, which cannot be learned and interpreted from single-neuron activities^17^ and are predictive of movements^15,18–22^.

To date, studies of low-dimensional motor cortical population dynamics have largely examined a single scale of population activity^5–16^, mostly the spiking activity of an ensemble of neurons, which we refer to as spiking network activity. In addition, even for spiking network activity, prior models of low-dimensional population dynamics mainly investigate them within behavioral paradigms that instruct isolated trial-based reach movements^5–7,9,11,12,14,15^, or in a few cases isolated trial-based reach-to-grasp movements typically to fixed locations and within relatively limited spatial coordinates^10,13,20^. However, in our natural behaviors, reach and grasp movements evolve continuously in time rather than in an isolated manner and are coordinated. Indeed, prior studies have found that the firing rate of individual neurons in motor cortex is modulated by the kinematics of multiple joints in more naturalistic reach-and-grasp setups and have shown successful decoding of these joint angles from motor cortical spiking and field potential activity^23,24^. However, the low-dimensional neural population dynamics of continuous reach-and-grasp movements in naturalistic experimental setups are largely under-explored even for spiking activity. Further, neural control of arm and hand movements may reflect the integrated action of brain networks^25–27^; as a result, this neural control is distributed across multiple spatial and temporal scales of neural activity, spanning not only neuronal spiking activity but also larger-scale neural population activity reflected in local field potentials (LFP)^8,16,24,25,28–35^. How multiple scales of low-dimensional population dynamics measured with simultaneous spike-LFP activity reflect the neural control of naturalistic reach-and-grasp movements has remained elusive to date.

We refer to neural population activity measured with LFP on multiple recording channels as LFP network activity. Unlike spiking activity, which measures all-or-none action potential events that ultimately drive muscle contraction, LFP activity is continuously varying and measures sub-threshold synaptic and dendritic activity that shapes when cells generate action potentials^27,36,37^. Therefore, spiking and LFP activity measure different biological processes with different spatial and temporal activity scales, and with different statistical profiles^27,35^. Like single-scale spiking activity, single-scale LFP activity has been directly related to movement parameters such as position, velocity, or direction^28,29,38,39^, studied using dimensionality reduction^8,16^, and used to decode movement parameters such as position, velocity, movement goal, and joint angles^24,40,41^. However, how the low-dimensional dynamics in LFP activity may relate to those in spiking activity is largely unknown. Studies looking at spiking and LFP activity together^24,28–35,38^ have generally focused on quantifying the amount of task-related information at each measurement scale rather than on studying and comparing their low-dimensional dynamics. Using decoding analyses in various brain regions, some studies find similar and comparable amount of task-related information in spiking and LFP activity^29,32,34^, whereas other studies suggest that each scale may reflect non-redundant aspects of an ongoing behavior^28,30,31,33,38^ (see Discussion). These different conclusions about whether behavior is similarly or differentially encoded in spiking and LFP activity further make it unclear how spiking and LFP dynamics would relate to each other or to reach-and-grasp behavior. Specifically, it is not clear whether low-dimensional dynamics of spiking and LFP network activity have similar or different temporal characteristics, i.e., whether they are shared or distinct. Further, it is not known how low-dimensional dynamics across the different scales similarly or differentially predict reach-and-grasp behavior and encode movement parameters. In addition to requiring a naturalistic reach-and-grasp task, investigating these questions requires developing a new machine learning algorithm that can extract the low-dimensional latent dynamics from combined spike-field activity—i.e., extract them across multiple scales simultaneously^42^ (Methods). Answering these questions will help understand how naturalistic reach-and-grasp movements are controlled by the integrated action of large-scale brain networks as well as smaller-scale neural processes.

Here, we propose that multiscale, low-dimensional motor cortical state dynamics reflect the neural control of naturalistic reach-and-grasp behavior owing to the integrated action of large-scale brain networks. We simultaneously record spiking and LFP activity across the motor cortices in two non-human primates (*macaca mulatta*) performing a naturalistic coordinated three-dimensional (3D) reach-and-grasp movement task to an object that was continuously moved to random diverse locations; the task further lacked overt movement instructions and was performed continuously in time. We deploy a novel algorithm to learn multiscale dynamics in spiking and LFP network activity and partition these dynamics into different modes. Each mode captures a unique dynamical characteristic of network activity and is specified by a pair of time decay and frequency in the temporal neural response to excitations (Methods). We find that spiking and LFP network activity exhibited several distinct principal modes in their dynamics among which one mode was dominant in predicting behavior in each case. Notably, this predictive mode was shared across scales with a similar dynamical decay-frequency characteristic, was again learned as a unified mode for spike-LFP activity in combination, was shared across experimental sessions and monkeys, yet did not simply replicate the modes in behavior trajectories. This shared multiscale mode was found despite spiking and LFP activity being analyzed from non-overlapping electrodes and despite significant differences in their biological significance and mathematical modeling. The multiscale low-dimensional mode integrated multiscale motor cortical state dynamics over longer time intervals than non-predictive principal modes and offered an explanation of how these dynamics predict naturalistic behavior.

## Results

### Neural recording and task set-up

**Fig. 1:**
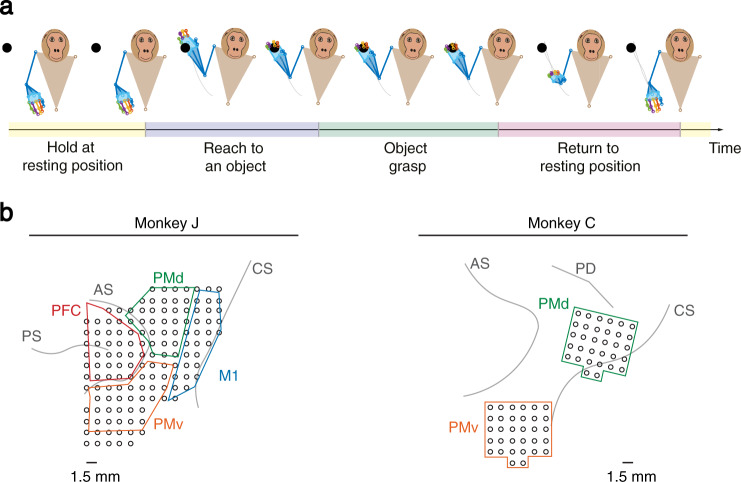
Task set-up and recording regions for two non-human primates (Monkey J and Monkey C). **a** Both subjects performed a 3D naturalistic reach-and-grasp task in a 50 cm × 50 cm × 50 cm workspace. An object located on a wand was always present in the 3D workspace and visible to the monkey. The wand was continuously moved by the experimenter to random locations spanning a large spatial area in front of the subjects. Subjects naturalistically reached to the object (without a go cue), grasped it, and then returned to the resting position. They performed the reach-and-grasp continuously in time for the whole course of the recording session (without a trial structure). There was no go cue, no instructions as to when to reach, and no requirements on reach, hold and grasp durations as the subjects performed the task. Subjects performed reaches to the objects, decided on the hold durations, reach durations, and grasp durations naturalistically. We used motion capture technology using retroreflective markers to track 27 (Monkey J) or 25 (Monkey C) joint angles on the right shoulder, elbow, wrist, and fingers at each point in time. The picture is recreated from marker trajectories of a sample recording session for Monkey J. **b** Recording regions covered primary motor cortex (M1), dorsal premotor cortex (PMd), ventral premotor cortex (PMv), and prefrontal cortex (PFC) for Monkey J and covered PMd and PMv for Monkey C. Recording electrodes are shown as black circles. Brain sulci (*PS* principal sulcus, *AS* accurate sulcus, *CS* central sulcus, *PD* precentral dimple) around the recording regions are shown by grey lines. The inter-electrode distance was 1.5 mm.

We simultaneously recorded spiking and LFP activity from motor cortical areas across multiple days from two Rhesus macaque monkeys (Monkey J and Monkey C) and analyzed recordings in the hemisphere contralateral to the arm and hand movement (Fig. 1). Recordings covered primary motor cortex (M1), dorsal premotor cortex (PMd), ventral premotor cortex (PMv), and prefrontal cortex (PFC) for Monkey J and PMd and PMv for Monkey C (see Fig. 1b and Methods).

Both monkeys performed a 3D reach, grasp, and return movement naturalistically in a 50 cm × 50 cm × 50 cm workspace and continuously in time (Fig. 1a). Monkeys naturalistically reached for an object positioned on a wand, grasped the object, then released the object and returned their hand to a resting position (Fig. 1a). An experimenter continuously moved the wand to random locations spanning a large spatial area in front of the monkey (Supplementary Table 1). Also, critically, the task allowed each monkey to choose how to reach-and-grasp the object, lacked instructions needed to isolate reach-and-grasp movement components and, in fact, lacked overt movement instructions in general. There was no go cue, no time-limit was enforced instructing how fast to reach, grasp, or return, and no targets appeared suddenly as the wand was always visible and generally moving. We used motion capture technology using retroreflective markers to track 27 (Monkey J) or 25 (Monkey C) joint angles on the right shoulder, elbow, wrist, and fingers at each point in time^43^ (Methods). Marker trajectories were tracked using infrared and near-infrared cameras^43^. Arm and finger joint angles were then obtained from the marker trajectories by solving for the inverse kinematics using an anatomically-correct non-human primate (NHP) musculoskeletal model^44^ (Methods). Consistent with the expression of a naturalistic behavior, each animal could develop their own behavioral strategy for performing the task.

### Multiscale dynamical modeling and modal analysis

**Fig. 2:**
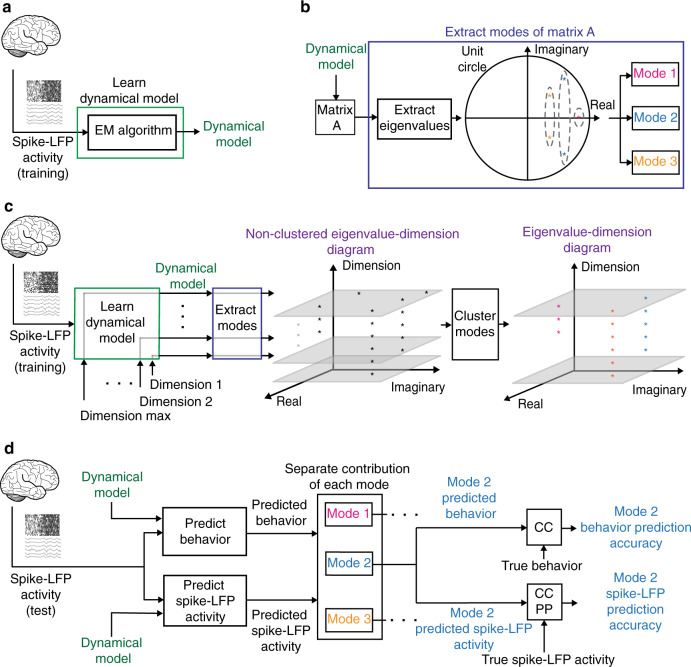
Multiscale dynamical modeling and modal analysis. **a** We first learn the dynamical model from the neural activity in the training set. The neural activity can be in the form of spiking, LFP, or combined spike-LFP activity. **b** After learning the dynamical model, we find the principal modes that characterize neural dynamics. Each principal mode has a unique pair of dynamical characteristics consisting of a decay and frequency and indicating how fast one component of neural response decays in time and with what frequency it rings over time in response to excitations. **c** To get the principal modes, for the same neural activity, we learn dynamical models of various latent state dimensions. For each dimension, we indicate the location of the modes corresponding to their real and imaginary values on a plane parallel to the *x*–*y* plane and intersecting the *z* axis at that dimension. We finally cluster the modes using K-means clustering to find the vertical clusters, each corresponding to a different location on the *x*–*y* plane and thus different decay-frequency characteristics. **d** In the test set, we use the learned dynamical model to estimate the modes and states. We then use the estimated state to predict behavior and predict neural activity one-step-ahead into the future, and we separate the contribution of each mode in these predictions. By comparing the contribution of each mode with true behavior and neural activity, we get each modes’ behavior prediction accuracy and one-step-ahead prediction accuracy of neural activity. This accuracy is quantified with correlation coefficient (CC) for behavior and LFP features and with prediction power (PP) for spike events (Methods).

We binned the spikes in 10 ms time-bins to obtain their 0–1 time-series, with a 1 or 0 representing the presence or absence of a spike in a given time-bin, respectively. Given their relevance to encoding of behavior^32,34,35,45^, we computed the LFP log-power features in seven frequency bands every 50 ms using moving 300ms causal bins (Methods). These bands consisted of theta (4–8 Hz), alpha (8–12 Hz), beta 1 (12–24 Hz), beta 2 (24–34 Hz), gamma 1 (34–55 Hz), gamma 2 (65–95 Hz), and gamma 3 (130–170 Hz). In all our analyses, we chose the spike channels to be different from the LFP channels so that the results were not confounded by leakage effects^27^ (Methods and Discussion). We then learned a novel multiscale dynamical model with latent states for combined spike-LFP network activity using a multiscale expectation-maximization (EM) algorithm (Fig. 2a and Methods). This algorithm modeled the spikes as point processes with a 10 ms time-scale and LFP activity as linear Gaussian processes with a 50 ms time-scale^42^ and learned low-dimensional latent states to describe the combined spike-LFP dynamics. We also learned dynamical models for the spiking network activity alone and the LFP network activity alone using EM algorithms for point process^46^ and linear Gaussian models^47^, respectively (Fig. 2a; Methods). Model performance was assessed using cross-validation.

The dynamical models had a latent state-space formulation (Methods). The model summarized the dynamics of neural population activity in terms of a low-dimensional latent state whose time-evolution was modeled with a linear state equation. We emphasize that the state was latent and the state-space model characterized just the neural population activity and thus was learned unsupervised with respect to (i.e., blind to) behavior measurements. As such, the latent state described the low-dimensional dynamics of neural population activity (see Methods and Discussion). We refer to each real eigenvalue or each pair of complex-conjugate eigenvalues of the state transition matrix in the state equation as a mode (Fig. 2b). Each mode corresponds to a unique decay-frequency pair present within neural dynamics; each pair corresponds to one component of the neural response to excitations and indicates how fast this response decays in time and with what frequency it rings over time^48^. Thus, the modes of the state transition matrix summarize the dynamical characteristics of neural population activity (Methods).

**Fig. 3:**
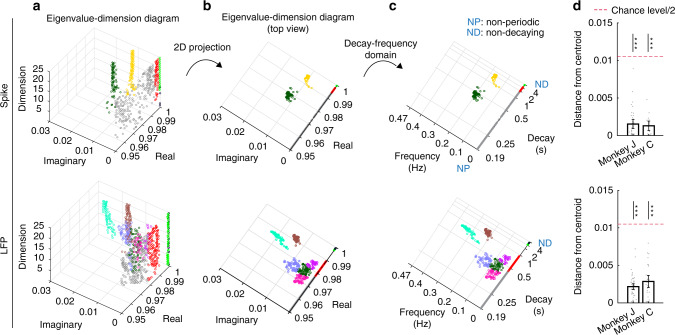
Both spiking and LFP network activity exhibit several principal modes in their dynamics. **a** Eigenvalue-dimension diagram for spiking (top) and LFP (bottom) network activity in one sample session. The vertical clusters are principal modes each shown with a different color. Modes that cannot be categorized in clusters are shown in grey. **b** The top view of eigenvalue-dimension diagram in **a**. For simplicity, in this view we show the modes for dimensions higher than 10. **c** The top view of eigenvalue-dimension diagram in (**b**) in the interpretable decay-frequency domain (Methods). NP and ND represent non-periodic or non-decaying dynamics, respectively. **d** Principal modes that form vertical clusters exist in every session. Each data point (grey dot) represents the mean distance of the members in one vertical mode cluster in one session from its centroid in the *x–y* plane. Data points are shown for all clusters in all sessions. Bars represent the mean over data points and error bars represent the 95% confidence bounds of this mean. A principal mode cluster must have a mean member distance to cluster centroid that is significantly smaller than half of chance-level distance (*P* < 1.6 × 10^−4^, *N*_*s*_ = 39, 17 (top) and *N*_*s*_ = 53, 31 (bottom) mode clusters across sessions in Monkey J and C, one-sided Wilcoxon signed-rank test, FDR-corrected) (Methods). Asterisks indicate significance of this comparison (*P < 0.05, **P < 0.005, and ***P < 0.0005). Source data are provided as a Source Data file.

To validate the learned modes, we used the fact that a principal mode of network dynamics should have consistent decay-frequency characteristics regardless of latent state dimension. We therefore compared the learned modes when increasing the dimension of the latent state from 1 to 25. We found the validated principal modes of network dynamics as those modes that were consistently learned at all dimensions greater than some value (Fig. 2c). We plot the modes within a 3D eigenvalue-dimension diagram whose *x*-*y* plane represents the real and imaginary components of the modes and whose *z* axis represents the state dimension (Methods and Fig. 2c). For modes with complex-conjugate eigenvalue pairs, we only show the eigenvalue with the positive imaginary component for simplicity of illustration. The distance between any two modes is defined as the norm of the difference in their corresponding eigenvalues, which is the Euclidean distance in the x-y plane of eigenvalue-dimension diagram. As a principal mode should have consistent decay-frequency characteristics regardless of dimension, the mode should appear at a similar location on the *x*-*y* plane for any dimension, thus forming a vertical cluster. We thus use K-means clustering to group the vertical clusters that persist through dimensions based on their location on the x-y plane (Fig. 2c; Methods). A vertical cluster is defined as one for which the distance of the members from the cluster centroid is significantly smaller than half of chance-level distance (Methods). With this criteria, members of a cluster are within a circle whose diameter is smaller than chance-level. We compute the chance-level as the mean distance of two eigenvalues placed randomly and uniformly on the eigenvalue planes in the range shown in Fig. 3 (chosen based on the observed modes in our data). This definition is a conservative chance-level because positive-imaginary eigenvalues can be anywhere on the upper unit semicircle (see Fig. 2b).

For each principal mode, in addition to the decay-frequency pair, we quantify how well the mode predicts behavior and neural activity (Supplementary Note 1 and Methods). Similar to prior work, we model the behavior, defined as joint angle trajectories or 3D end-point hand kinematics (positions and velocities in 3D physical space), as linear projections of the latent state^19,22,42,49^. After learning the dynamical model and estimating the latent states based on neural data alone in the training set, we fit the projection matrix in the training set to relate the already-estimated modes and states to behavior (Methods). In the test set, we first estimate the latent state using the learned dynamical model with a multiscale filter (Methods). We then use the fitted projection matrix to project the estimated latent states and predict the joint angles (Methods and Fig. 2d). Prediction accuracy is measured by the Pearson’s correlation coefficient (CC) between the actual and decoded trajectories in the test set. Unless otherwise stated, the term prediction accuracy refers to behavior prediction accuracy from neural activity. Finally, we find the prediction accuracy of each mode separately using a linear transformation of the latent states (Methods and Fig. 2d). We also assess the contribution of each mode to predicting the neural activity (Methods and Fig. 2d).

### Spiking and LFP network activity exhibited several principal modes

We first asked whether there existed clusters of principal modes in the spiking and LFP network activity, and if so what dynamical characteristics they had (Fig. 3). Figure 3a–c shows the eigenvalue-dimension diagram and its top view in both the eigenvalue plane and decay-frequency domain (for interpretability) for spiking and LFP activity in one experimental session. We found that clear vertical clusters of principal modes were formed across dimensions for both spiking and LFP activity, suggesting the existence of consistent dynamical characteristics that persist regardless of state dimension. Vertical clusters of principal modes were present in both monkeys and all experimental sessions (Supplementary Fig. 1). In every session, member distance from centroid in each principal mode cluster was small. In particular, this distance was 0.002 ± 0.002 (0.002 ± 0.001; mean ± sd) for the spiking network activity and 0.001 ± 0.001 (0.003 ± 0.002) for the LFP network activity in Monkey J (Monkey C) (Fig. 3d; chance-level distance = 0.021, *P* < 1.6 × 10^−4^, one-sided Wilcoxon signed-rank test, multiple comparisons across sessions were corrected by Benjamini–Hochberg false discovery rate (FDR)). As a control analysis, we obtained the eigenvalue-dimension diagram of randomly shuffled neural activity with the exact same procedure and found that principal complex-conjugate modes were not formed in shuffled activity (Supplementary Fig. 2).

**Fig. 4:**
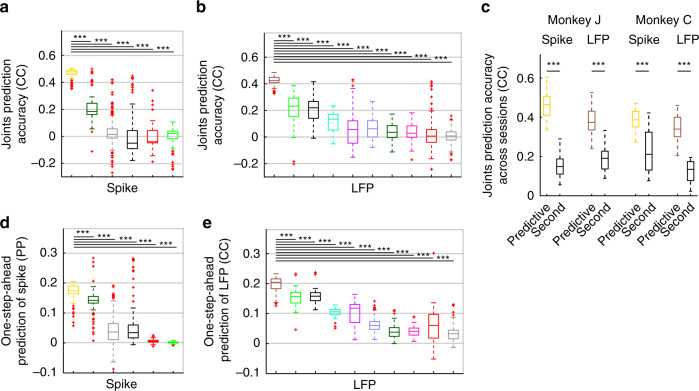
In both spiking and LFP activity, one mode is dominantly predictive of behavior. **a** Joint angle prediction accuracy statistics are shown for the members of the mode clusters in spiking network activity in the sample session shown in Fig. 3. Colors represent the colored clusters in Fig. 3. The yellow cluster termed predictive mode has significantly higher prediction accuracy than every other cluster (*P* < 8.1 × 10^−28^, *N*_*s*_ > 97 yellow cluster members, one-sided Wilcoxon rank sum test). The line inside boxes shows median, box edges represent the 25th and 75th percentiles, whiskers show the minimum and maximum values excluding outliers and red crosses indicate the outliers. Outliers are the points that are >1.5 times the box size away from the beginning and end of the box. **b** Similar to (**a**) for LFP network activity, where the brown cluster (predictive mode) has significantly higher prediction accuracy than every other cluster (*P* < 1.4 × 10^−4^, *N*_*s*_ > 99 brown cluster members, one-sided Wilcoxon rank sum test). **c** In each monkey, among all principal modes in spiking activity, the prediction accuracy of one of them (termed predictive mode) is significantly better than that of the second best principal mode shown by black box plots (yellow vs. black). The same result holds for LFP activity, and its predictive mode and second best principal mode (brown vs. black). Significance is shown by asterisks (*P* < 1.8 × 10^−20^, *N*_*s*_ = 35 and 20 cross-validation folds for Monkeys J and C, one-sided Wilcoxon signed-rank test). **d** Boxplot of the prediction power (PP) of mode clusters in predicting spiking activity. Higher prediction power indicates better one-step-ahead prediction of spiking activity. The same predictive mode that dominantly predicted behavior had the best one-step-ahead prediction of spiking activity (yellow mode cluster, *P* < 9.9 ×  10^−17^, *N*_*s*_ > 97 cluster members, one-sided Wilcoxon rank sum test). **e** Similar to (**d**) but for the correlation coefficient (CC) of mode clusters in one-step-ahead prediction of LFP. The same predictive mode cluster that dominantly predicted behavior had the best one-step-ahead prediction of LFP activity (brown mode cluster, *P* < 3.5 × 10^−11^, *N*_*s*_ > 99 cluster members, one-sided Wilcoxon rank sum test). Source data are provided as a Source Data file.

We then quantified how predictive of behavior each principal mode cluster was by predicting the joint angles of the arm during movement. In each individual session and in both spiking and LFP network activity, there was a principal mode cluster with a dominant prediction accuracy, which was significantly higher than the prediction accuracy of every other cluster (Fig. 4a, b; Monkey J: *P* < 6.9 × 10^−13^, Monkey C: *P* < 5.0 × 10^−10^, one-sided Wilcoxon rank sum test, FDR-corrected). We term this dominant mode the predictive mode. For spiking network activity, the mean prediction accuracy of the predictive mode across sessions for Monkey J (Monkey C) was 3.2 ± 0.96 (2.2 ± 1.11) times larger than that of the next best mode for prediction (Fig. 4c; Monkey J: *P* < 1.3 × 10^−7^, *N*_*s*_ = 35, Monkey C: *P* < 1.8 × 10^−4^, *N*_*s*_ = 20, one-sided Wilcoxon signed-rank test). Similarly, for LFP network activity, the mean prediction accuracy of the predictive mode across sessions for Monkey J (Monkey C) was 2.2 ± 0.71 (3.3 ± 1.9) times larger than that of the next best mode for prediction (Fig. 4c; Monkey J: *P* < 1.4 × 10^−7^, *N*_*s*_ = 35, Monkey C: *P* < 4.8 × 10^−5^, *N*_*s*_ = 20, one-sided Wilcoxon signed-rank test). Sample trajectories of the two latent states associated with just the predictive mode are shown in Supplementary Fig. 3a along with reach and return periods.

In addition to showing that the predictive mode is dominantly more predictive than the other modes above, we also compared the predictive mode’s prediction accuracy to the chance-level CC computed by comparing the predicted joint angles in the test set to time-shifted segments of joint angles in the training set as proposed in a prior work^23^. Consistent with our mode comparison results, for both spiking and LFP network activity, the predictive mode’s prediction accuracy was significantly better than this chance-level prediction (Supplementary Fig. 4; *P* < 1.0 × 10^−37^; *N*_*s*_ > 55; one-sided Wilcoxon rank sum test). We also found that the predictive mode was the same regardless of which aspect of movement (arm joint angles, finger joint angles, or end-point hand kinematics) was being predicted. In a control analysis, the predictive mode that had the best prediction accuracy for the arm joint angles, also predicted finger joint angles and end-point hand kinematics significantly better than other modes (Supplementary Fig. 3b–e; Monkey J: *P* < 1.3 × 10^−7^, *N*_*s*_ = 35; Monkey C: *P* < 4.8 × 10^−5^, *N*_*s*_ = 20; one-sided Wilcoxon signed-rank test). Finally, in an analysis for both spiking and LFP activity, we found that despite being very low-dimensional (corresponding to just a 2D state) and in addition to condition-independent information, the predictive mode also captured some condition-dependent information about reach-and-grasp movements. In particular, in addition to significantly decoding condition-independent movement trajectories—the trajectories averaged over targets, i.e., conditions—, the predictive mode also significantly decoded the condition-dependent residual trajectories—the trajectories for different targets minus the averaged trajectory (Supplementary Fig. 5; *P* < 8.3 × 10^−10^, *N*_*s*_ = 55, one-sided Wilcoxon signed-rank test).

Finally, this predictive mode for behavior was also dominant in describing neural dynamics across sessions. We quantified the relative contribution of each principal mode to neural dynamics by asking how well it could predict the neural activity one-step-ahead into the future (Methods). For both the spiking and LFP network activity, the one-step-ahead prediction of neural activity was significantly better for the behavior predictive mode compared with every other mode (Fig. 4d, e and Supplementary Fig. 6; Monkey J: *P* < 1.1 × 10^−3^, *N*_*s*_ = 35, Monkey C: *P* < 2.1 × 10^−3^, *N*_*s*_ = 20, one-sided Wilcoxon signed-rank test).

### The predictive mode was multiscale

**Fig. 5:**
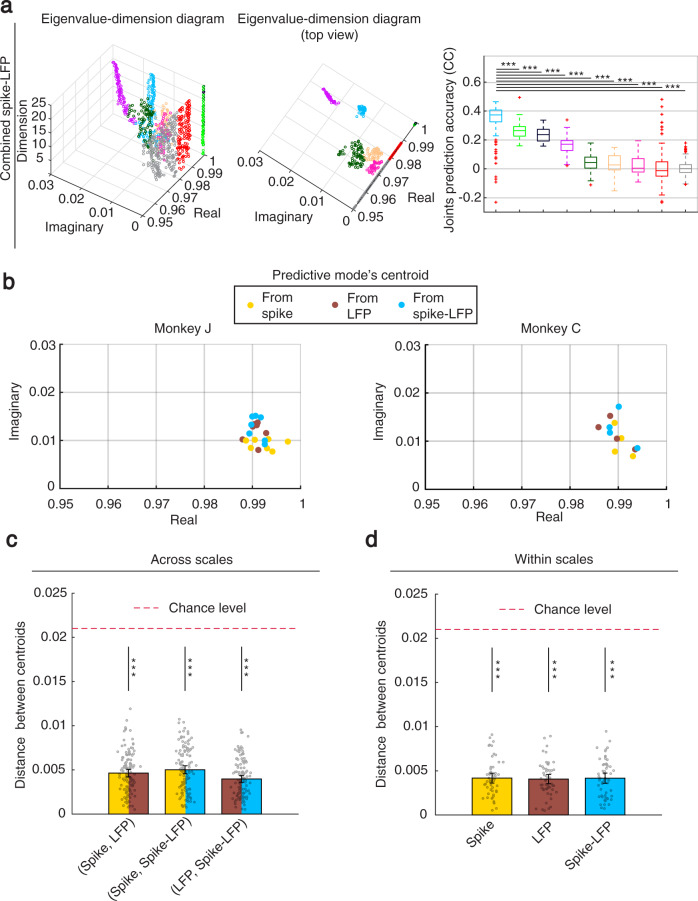
The predictive mode is multiscale, i.e., is shared across scales of brain activity. **a** Eigenvalue-dimension diagram for combined spike-LFP activity. Figure convention is the same as Figs. 3 and 4. Combined spike-LFP network activity also exhibited several principal modes among which again one (cyan) was dominantly predictive of behavior (*P* < 3.4 × 10^−12^, *N*_*s*_ > 125 cyan cluster members, one-sided Wilcoxon rank sum test). Boxplot outliers are a small fraction of data and mainly correspond to learned mode members at low dimensions when mode estimation has not converged yet. **b** The predictive modes across sessions in the two monkeys. Each dot represents the centroid of the predictive mode cluster in one experimental session for spiking (yellow), LFP (brown) and combined spike-LFP (cyan) network activity. **c** The location of the predictive mode was similar across scales of activity. The distance between the predictive mode centroids across scales, pooled across monkeys and experimental sessions was significantly smaller than chance-level (*P* < 6.8 × 10^−22^, *N*_*s*_ = 121 possible pairwise distances across scales, one-sided Wilcoxon signed-rank test). Each bar represents the mean of all possible pairwise distances between cluster centroids across scales shown in (**b**). For example, yellow-brown bar represents the mean of all pairwise distances between any yellow and brown dot in (**b**). Grey dots show all pairwise distances. Error bars show 95% confidence bound of the mean. Asterisks show the significance of comparison with chance-level distance with a similar convention as in Fig. 3. **d** The location of the predictive mode was similar across monkeys and experimental sessions (*P* < 5.7 × 10^−11^, *N*_*s*_ = 55 possible pairwise distances within scales, one-sided Wilcoxon signed-rank test). Same as (**c**) but for pairwise distances between cluster centroids within scales in (**b**). For example, yellow bar represents the mean of all pairwise distances between any two yellow dots in (**b**). Source data are provided as a Source Data file.

Although distinct modes were present in spiking and LFP network activity (Fig. 3 and Supplementary Fig. 1), consistent with different biological processes, the predictive mode was shared between spiking and LFP activity (Fig. 5). This was the case even though the analyzed spiking and LFP activity were recorded from non-overlapping channels, had different time-scales and statistical distributions in the dynamical models, and had their models learned with different algorithms (Methods and Discussion). Two sets of analyses supported this conclusion (Fig. 5).

First, we found that the distance between the predictive mode in the spiking network activity and that in the LFP network activity was small. We computed this distance at the maximum dimension in Fig. 3. In each session, this distance was 0.005 ± 0.001 for Monkey J and 0.004 ± 0.002 for Monkey C, which was significantly smaller than chance-level in both cases (Fig. 5b; chance-level distance = 0.021; Monkey J: *P* < 1.3 × 10^−7^, *N*_*s*_ = 35; Monkey C: *P* < 4.8 × 10^−5^, *N*_*s*_ = 20, one-sided Wilcoxon signed-rank test). This distance was significantly smaller than chance-level even when we randomly selected non-overlapping LFP channels (Monkey J distance: 0.006 ± 0.003; Monkey C distance: 0.006 ± 0.006; *P* < 1.4 × 10^−9^, *N*_*s*_ = 1050 and *N*_*s*_ = 600, for Monkeys J and C, respectively, for 30 random selections; one-sided corrected resampled paired *t* test, see Methods). Further, even when pooling the results of all sessions in each monkey, the distance between the predictive mode cluster centroids in spiking and LFP network activity was significantly smaller than chance-level (Fig. 5b, c; Monkey J: *P* = 5.7 × 10^−10^, *N*_*s*_ = 49; Monkey C: *P* = 2.4 × 10^−4^, *N*_*s*_ = 16, one-sided Wilcoxon signed-rank test); this pooled result shows that the mode was shared across scales even when comparing one session to another. Together, these results suggest that the dynamics of the predictive mode in spiking network activity—that is its decay-frequency—were very similar to the dynamics of the predictive mode in LFP network activity both within and across sessions.

Second, we learned a multiscale dynamical model for the combined spike-LFP network activity and found that a single principal mode cluster was again learned at a very similar location; further, this mode was again dominant in predicting behavior, i.e., was also the predictive mode for combined spike-LFP activity (Fig. 5a). In every experimental session and compared with every other cluster, this predictive mode cluster had significantly better prediction accuracy, which was on average 1.7 ± 0.35 and 4.4 ± 1.9 times larger than the accuracy of the second best mode cluster in Monkeys J and C, respectively (Fig. 5a; Monkey J: *P* < 1.4 × 10^−7^, *N*_*s*_ = 35; Monkey C: *P* < 4.8 × 10^−5^, *N*_*s*_ = 20, one-sided Wilcoxon signed-rank test). Example predicted movement trajectories with just the multiscale predictive mode are shown in Supplementary Fig. 7 for an extensive set of arm and finger joint angles and for end-point hand kinematics. In addition, across sessions, the distance between the predictive mode in the combined spike-LFP network activity and that in separate spiking or LFP network activity was on average 0.004 ± 0.001 (0.006 ± 0.002) for Monkey J (Monkey C), which were significantly smaller than chance-level (Fig. 5b; Monkey J: *P* < 1.3 × 10^−7^, *N*_*s*_ = 35; Monkey C: *P* < 4.8 × 10^−5^, *N*_*s*_ = 20, one-sided Wilcoxon signed-rank test). We refer to separate spiking activity and LFP activity as single-scale activity.

Finally, we found that the predictive mode stays at a similar location even when we remove the neural activity during the periods prior to movement initiation and relearn the models and modes. The distance of the relearned predictive mode to the original predictive modes in Fig. 5b was only 0.006 ± 0.003, 0.005 ± 0.002, and 0.004 ± 0.003 for spiking, LFP and combined spike-LFP activity, respectively, and was significantly smaller than chance-level (Supplementary Fig. 8; *P* < 3.9 × 10^−10^, *N*_*s*_ = 55, one-sided Wilcoxon signed-rank test). This result may suggest that the dynamical characteristics of the multiscale predictive mode were not significantly changed due to potential inputs to the motor cortex during planning/initiation phases of the movements (see Discussion). In line with this analysis, the prediction accuracy of the multiscale predictive mode was significantly higher than that of a smoothed onset-offset movement trajectory that regardless of the target, just smoothly turned the movement on when it started and off when the subject was at rest (Supplementary Fig. 9; *P* < 1.3 × 10^−6^; *N*_*s*_ = 55; one-sided Wilcoxon signed-rank test). Finally, we also shifted the multiscale predictive mode’s time-series in time and found that as the shift grows, the shifted mode loses its behavior prediction accuracy (Supplementary Fig. 10); as a shift keeps the autocorrelation of both behavior and neural activity intact, this result shows that the multiscale predictive modes’ prediction accuracies are not owing to the autocorrelations in behavior and/or neural activity.

Together, these results suggest that neural population dynamics that predict naturalistic reach-and-grasp behavior are multiscale, i.e., are shared across spatiotemporal scales reflected in both spiking and LFP activity. In what follows, we thus refer to this mode as the multiscale predictive mode.

### The multiscale predictive mode was not replicated in behavior dynamics

An important concern is that the multiscale predictive mode is shared across scales because it simply replicates or is otherwise a direct representation of a dominant mode in the dynamics of the behavior. To address this concern, we performed multiple analyses (Supplementary Notes 2 and 3).

First, we performed an extensive analysis of the behavior modes themselves. We found that the behavior modes exhibited a wider range of frequencies and larger decays compared to the multiscale predictive mode in neural activity (Supplementary Figs. 11 and 12). The mean decay and frequency of the multiscale predictive mode were 1.1 ± 0.49 s and 0.17 ± 0.03 Hz, respectively. In comparison, for the modes in joint angle trajectories, the decay was significantly larger than the multiscale predictive mode decay and ranged ~4–10 s (Supplementary Fig. 11; *P* = 3.5 × 10^−12^, *N*_*s*_ > 22, one-sided Wilcoxon rank sum test). Also, for these joint angle modes, the range of frequency was 0–0.49 Hz, which was significantly wider than the range of multiscale predictive mode frequencies (Supplementary Fig. 11; *P* = 7.1 × 10^−9^, *N*_*s*_ > 22, one-sided *F* test for equal variances). Similarly, for the end-point hand kinematics, these dynamical characteristics were 2–10 s and 0.17–1.5 Hz, respectively, thus leading to similar conclusions (Supplementary Fig. 12; *P* = 1.5 × 10^−9^, *N*_*s*_ > 22, one-sided Wilcoxon rank sum test; *P* = 1.6 × 10^−17^, *N*_*s*_ > 22, one-sided *F* test for equal variances).

Second, we computed the power spectral density (PSD) of the trajectories of behavior measurements and calculated the peak frequencies. These results further supported that behavior frequency ranges were wider than the multiscale predictive mode frequencies (Supplementary Figs. 11 and 12). The peak frequencies of the PSD for the joint angles and end-point hand kinematics were distributed between a wide range of 0–0.4 Hz and 0–1 Hz, respectively, consistent with the behavior modal analysis above.

Finally, using a simulation (Supplementary Note 3), we confirmed that if neural activity was simply representing behavior (and possibly some behavior-irrelevant processes), our analyses would have revealed the same modes in behavior and neural activity, unlike what we observe in our data (Supplementary Fig. 13 compared with Figs. 3 and 5, Supplementary Figs. 11 and 12). Taken together, these results indicate that the multiscale predictive mode in neural activity is not simply a direct representation of the behavior modes. To date, there is no consensus on whether and what movement parameters are directly encoded in neural activity^3,4,50^. Therefore, an exact match between the predictive neural modes and behavior modes, whether in joint angles or end-point hand kinematics, is not necessarily expected.

### The multiscale predictive mode was consistent across sessions and monkeys

Despite the naturalistic nature of our task, which allowed for variations in how each monkey chose to perform the task each day, we found that the multiscale predictive mode had a similar eigenvalue location both across sessions and across the two monkeys. The mean pairwise distance between the predictive mode cluster centroids pooled across all experimental sessions in both monkeys was only 0.004 ± 0.002 for spiking network activity and only 0.004 ± 0.002 for LFP network activity. These distances were both significantly smaller than chance-level, showing that the multiscale predictive mode and thus its decay-frequency characteristic was shared not only across scales but also across sessions and monkeys (Fig. 5b, d; chance-level distance = 0.021; *P* = 5.7 × 10^−11^, *N*_*s*_ = 55, one-sided Wilcoxon signed-rank test).

### Distance to the multiscale predictive mode explained behavior prediction accuracy

Given the existence of a multiscale predictive mode in spiking and LFP network activity, which was dominant in predicting behavior, we formed two hypotheses. We hypothesized that the distance of the closest estimated mode to the multiscale predictive mode should explain behavior prediction accuracy. We also hypothesized that this distance would be reduced when using combined spike-LFP activity to estimate the mode location. To test the above hypotheses, we created 280 (160) randomly selected channel sets containing non-overlapping spike and LFP channels for Monkey J (Monkey C). Each channel set used either five random spike channels or five random LFP channels as baseline, to which we then added 25 random LFP or spike channels, respectively (Methods). For each channel set, we repeated the cross-validated model fitting and behavior prediction process for both single-scale and combined spiking and LFP activity (Methods). For computational tractability, we fixed the dimension of the latent state to be 15 in these analyses, as we had found that it was high enough to capture the principal modes. We compared the principal modes learned in combined spike-LFP activity to those in either spiking or LFP activity in terms of their distance to the multiscale predictive mode (Methods). In each session, the location of the multiscale predictive mode that we compared with was defined as the average location of the predictive mode clusters in single-scale spiking and LFP network activity when using all channels to get a good estimate of its location (average of yellow and brown dots in that session in Fig. 5b). Then for each random channel set, we obtained the closest estimated mode to the multiscale predictive mode, both when estimating the mode from single-scale spiking activity or single-scale LFP activity separately and when estimating it from combined spike-LFP activity.

**Fig. 6:**
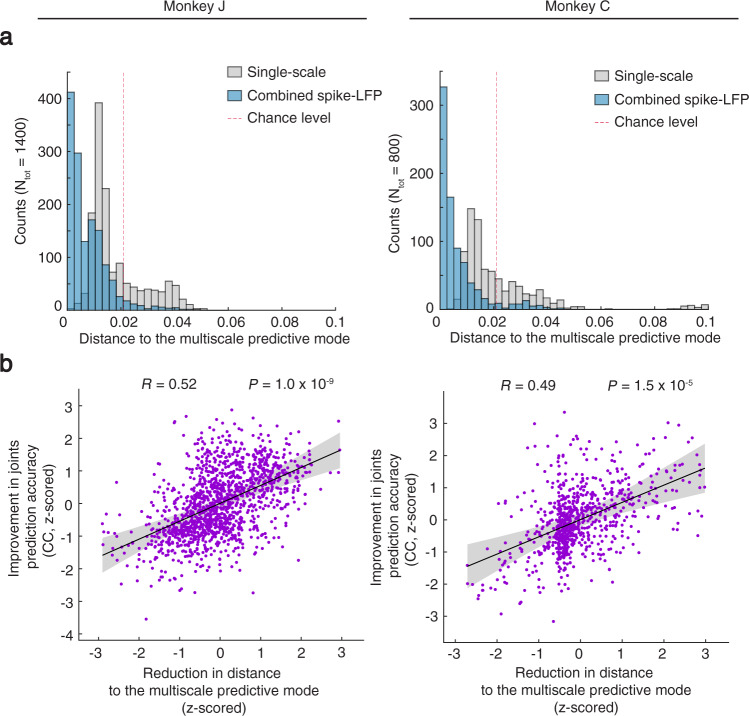
Distance to the multiscale predictive mode explains behavior prediction accuracy. **a** For both monkeys, using the combined spike-LFP activity improved the estimation of the multiscale predictive mode across channel sets (Methods). The distance of the estimated modes to the multiscale predictive mode significantly decreased when using spike-LFP activity in combination compared with separately. Each channel set used a baseline of random five LFP or spike single-scale channels and then added random 25 spike or LFP channels to them, respectively. The location of the multiscale predictive mode was defined as the average of the single-scale predictive modes estimated using all channels to get the best estimate (average of brown and yellow dots in Fig. 5b). **b** For both monkeys, there was a positive correlation between the reduction in distance and improvement in joint angle prediction accuracy in CC (*P* < 1.5 × 10^−5^, *N*_*s*_ = 1400 and 800 Monte Carlo samples in Monkey J and C, two-sided corrected resampled paired *t* test). Each dot represents one cross-validation fold from one channel set. To show the results across sessions, values are *z* scored for each session. R represents the Pearson’s correlation coefficient and P its *p* value corrected by the resampled *t* test (see Methods). Black line is the linear least-square fit representing the mean prediction accuracy for different distances and the shaded area is the 95% confidence bound of the mean. Source data are provided as a Source Data file.

We observed that using combined spike-LFP activity more accurately learned the multiscale predictive mode and resulted in better prediction accuracy. The distance to the multiscale predictive mode was significantly smaller when estimating the mode using combined spike-LFP activity compared with separate spiking or LFP activity (Fig. 6a; Monkey J: *P* = 2.6 × 10^−35^, *N*_*s*_ = 1400; Monkey C: *P* = 1.1 × 10^−10^, *N*_*s*_ = 800, one-sided corrected resampled paired *t* test, see Methods). Also, there was a significant positive correlation between the reduction in distance and the improvement in prediction accuracy when going from single-scale spiking or LFP activity to combined spike-LFP activity for both monkeys (Fig. 6b). These results also held when 10 single-scale spike or LFP channels were used as baseline in the randomly selected channel sets (Supplementary Fig. 14).

### The multiscale predictive mode was present in both low-frequency and high-frequency bands of LFP activity

The information encoded in spiking and high-frequency (gamma) band of LFP activity may be correlated as both may measure action potentials^31,51^. This raises the question of whether the multiscale predictive mode was simply owing to high-frequency band of LFP activity, and so closely related to spiking, or reflected activity at other frequency bands, which are not closely related to spiking.

**Fig. 7:**
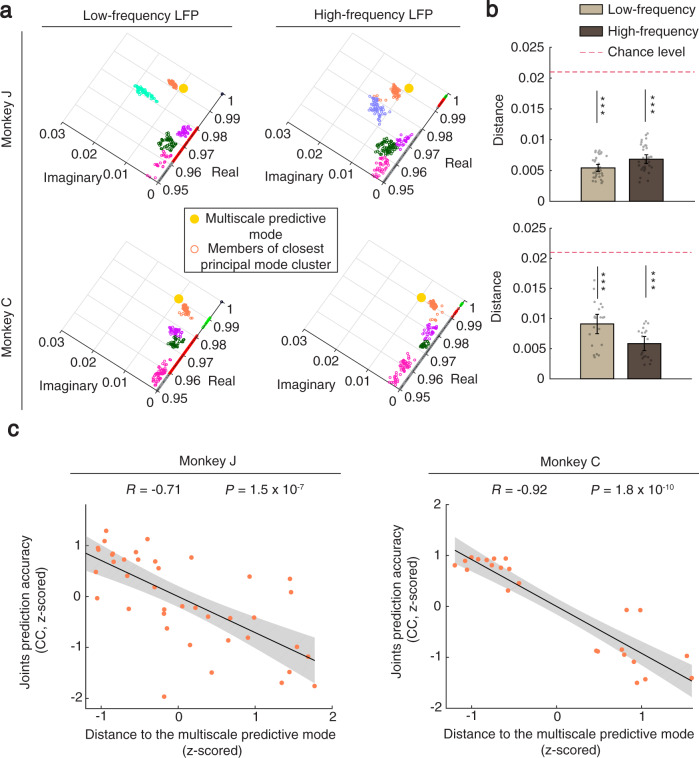
The multiscale predictive mode was present in both low-frequency and high-frequency bands of LFP activity. **a** For a sample experimental session (top: Monkey J, bottom: Monkey C), the top view of the eigenvalue-dimension diagram is shown for low-frequency (theta + alpha + beta; left) and high-frequency (gamma; right) bands of LFP activity. The yellow circle is the average location of the predictive mode clusters in the spiking network activity in Fig. 5b, which we used as the ground-truth location of the multiscale predictive mode in this analysis. The members of the closest estimated principal mode cluster to the yellow dot are shown in orange. **b** In both monkeys and for both low-frequency and high-frequency bands of LFP activity, the mean distance of the closest estimated principal mode cluster to the multiscale predictive mode was significantly smaller than chance-level as shown by the asterisks with similar convention as in Fig. 3 (*P* < 6.4 × 10^−5^, *N*_*s*_ = 35 and 20 cross-validation folds across sessions for Monkeys J and C, FDR-corrected, one-sided Wilcoxon signed-rank test). The distance in each session is calculated between the centroid of the orange cluster and the yellow dot in (**a**). Bars represent mean across sessions and error bars are 95% confidence bound of the mean. **c** The distance of the estimated principal mode to the multiscale predictive mode is correlated with the behavior prediction accuracy for different frequency band combinations of LFP activity (theta + alpha, beta, gamma in addition to their pairwise combinations: theta + alpha + beta, theta + alpha + gamma and beta + gamma; *P* < 1.5 × 10^−7^, *N*_*s*_ = 42 and 24 frequency band combinations across sessions for Monkeys J and C, one-sided Wilcoxon signed-rank test, FDR-corrected). For each monkey, each dot represents one frequency band combination for one session. The distance and the prediction accuracy of the estimated principal mode in each session are *z* scored and then results are pooled across sessions. Similar to Fig. 6, black line is the linear least-square fit representing the mean prediction accuracy and the shaded area is the 95% confidence bound of the mean. Source data are provided as a Source Data file.

To address this question, we performed the modal analysis on the low-frequency (theta + alpha + beta 1 + beta 2) and high-frequency (gamma 1 + gamma 2 + gamma 3) bands of LFP activity separately. We found that the multiscale predictive mode existed in both (Fig. 7). For both monkeys, the distance between the predictive mode in spiking activity in Fig. 5 and the closest estimated mode in either the low-frequency or the high-frequency bands of LFP activity was significantly smaller than chance-level (Fig. 7a, b; Monkey J: *P* = 1.3 × 10^−7^, *N*_*s*_ = 35; Monkey C: *P* = 6.4 × 10^−5^, *N*_*s*_ = 20, FDR-corrected, one-sided Wilcoxon signed-rank test). To better rule out the possibility that the multiscale predictive mode was present in low- and high-frequency bands of LFP activity simply because their frequency ranges ended and started at the same 34 Hz (Methods), respectively, we performed a control analysis in which the high-frequency bands of LFP activity started from 45 Hz (i.e., 11 Hz gap). We observed that again the multiscale predictive mode was present in both the low-frequency band and this high-frequency band (Supplementary Fig. 15).

Finally, since the multiscale predictive mode was also shared across frequency bands of LFP activity, we further validated the hypothesis that the behavior prediction accuracy should be explained by the distance between the multiscale predictive mode and the closest estimated mode when using different combinations of these frequency bands. We estimated the principal modes from various combinations of frequency bands of LFP activity (theta + alpha, beta and gamma in addition to their pairwise combinations). We found that the distance of the closest estimated principal mode to the multiscale predictive mode was significantly correlated with behavior prediction accuracy (Methods and Fig. 7c).

### Both the decay and frequency of the multiscale predictive mode explained naturalistic behavior

**Fig. 8:**
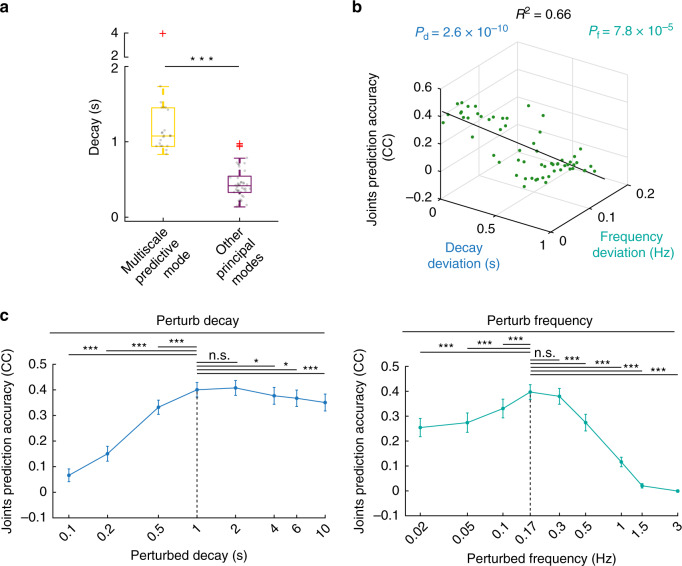
The multiscale predictive mode had a larger decay than other modes and both its decay and frequency explained naturalistic behavior prediction. **a** Multiscale predictive modes in Fig. 5 had significantly larger decays than other complex-conjugate modes in spiking and LFP activity (*P* = 9.1 × 10^−11^, *N*_*s*_ > 22 spiking and LFP sessions, one-sided Wilcoxon rank sum test). Figure convention for boxplot and significance (asterisks) is similar to Figs. 3 and 4. Each grey dot shows the decay of one principal mode across subjects and experimental sessions. **b** Multivariate linear regression (MVLR) relating the principal mode’s prediction accuracy to its decay and frequency deviation. The deviations were computed as the mode’s decay and frequency absolute difference from those of the multiscale predictive mode (Methods). Both decay and frequency had significantly negative coefficient with *p* values reported by *P*_d_ and *P*_f_, respectively (*N*_s_ = 66 complex-conjugate principal modes, two-sided paired *t* test). *R*^2^ shows the R-squared of the fitted MVLR model. Each green dot represents one complex-conjugate principal mode across subjects and experimental sessions. The black line shows the MVLR fitted line. **c** Perturbing decay (left) and frequency (right) of the multiscale predictive modes in the learned state-space model shows that both decay and frequency components explained naturalistic behavior prediction accuracy. Each dot represents the mean prediction accuracy of one perturbed mode across experimental sessions and monkeys; error bars represent 95% confidence bounds of the mean. The *x* axis is shown in log scale and represents the value of perturbed decay and frequency. Asterisks show whether the prediction of the perturbed mode is significantly different from that of the unperturbed multiscale predictive mode represented by a vertical dashed line, with conventions similar to Fig. 3 (n.s. is non-significant *P* > 0.05). Decays ~1−2 s had the best prediction accuracy (picked as the interval that had non-significant difference with the unperturbed mode, *P* = 4.1 × 10^−2^, *N*_*s*_ = 55 cross-validation folds, one-sided Wilcoxon signed-rank test, FDR-corrected). Also, mode frequencies ~0.17−0.3 Hz had the best prediction accuracy (picked as the interval that had non-significant difference compared with the unperturbed mode, *P* = 8.2 × 10^−5^, *N*_*s*_ = 55 cross-validation folds, one-sided Wilcoxon signed-rank test, FDR-corrected). Source data are provided as a Source Data file.

Multiple convergent analyses revealed that the temporal characteristics of the multiscale predictive mode, i.e., its decay and frequency, explain its behavior prediction accuracy. First, the multiscale predictive modes had significantly larger decays compared with every other complex-conjugate principal mode (Fig. 8a; *P* = 9.1 × 10^−11^, *N*_*s*_ > 22, one-sided Wilcoxon rank sum test). This indicates that accurate predictions of naturalistic behavior required integrating information over larger time intervals on the order of 1 s (Discussion).

Second, we fitted a multivariate linear regression (MVLR) model that wrote the behavior prediction accuracy of principal modes as a linear function of their decay and frequency deviation from the multiscale predictive mode (Supplementary Fig. 1 and Methods). We found that both decay and frequency deviation had significantly negative coefficients in the MVLR (Fig. 8b; frequency: *P* = 7.8 × 10^−5^; decay: *P* = 2.6 × 10^−10^, *N*_*s*_ = 66). This indicates that the further the decay or frequency of a principal mode was from the multiscale predictive mode, the lower its behavior prediction accuracy was.

Third, we perturbed the decay and frequency of the multiscale predictive mode and recomputed its behavior prediction accuracy (Methods). To do this, we first fixed the frequency of the multiscale predictive mode and perturbed its decay from 0.1–10 s while fixing all the other principal modes at their learned values. We then relearned the state-space model (all parameters except the state transition matrix that is dictated by the modes) for all experimental sessions from neural data (Methods). We repeated this analysis by similarly fixing the decay and perturbing the frequency from 0.02 Hz to 3 Hz for the multiscale predictive mode. For simplicity, we fixed the dimension of the latent state at 15. We found that the behavior prediction accuracy of the perturbed mode decreased significantly when going outside the vicinity of the multiscale predictive mode. This significant decrease occurred when the perturbed frequency was outside a range of ~0.17–0.3 Hz (Fig. 8c; *P* = 8.2 × 10^−5^, *N*_*s*_ = 55, one-sided Wilcoxon signed-rank test, FDR-corrected) or when the perturbed decay was outside a range of ~1–2 s (Fig. 8c; *P* = 4.1 × 10^−2^, *N*_*s*_ = 55, one-sided Wilcoxon signed-rank test, FDR-corrected). Further, a perturbation that decreased the decay value (faster decays) was more detrimental than one that increased the decay value (slower decays). Decreasing the decay value 10 times led to a decrease of 0.33 in CC of prediction accuracy (from 0.4 to 0.07) compared with a decrease of 0.05 (from 0.4 to 0.35) when increasing the decay 10 times (Fig. 8c). Therefore, prediction accuracy decreased more when the perturbation led to smaller decays (fast-decaying) compared to larger decays (slow-decaying), again indicating that integrating information over larger time intervals of at least 1 s is important in behavior prediction (Fig. 8c). Taken together, these convergent results suggest that both the decay and frequency temporal characteristics of the multiscale predictive mode are important in explaining how motor cortical state dynamics control naturalistic behavior.

## Discussion

We learned novel multiscale dynamical models in order to characterize the low-dimensional state dynamics at multiple scales of motor cortical population activity during naturalistic 3D reach-and-grasp movements. We observed that both motor cortical spiking and LFP network activity exhibited several principal modes of network state dynamics, with one predictive mode that predicted joint angles and end-point hand kinematics markedly better than the other principal modes. Importantly, despite the existence of several distinct modes in spiking and LFP network activity, the predictive mode was shared between them and reflected state dynamics that were multiscale and present in both spiking and LFP activity, in all experimental sessions and in both monkeys, yet did not simply replicate behavior modes.

The presence of a multiscale predictive mode suggests that a low-dimensional dynamical state that is multiscale controls the naturalistic movements of the arm and hand. Analyzing the distance between the multiscale predictive mode and the closest mode estimated from neural activity reinforced this conclusion. Estimating the state dynamics by adding spiking to LFP activity or vice versa reduced the distance of the estimated modes to the multiscale predictive mode and improved prediction accuracy. Moreover, both the decay and frequency components of the multiscale predictive mode were important in behavior prediction. Our work extends the dynamical system models of low-dimensional population dynamics in two directions. First, we model the low-dimensional population dynamics at multiple spatiotemporal scales. Second, we study these low-dimensional multiscale population dynamics in naturalistic 3D reach-and-grasp movements and identify their dynamical modes and decay-frequency characteristics. In doing so, we discover that the role of motor cortex in generating naturalistic reach-and-grasp movements occupies a low-dimensional multiscale dynamical state. We propose the multiscale predictive mode reflects, at least in part, the prominent role played by large-scale brain networks in the control of arm and hand movements.

Here, we modeled population dynamics at multiple scales simultaneously. How low-dimensional dynamics of large-scale brain networks control unconstrained, naturalistic movements remains elusive. To date, low-dimensional motor cortical population dynamics have been largely learned during the performance of constrained, instructed movement tasks and, further, by focusing on only a single scale of brain activity—mainly spiking activity. However, in the motor cortex^24,29,32–35^ and in related regions of the association cortices^28,30,31,38^, task-related information is present across different spatial and temporal scales of brain activity reflected in spiking and LFP measurements. Thus, it is important to study low-dimensional population dynamics at these multiple scales. Indeed, spiking and LFP activity reflect different biological processes^27,36,37^ and can differentially encode distinct aspects of an ongoing sensorimotor behavior^28,30,31,33,38^. For example, in the motor cortex, position is encoded better in spiking activity while LFP activity better encodes speed^33^. Also, both similar and differential representations in spiking and LFP activity have been observed in the auditory and visual cortices during naturalistic stimuli presentation^31^ and during ocular response to visual stimuli^30^. Similarly, in the posterior parietal cortex, decoding the direction of saccades and reaches from spiking and LFP activity yielded similar accuracy, but decoding the time of saccade or reach execution was more accurate using LFP compared with spiking^28,38^. Even though these works do not study low-dimensional population dynamics, they provide different conclusions regarding the nature and amount of task-related information at different scales. Therefore, if and how dynamical modes captured by spiking and LFP network activity relate to each other or to behavior has been unclear. Here, we addressed the above question and the role of low-dimensional dynamics in naturalistic motor control by learning state dynamics from both spiking and LFP network activity during naturalistic reach-and-grasp movements.

We study low-dimensional multiscale population dynamics during naturalistic reach-and-grasp tasks. Prior studies that focused on investigating individual neuron firings or on decoding have successfully decoded joint angles from spiking and field potential activity in naturalistic reach-and-grasp tasks^23,24^. However, naturalistic reach-and-grasp tasks are largely under-explored in studies that instead focus on the low-dimensional dynamics of neural populations as a whole; also, these studies largely explore low-dimensional dynamics at a single scale of population activity^5–16^. Instead, here we compare these low-dimensional population dynamics at multiple scales (i.e., in spiking and LFP network activity) rather than a single scale, and identify their modes and decay-frequency characteristics. Further, we do so during naturalistic 3D reach-and-grasp movements. Prior studies of low-dimensional population dynamics instead investigated single-scale activity during isolated trial-based reach movements^5–9,11,12,14,15^ or, in a few cases, isolated trial-based reach-to-grasp movements but to one or few fixed locations within a relatively limited spatial range^10,13,20^. In comparison, we studied a more naturalistic task in which reach-and-grasp movements happened to random locations that spanned a large spatial area (also the locations were not fixed spatially). Further, the object was always visible, and our task lacked instructions to isolate or constrain movement components and instead featured monkeys voluntarily coordinating reach and grasp movements continuously in time.

We discovered a multiscale predictive mode in our naturalistic 3D reach-and-grasp task that exhibited rotational characteristics, i.e., consisted of complex conjugate as opposed to real eigenvalues (Supplementary Fig. 16). This is consistent with the observation in prior tasks about the existence of rotational dynamics in single-scale network activity^7,8,10,15,16,19^. However, we observed that the principal modes in neural activity had a frequency range from 0–0.3 Hz, which is lower than the frequency of rotations reported in some studies for spike firing rates of ~0–2.5 Hz^7^ and for LFP activity of ~2–3 Hz^8,16^. There could be multiple reasons for this. First, as stated above, our task consisted of naturalistic 3D reach-and-grasp movements, which differ from instructed 2D hand-controlled cursor movements^7,8,19,22^ and button presses to track cursor movements^16^ in these prior studies. Second, these prior studies used a trial-based experimental design and analysis. In turn, they found the frequencies of projected rotations from analysis of trial-locked neural activity lasting for the relatively short analysis durations of ~200 ms^7^ or 400 ms^8,16^. In contrast, we use a continuous-time, naturalistic reach-and-grasp task and in turn model the whole course of neural activity for the full duration of the session—rather than short segments of it—that spans all the forward and backward movements as well as the holds. Consistent with our frequency range, recent work that considered the single-scale spiking activity during both the center-out forward and backward cursor movements together found that the frequencies within single-scale spiking activity were mostly ~0–0.5 Hz, which is closer to that in our results^19,22^; this study also found that these lower-frequency dynamics better decoded the cursor movement compared to higher-frequency ones (i.e., compared to 1–3 Hz)^22^. Finally, consistent with the frequency range of the principal modes, we found that single-channel activity also exhibited a low-frequency range in its PSD (Supplementary Fig. 17).

Interestingly, the multiscale predictive mode was also shared across sessions and monkeys. This means that the predictive neural population dynamics had the same decay-frequency in all sessions and both monkeys. This shared nature may be due to the general similarities in the performed naturalistic reach-and-grasp movements and importantly the preserved role of motor cortical regions in generating these movements. This finding is distinct from a recent study showing that single-scale neural population activity lives in preserved low-dimensional subspaces across different tasks^13^ by performing static dimensionality reduction. In particular, even if the low-dimensional subspace is the same, this does not imply that the principal modes within this subspace—which dictate how neural population activity evolves over time within this subspace—would also be the same. Here, we found that for a naturalistic reach-and-grasp task, the temporal decay-frequency characteristics of neural dynamics are the same across animals and sessions. It would be interesting in future work to examine whether and how multiscale modes may change as subjects switch between alternating tasks^52^ in one experimental session by developing adaptive or switching state-space models^18,53^ for multiscale activity.

Our goal is to investigate the low-dimensional latent dynamics and modes of neural populations and their decay-frequency across scales in naturalistic reach-and-grasp movements. Prior studies have shown successful decoding of joint angles in naturalistic reach-and-grasps by training state-space models based on neural activity and behavior^23,24^. As the goal of these models is to decode behavior, the state in these models is taken to be the overt behavior or to describe information about behavior such as the joint angles^23,24^. To investigate low-dimensional neural population dynamics and modes, we needed to instead build state-space models with latent states, which describe just the neural activity and are thus trained fully unsupervised with respect to behavior measurements and only on neural data. This allowed our latent state to describe the dynamics and modes in neural population activity itself. We built these models not only for single-scale spiking or LFP activity, but also for combined spike-LFP activity using an unsupervised multiscale EM learning algorithm^42^, allowing us to characterize and compare low-dimensional dynamics and modes across scales and animals. An important consequence of our work is that behavioral predictions were learned by performing a regression of the already-estimated latent state onto behavior measurements and did not involve fitting the latent state itself to the behavior.

In addition to being robustly present across experiments and animals, we found that the temporal characteristics of the multiscale predictive mode offered an explanation for how motor cortical state dynamics may control movements. A temporal characteristic of the multiscale predictive mode that supported accurate behavioral predictions was the slow, ~1 s, time decay. This conclusion is supported by convergent evidence. We identified distinct principal modes in spiking and LFP network activity other than the multiscale predictive mode (Fig. 3 and Supplementary Fig. 1). In every case, the time decay of the other principal modes was faster and so captured more transient state dynamics compared with the multiscale predictive mode (Fig. 8a). Multiscale predictive state dynamics also exhibited a ~0.2 Hz frequency component, which quantifies the tendency of the state vector to rotate (Supplementary Fig. 16). This frequency value was comparable to the behavior mode frequencies (0–0.49 Hz), though the latter had a significantly wider range and thus did not simply replicate the neural mode (Supplementary Figs. 11 and 12).

Our results suggest that relatively slow, second-long state dynamics are a reliable feature of the neural control of movement. Slow state dynamics may be specific to the control of movements for which the timing and coordination of the movements is not precisely controlled by external sensory cues. Motor processing of sensory cues likely does not feature slow state dynamics because of the importance of faithfully representing sensory input to sensation and the instructed actions. In contrast, naturalistic movements may instead evolve according to how different parts of the arm and hand are coordinated together over time, and so depend on neural computations performed across multiple brain systems, consistent with the behaviorally predictive multiscale state dynamics that we report.

We characterized the motor cortical activity with an autonomous state-space model without explicit inputs, which is also consistent with what is done in prior dynamical models of population activity^5–10,13–15^. In many experiments, inputs to the motor cortex from other regions are not observable and thus it is not easy to separate the effect of potential inputs to motor cortex when learning the model. Nevertheless, in a control analysis, we showed that even when we remove the neural activity prior to movement initiation, which is when inputs owing to presenting the movement goals or initiating the movement are prevalent^54^, the dynamical characteristics of the multiscale predictive mode remain essentially unchanged (Supplementary Fig. 8).

In addition to the shared multiscale predictive mode, there existed other distinct principal modes that were different between spiking and LFP network activity, suggesting that these distinct modes measure other neural processes present across different spatiotemporal scales. The number of distinct complex-conjugate principal modes was larger for LFP network activity compared with spiking network activity—3.1 ± 1.1 times larger in Monkey J and 3.5 ± 1.3 times larger in Monkey C (Fig. 3 and Supplementary Fig. 1). This result could reflect a larger spatial and temporal scale of LFP-based neural dynamics. Indeed, compared with fast action potential spike events that can largely be attributed to a single or at most a few local neuron sources^27,36,37^, LFP activity contains contributions from synaptic and dendritic activity as well as coordinated action potentials from a large-scale network of neurons. Thus, LFP activity may contain more diverse spatial and temporal scales of neural dynamics associated with all these contributions, leading to more principal modes. Further, as LFP activity also measures the dynamics of brain networks^27^, one possibility is that the number of principal modes in LFP activity may partially represent complementary higher-level movement information encoded in neighboring regions such as supplementary motor area^55,56^ or posterior parietal cortex^57^. Consistent with a role in movement, some principal modes that were much less informative of the low-level movement trajectories compared with the multiscale predictive mode still contained significant information about movement (Figs. 3 and 4).

An important interpretational concern is that the LFP signal recorded from an electrode may include leakage effects from the action potential events on the same electrode^58^. As spike leakage dramatically decreases with the distance between electrodes^27,36^, we formed the pool of LFP channels such that they did not overlap with the pool of spike channels to mitigate leakage concerns^27^. As the inter-electrode distance in our recording arrays was at least 1.5 mm, the non-overlapping pools of LFP and spike channels considered here should not exhibit leakage of large amplitude spikes into LFP recordings^27,59^. Moreover, leakage should largely affect the high-frequency bands of LFP activity^31,51^. However, we found that the multiscale predictive mode was present when analyzing both low-frequency and high-frequency bands of LFP activity (Results and Fig. 7).

The existence of shared multiscale behavior predictive dynamics in spiking and LFP network activity points to the importance of using LFP to enhance performance, longevity, and clinical viability of neurotechnologies. Although spiking activity is the predominant signal used to study brain mechanisms and build brain-machine interfaces (BMIs), the quality of the recorded action potentials often degrades gradually in time. LFP activity is more durable, can augment the usable lifespan of recordings and enhance future BMIs for neural decoding and stimulation^24,41,60–63^. Here, we learn the multiscale dynamics in spiking and LFP activity with a data-driven state-space model^6,18,19,21,22,42,46,47,49,53,60,62–68^ but by leveraging an unsupervised multiscale EM algorithm^42^ that can jointly model multiscale spiking and LFP activities together. Various supervised and unsupervised machine learning algorithms have been developed for state-space models but are instead designed for single-scale activity^6,18,46,47,53,60,62–65,68^. Our results motivate extending these single-scale machine learning algorithms to accommodate multiscale neural observations in the future, which can also help enhance neurotechnologies^35,41^. It is also important to investigate neural dynamics of brain signals at other spatiotemporal scales such as electroencephalogram or electrocorticogram (ECoG)^40,41,69^. Indeed, linear latent state-space models can model the network dynamics in ECoG activity^53,70^, thus motivating the extension of multiscale analyses to even larger scales.

Taken together, convergent evidence indicates that among the diversity of spatiotemporal dynamics present in brain activity, there exists a preserved multiscale low-dimensional dynamical structure that explains naturalistic reach-and-grasp movements in its generation. Furthermore, the machine learning framework we employ to reveal the multiscale predictive mode can also be used to reveal how perception, action and cognition involve multiscale brain dynamics, more generally.

## Methods

### Experimental model and subject details

We recorded neural activity from two male non-human primates (*macaca mulatta*), Monkey J and Monkey C. No statistical procedures were used to predetermine the sample size, but our sample size was similar to those reported in previous publications^9–11,19,22^. Both monkeys performed a 3D reach-and-grasp movement for liquid reward in a 50 cm × 50 cm × 50 cm workspace and continuously in time. Monkeys naturalistically reached for an object positioned on a wand, grasped the object, then released the object and returned their hand to a natural resting position (Fig. 1a). The wand was always visible to the monkeys and was continuously moved by the experimenter to random diverse locations that spanned a large spatial area in front of the monkeys (Supplementary Table 1). Also, monkeys performed the task continuously in time. Our experimental set-up allowed each monkey to choose how to reach-and-grasp the object, lacked instructions needed to isolate reach-and-grasp movement components and, in fact, lacked overt movement instructions in general (see Results). All surgical and experimental procedures were performed in compliance with the National Institute of Health Guide for Care and Use of Laboratory Animals and were approved by the New York University Institutional Animal Care and Use Committee. A total of 23 retroreflective markers were attached on each monkey’s right arm (on skin) and monitored using infrared and near-infrared motion capture cameras (Osprey Digital RealTime System, Motion Analysis Corp., USA) at a sampling rate of 100 frames s^−1^. We labeled 3D marker trajectories for each marker on the arm and hand (Cortex, Motion Analysis Corp., USA) and solved for the joint angles using a NHP musculoskeletal model^44^ that allowed us to compute inverse kinematics (SIMM, MusculoGraphics Inc., USA). The joint angles on the arm are: shoulder elevation, elevation angle, shoulder rotation, elbow flexion, pro supination, wrist flexion, and wrist deviation^44^. We tracked 20 finger joint angles for Monkey J (prox flexion, prox abduction, mid flexion, and distal flexion for each finger). For Monkey C, we tracked 18 finger joint angles as two of the finger joint angles (middle distal flexion and ring distal flexion) were missing due to technicalities. For the end-point hand kinematics, we use the position and velocity of the wrist marker in the *x*, *y*, and *z* directions. On each frame, motion capture camera data acquisition was synchronized to the neural recordings using a synchronization trigger pulse.

### Neural recordings

Spiking and LFP activity were recorded from motor cortical areas for both subjects. The recorded cortical areas were M1, PMd, PMv, and PFC for Monkey J and were PMd and PMv for Monkey C^43^. For Monkey J, an electrode array with 137 microelectrodes (large-scale micro-drive system, Gray Matter Research, USA) was used to record spike and LFP activity from the left (contralateral) hemisphere of the brain. For Monkey C, two 32-microelectrode microdrives (SC32, Gray Matter Research, USA) were used to record contralateral spiking and LFP activity. For both monkeys, broad-band (0.1 Hz–11 kHz) neural signals from all electrodes were continuously streamed to disk (30 kHz sampling rate) during task performance and synchronized with behavior. Microelectrodes were Tungsten-in-glass with impedance 0.8–1.2 MOhm (Alpha Omega).

### Neural data processing

We obtained LFP signals by applying a low-pass filter with 400 Hz cutoff frequency on raw neural signals and subsequently downsampling the signal to 1 kHz. In all our analyses, the processing time-step is set to 10 ms (denoted by **Δ**). To get the LFP features from each LFP channel, we extracted the log-power in seven frequency bands: theta (4–8 Hz), alpha (8–12 Hz), beta 1 (12–24 Hz), beta 2 (24–34 Hz), gamma 1 (34–55 Hz), gamma 2 (65–95 Hz), and gamma 3 (130–170 Hz) similar to prior works^35,45,71^. After applying common average referencing, the band power was computed from the short-time Fourier transform estimated with a 300 ms causal moving window every 50 ms. To obtain spike events, we passed the raw neural signals through a band-pass filter (0.3–6.6 kHz) and subsequently found the threshold crossing events using a threshold of 3.5 standard deviations below the mean filtered signal. Thus, in the experiment, the time-scale of spike events was 10 ms (available every time-step) and the time-scale of LFP log-power features was slower and 50 ms (available every five time steps).

### Single-scale and multiscale dynamical models

To formulate the dynamical model for neural activity, we exploit a latent state-space formulation. A state-space model consists of a state equation and an observation equation^17–19,21,22,35,42,46,47,49,53,60–63,68,70,72^. We build three dynamical models: a single-scale dynamical model for spiking activity, a single-scale dynamical model for LFP activity, and a multiscale dynamical model for combined spike-LFP activity. The state equation describes the latent state dynamics, i.e., how it evolves in time. We build the state equation for all models as

1 $${\mathbf{x}}_{t + 1} = {\mathbf{Ax}}_t + {\mathbf{w}}_t.$$

Here **x**_*t*_ is the column vector of latent states, **w**_*t*_ is a zero-mean white Gaussian noise with covariance matrix **W** and **A** is the state transition matrix ($\{ {\mathbf{x}}_t,{\mathbf{w}}_t\} \in {\Bbb R}^{n_x \times 1}$, ${\mathbf{A}} \in {\Bbb R}^{n_x \times n_x}$, and ${\mathbf{W}} \in {\Bbb R}^{n_x \times n_x}$, where *n*_*x*_ is the dimension of the latent state). The eigenvalues of the state transition matrix **A** dictate the characteristics of dynamics in the latent state. The processing time-step and thus the time-step at which the latent state is updated is 10 ms for all models.

The observation equation for spiking and LFP activity describes their relationship to the latent state. We require the observation equation to model the different statistical distributions and time-scales of spiking and LFP activity as described below. Thus, the time-scale of the observation equation is different for the three models.

We model the spiking activity of each neuron as a binary time-series. For a given neuron *c*, we denote the binary spike event at the $t^{\mathrm{th}}$ time-step by $N_t^c$, which takes a value of 1 when neuron *c* fires a spike at this time-step and a value of 0 otherwise. We use a point process to model the binary time-series of spike events^35,42,46,56,71,73–78^. Assuming neurons are conditionally independent conditioned on the latent state^35,42,46,73–77^, the observation model for the spiking network activity is given by the point process likelihood function as

2 $$p\left( {{\mathbf{N}}_t^{1:n_c}{\mathrm{|}}{\mathbf{x}}_t} \right) = \mathop {\prod }\limits_{c = 1}^{n_c} \left( {\lambda _{\mathrm{c}}\left( {\widetilde {\mathbf{x}}_t} \right){\Delta}} \right)^{N_t^c}\exp \left( { - \lambda _{\mathrm{c}}\left( {\widetilde {\mathbf{x}}_t} \right){\Delta}} \right),$$

where *n*_*c*_ is the total number of neurons and ${\mathbf{N}}_t^{1:n_c} = \left[ {N_t^1, \ldots ,N_t^{n_c}} \right]^\prime$ with $^{\prime}$ being the transpose operator. The time-step or bin-width Δ is taken to be small enough to contain at most one spike and we form $\widetilde {\mathbf{x}}_t = [1,{\mathbf{x}}_t^\prime ]^\prime$ to take into account the baseline firing rate. Here $\lambda _{\mathrm{c}}\left( {\widetilde {\mathbf{x}}_t} \right) = \exp \left( {{\mathbf{\alpha }}_c\widetilde {\mathbf{x}}_t} \right)$ is the firing rate of neuron ***c*** at time ***t***, and $\alpha _c \in {\Bbb R}^{1 \times (n_x + 1)}$ is the row vector of the point process model parameters to be learned. We take the time-scale for the spiking network activity to be 10 ms (one time-step) as we found it to be small enough to rarely contain more than one spike.

For LFP activity, prior works have shown that various frequency bands of the PSD are predictive of behavior^24,34,35,79,80^. We thus take the log-power of different bands of LFP activity as the neural features and use a linear Gaussian model to describe their relationship to the latent state as also done in prior work^24,34,35,49,53,61,62,70,79,80^. Thus, we write the observation model for the LFP network activity as

3 $${\mathbf{y}}_t = {\mathbf{C}}\widetilde {\mathbf{x}}_t + {\mathbf{r}}_t,$$

where **y**_*t*_ is the column vector of continuous LFP log-power features at time *t*, **C** is the observation matrix and **r**_*t*_ is white Gaussian noise with a covariance matrix **R** (${\mathbf{y}}_t \in {\Bbb R}^{n_y \times 1}$, ${\mathbf{C}} \in {\Bbb R}^{n_y \times (n_x + 1)}$, and ${\mathbf{R}} \in {\Bbb R}^{n_{\boldsymbol{y}} \times n_{\boldsymbol{y}}}$, where *n*_*y*_ is the number of LFP features). Thus *p*(**y**_*t*_|**x**_*t*_) in (3) is a Gaussian density with mean ${\mathbf{C}}\widetilde {\mathbf{x}}_t$ and covariance ***R***. As LFP features are computed every 50 ms, the time-scale for the LFP network activity is 50 ms in this model (i.e., they are available every five time steps).

For combined spike-LFP activity, we can write the observation model using a joint probability distribution^35,42,71,78^

4 $$p\left( {{\mathbf{N}}_t^{1:n_c},{\mathbf{y}}_t{\mathrm{|}}{\mathbf{x}}_t} \right) = p\left( {{\mathbf{N}}_t^{1:n_c}{\mathrm{|}}{\mathbf{x}}_t} \right)p\left( {{\mathbf{y}}_t{\mathrm{|}}{\mathbf{x}}_t} \right).$$

Here we have assumed that spiking and LFP activity are conditionally independent conditioned on the latent state^35,42^. The two terms in the observation equation in (4) are given by Eqs. (2) and (3), in order. The multiscale observation model has a time-scale of 10 ms for spikes and 50ms for LFPs.

Having written the state equation and the observation equations for spiking network activity, LFP network activity, and combined spike-LFP network activity, we can now construct three dynamical latent state-space models: (i) a single-scale dynamical model for spiking network activity using Eqs. (1) and (2), (ii) a single-scale dynamical model for LFP network activity using Eqs. (1) and (3), and (iii) a multiscale dynamical model for combined spike-LFP activity using Eqs. (1) and (4). Note that the latent state and parameters in each of these three models will be learned independently and with different algorithms. Thus, we emphasize that while the form and time-step of the state equation in (1) is similar, the state transition matrix ***A*** and the latent state can be completely different between the three dynamical models. Indeed, the time-scale of the observations and their statistical distribution are different in the three models. Thus, the learning and decoding algorithms to learn ***A*** and the modes and to predict the latent state in the three cases are different as described below.

### Learning algorithms for single-scale and multiscale dynamical models

To learn the single-scale and multiscale dynamical models from neural activity, we use three different EM algorithms because each of our three dynamical models involve different statistical distributions and time-scales. Let’s denote the set of spikes and LFP features in the training set by $\left\{ N \right\} = \{ N_t^c|t \le T,c = 1:n_c\}$ and $\left\{ Y \right\} = \{ {\mathbf{y}}_t|t \in {\Bbb T}_f\}$, respectively, where ${\Bbb T}_f = \left\{ {mk \le T,m \in {\Bbb N}} \right\}$ (${\Bbb N}$ is the set of positive integer numbers). Here *k* is the integer ratio representing the time-scale difference of spiking and LFP activity, i.e., the ratio of their time-scales, which is 5 here (50 ms/10 ms). *T* is the total number of time steps in the training set. For the single-scale dynamical model for spiking network activity, we use the EM algorithm for point process observations to learn the model parameters *θ*_S_ = {**A**, **W**, **x**_0_, **Λ**_0_, **α**_*c*_ for *c* = 1:*n*_*c*_}, where **x**_0_ and **Λ**_0_ are the initial estimate of the latent state and its covariance^46^. For the single-scale dynamical model with LFP network activity, we use the EM algorithm for linear Gaussian state-space models to learn the model parameters^47^ *θ*_L_ = {**A**, **W**, **x**_0_, **Λ**_0_, **C**, **R**}. To learn the novel multiscale dynamical model of combined spike-LFP activity, we employ a new multiscale EM algorithm to learn the model parameters *θ*_MS_ = {**A**, **W**, **x**_0_, **Λ**_0_, **C**, **R**, **α**_*c*_ for *c* = 1:*n*_*c*_} detailed in our prior theoretical work^42^. Note that all dynamical latent state-space models are trained just based on neural activity and unsupervised (i.e., blind) with respect to behavior measurements. Thus, the estimated latent states describe the low-dimensional dynamics in neural population activity itself and the eigenvalues of the state transition matrix ***A*** characterize the dynamical modes in neural population activity—the decay-frequency characteristics of population dynamics.

### Estimating the latent states

We first use the single-scale or multiscale EM algorithm to learn the dynamical model parameters in an unsupervised fashion—i.e., without any knowledge of behavior measurements and purely from neural activity—in the training set. We then use this model to construct the appropriate filter for estimating the latent state. For the single-scale dynamical model of spiking and LFP network activity, the optimal filters are a point process filter (PPF) and a Kalman filter (KF), respectively^41^. For the multiscale dynamical model of combined spike-LFP activity, the optimal filter is a new multiscale filter (MSF) that we have recently derived^35^. These filters estimate the latent states at each time *t*, which we denote by ***x***_*t*|*t*_.

### Predicting behavior from latent states

We learn a projection matrix from the already-estimated latent states in the training set to the measured behavior in the training set. We denote the behavior—whether joint angles or end-point hand kinematics— by **z**_*t*_ (${\mathbf{z}}_t \in {\Bbb R}^{n_b \times 1}$, n_*b*_ is the dimension of behavior). We first estimate the latent states in the training set using the filters as described in the previous section. In the training set, we then learn a matrix **L** that multiplies the already-estimated latent states to project them to the measured behavior as ${\mathbf{z}}_t = {\mathbf{L}}\widetilde {\mathbf{x}}_{t|t}$. Here $\widetilde {\mathbf{x}}_{t|t} = \left[ {1,{\mathbf{x}}_{t|t}^\prime } \right]^\prime$ to consider the bias. This constitutes a simple linear regression from the already-estimated state to behavior. Note that this linear projection is used for interpretability of dynamics, similar to prior work^19,22,42,49^. The first column of ***L*** takes into account the bias in the linear equation for behavior (${\mathbf{L}} \in {\Bbb R}^{n_b \times (n_x + 1)}$). To obtain the learned projection matrix, $\widehat {\mathbf{L}}$, we use the ordinary least-squares equation

5 $$\widehat {\mathbf{L}} = {\mathbf{Z}}\widetilde {\mathbf{X}}^\prime \left( {\widetilde {\mathbf{X}}\widetilde {\mathbf{X}}^\prime } \right)^{ - 1},$$

where, $\widetilde {\mathbf{X}} = \left[ {\widetilde {\mathbf{x}}_{1|1}, \ldots ,\widetilde {\mathbf{x}}_{t|t}, \ldots ,\widetilde {\mathbf{x}}_{T|T}} \right]$ and ${\mathbf{Z}} = [{\mathbf{z}}_{1}, \ldots , {\mathbf{z}}_{t}, \ldots , {\mathbf{z}}_{T}]$ are the matrices containing all estimated latent states and the measured behavior in the training set, respectively. We emphasize that the above projection matrix in the linear regression is learned completely separately from the dynamical latent state-space model in Eqs. (1–3), which is learned first and based only on neural activity and blind to the measured behavior. Once the state-space model is learned, it is used to estimate the latent states. These estimated latent states are then simply regressed to behavior.

Having learned the projection matrix in the training set, we predict the behavior from the already-estimated latent states. We first estimate the latent states in the test set by using the corresponding KF, PPF, or MSF from LFP network activity, spiking network activity, or their combination, respectively. We then use the linear projection matrix to predict the behavior from the estimated latent states as

6 $$\widehat {\mathbf{z}}_t = \widehat {\mathbf{L}}\widetilde {\mathbf{x}}_{t|t}.$$

To measure the prediction accuracy, we use Pearson’s CC between the true (**z**_*t*_) and predicted ($\widehat {\mathbf{z}}_t$) behavior trajectories in the test set.

*Chance-level*: it is important to emphasize that our latent state-space model consisting of Eqs. (1–3) solely describes the dynamics of neural population activity and is trained purely based on neural data and blind to behavior data. Once the state is estimated, a simple projection/regression matrix is applied on it to predict behavior as in Eq. (6). As such, the theoretical chance-level for the behavior decoding CC is the chance-level for Pearson’s CC in linear regression, which is 0. We empirically confirmed this chance-level by randomly permuting joint angles in time for the training set and the test set, relearning a new linear regression matrix from latent states to these permuted joint angles, and predicting the permuted joint angles in the test set. Repeating this procedure 1000 times, we found that the empirical chance-level was not significantly different from 0 (mean: 1.5 × 10^−4^ and std: 3.1 × 10^−3^; the mean of the distribution is not statistically different from 0, *P* = 0.51, *N*_s_ = 1100, two-sided *t* test). This is also consistent with the fact that many of our modes in Figs. 4a, b and 5a have a CC ~0, suggesting they are non-predictive. Finally, another analysis in Supplementary Fig. 4 shows that the multiscale predictive mode has significantly higher prediction CC than the CC computed by comparing the predicted joint angles in the test set to time-shifted segments of joint angles in the training set^23^.

### Extracting the modes of neural dynamics from the state equation

To extract the modes from the learned dynamical model, we first perform an eigenvalue decomposition^81^ of the state transition matrix **A** in (1) as **A** = **UEU**^−1^, where **E** is the diagonal matrix with diagonal entries equal to the eigenvalues and columns of **U** consist of the eigenvectors. Each real eigenvalue or each pair of complex-conjugate eigenvalues of **A** constitute a mode (Fig. 2b). By multiplying the state equation in (1) from the left by **U**^−1^ and defining ${\mathbf{x}}_t^{{\mathrm{modal}}} = {\mathbf{U}}^{ - 1}{\mathbf{x}}_t$, we have a transformed dynamical model in which each coordinate of the state corresponds to a single eigenvalue as ${\mathbf{x}}_t^{{\mathrm{modal}}} = {\mathbf{Ex}}_{t - 1}^{{\mathrm{modal}}} + {\mathbf{w}}_t^{{\mathrm{modal}}}$. A complex-conjugate mode corresponds to a complex-conjugate pair of eigenvalues *e*_*i*1_ and *e*_*i*2_, which are the $i_1^{{\mathrm{th}}}$ and $i_2^{{\mathrm{th}}}$ diagonal elements of **E**. Then the $i_1^{{\mathrm{th}}}$ and $i_2^{{\mathrm{th}}}$ element of the ${\mathbf{x}}_t^{{\mathrm{modal}}}$ are the associated modal coordinates of the state. Similarly, a real mode corresponds to a real eigenvalue $e_{r_1}$, which is the $r_1^{{\mathrm{th}}}$ diagonal element of **E**, with the $r_1^{{\mathrm{th}}}$ element of ${\mathbf{x}}_t^{{\mathrm{modal}}}$ being the associated modal coordinate of the state. Each mode uniquely determines how the associated modal coordinate of the state evolves in time, i.e., its dynamical characteristics. The dynamical characteristics are described by a pair of decay and frequency specifying how fast the associated modal coordinate of the state decays in time and with what frequency it rings over time in response to excitations. For a general complex-conjugate mode corresponding to eigenvalues $e_i = r_i{\mathrm{e}}^{ \pm j\theta _i}$, the decay is $\frac{{\Delta}}{{\log \big( {\frac{1}{{r_i}}} \big)}}$ and the frequency is $\frac{{\theta _i}}{{2\pi {\Delta}}}$, where Δ is the state processing time-step.

### One-step-ahead prediction of spiking and LFP network activity

To assess how well the dynamical models describe observations of spiking and LFP network activity, we compute the one-step-ahead prediction of the observations in the dynamical models. We exploit two different measures for one-step-ahead prediction of spiking and LFP activity as they have different likelihood models.

### One-step-ahead prediction of spiking network activity

For spiking activity, one-step-ahead prediction is obtained by computing the area under the curve (AUC) of the receiver operating curve (ROC)^71,82,83^ or AUC ROC. To get the ROC, we compute the predicted spiking probability from the learned dynamical model and compare it to a threshold to predict the absence or presence of the spike event (0 if probability is smaller than threshold and 1 otherwise). The predicted spiking probability of neuron *c* at time *t* given all prior spike observations is found as $\left\{ {\hat N_{{\mathrm{prob}}}^c} \right\} = \{ \lambda _c\left( {\widetilde {\mathbf{x}}_{t|t - 1}} \right){\Delta}|t \le T\}$ where $\widetilde {\mathbf{x}}_{t|t - 1} = \left[ {1,{\mathbf{x}}_{t|t - 1}^\prime } \right]^\prime$ and **x**_*t*|*t*−1_ is the one-step-ahead prediction of latent states in the test set given all prior spike observations $\left\{ {N^c} \right\} = \{ N_i^c|i \le t - 1\}$ obtained from the filter (PPF or MSF). We subsequently compute the true positive rate and false positive rate for the range of all possible thresholds from 0 to 1, and plot these rates against each other. We quantify the one-step-ahead prediction accuracy of the spiking network activity using prediction power for all *n*_*c*_ neurons denoted by **PP**_spike_, which is a column vector (${\mathbf{PP}}_{{\mathrm{spike}}} \in {\Bbb R}^{n_c \times 1}$). For a given neuron *c*, the prediction power is the $c^{\mathrm{th}}$ component of this vector given by^71,82,83^

7 $${\mathbf{PP}}_{{\mathrm{spike}}}\left( c \right) = 2 \times {\mathrm{AUC}}\,{\mathrm{ROC}}\left( {\left\{ {\widehat N_{{\mathrm{prob}}}^c} \right\}} \right) - 1.$$

For the spiking network activity, we use mean of the **PP**_spike_ as the measure. Each element of **PP**_spike_ is between 0 and 1 and thus better one-step-ahead prediction results in a higher prediction power measure closer to 1, which corresponds to perfect prediction.

### One-step-ahead prediction of LFP network activity

For LFP network activity, we use one-step-ahead prediction accuracy as measured by Pearson’s CC. We denote the one-step-ahead prediction accuracy of LFP network activity by **CC**_LFP_ as a column vector containing the one-step-ahead prediction CC of all *n*_*y*_ LFP features, i.e., ${\mathbf{CC}}_{{\mathrm{LFP}}} \in {\Bbb R}^{n_y \times 1}$. For the $j^{\mathrm{th}}$ LFP feature, we define:

8 $${\mathbf{CC}}_{{\mathrm{LFP}}}\left( j \right) = {\mathbf{CC}}_j\left( {\left\{ {\widehat Y} \right\},\left\{ Y \right\}} \right),$$

where $\{ \widehat Y\} = \{ \widehat {\mathbf{y}}_{t|t - 1} = {\mathbf{C}}\widetilde {\mathbf{x}}_{t|t - 1}|t \in {\Bbb T}_{\mathrm{f}}\}$ and $\{ Y\} = \{ {\mathbf{y}}_t|t \in {\Bbb T}_{\mathrm{f}}\}$. Thus the CC_*j*_ measure for the $j^{\mathrm{th}}$ LFP feature is the CC between its true (${\mathbf{y}}_t^j$) and its one-step-ahead predicted ($\widehat {\mathbf{y}}_{t|t - 1}^j$) time-series. Our overall one-step-ahead prediction measure for the LFP network activity is then defined as the mean of **CC**_LFP_.

### Quantifying the contribution of modes to behavior and one-step-ahead neural prediction

We can write any arbitrary linear projection **F** of the latent states as

9 $${\mathbf{Fx}}_t = {\mathbf{FUx}}_t^{{\mathrm{modal}}} = \mathop {\sum }\limits_{i = 1}^{n_x} \left[ {{\mathrm{col}}_i\left( {{\mathbf{FU}}} \right){\mathrm{row}}_i\left( {{\mathbf{x}}_t^{{\mathrm{modal}}}} \right)} \right],$$

where row_*i*_(·) and col_*i*_(⋅) denote the $i^{\mathrm{th}}$ row and $i^{\mathrm{th}}$ column of a matrix/vector, respectively. *n*_*x*_ is the dimension of the latent state. Therefore, any linear transform of the latent state can be written as a linear sum of the contribution of its modal coordinates corresponding to all modes. For example, the contribution of the $i^{\mathrm{th}}$ modal coordinate of the state is ${\mathrm{col}}_i\left( {{\mathbf{FU}}} \right){\mathrm{row}}_i\left( {{\mathbf{x}}_t^{{\mathrm{modal}}}} \right)$. We refer to this as the $i^{\mathrm{th}}$ mode contribution; note that for a complex mode, its contribution is the sum of the contributions of the associated pair of complex-conjugate eigenvalues. With this procedure, we can dissociate the contribution of mode *i* in Eq. (6) to behavior prediction in the test set, which we denote by $\widehat {\mathbf{z}}_{i,t}$ (see Supplementary Note 1). Similarly, we can compute the contribution of mode *i* to one-step-ahead prediction of LFP activity and spiking activity in the test set, respectively (see Supplementary Note 1).

### Evaluation using fivefold cross-validation

For all analyses in this work, we performed fivefold cross-validation, where we divide the data into five equally sized folds and use four folds as the training set to learn the model and leave one fold out for the test set to evaluate the accuracy of behavior prediction and one-step-ahead prediction of neural activity.

### Forming the pool of spike and LFP channels for analyses

To mitigate leakage effects, we constructed non-overlapping pools of spike and LFP channels to make sure they have no common recording electrode between them. To do so, we first selected a pool of 30 recording channels whose spiking activity we analyzed (termed spike channels). Then, from the remaining recording channels, we picked 30 recording electrodes whose LFP activity we analyzed (termed LFP channels). We selected the LFP channels whose single-channel LFP behavior prediction accuracy was similar to the single-channel spike behavior prediction accuracy in the pool of spike channels.

In a control analysis, we also examined what happens if the LFP channels are randomly selected from among the remaining channels that are non-overlapping with spike channels. To do so, within a new Monte Carlo bootstrap procedure, we randomly sampled 30 LFP channels from the remaining channels. We repeated the procedure 30 times and used the corrected resampled *t* test to assess significance^84^.

### Investigating the modes of spiking and LFP network activity: eigenvalue-dimension diagram

We ran a modal analysis to find the principal modes in the spiking and LFP network activity. To this end, we learned single-scale and multiscale dynamical models with various latent state dimensions, in particular sweeping this dimension from 1 to 25 in the single-scale and multiscale EM algorithms. Principal modes in the network activity are expected to be learned with consistent dynamical characteristics starting from some low dimension. Therefore, we generate a diagram in which the *x* and *y* axes represent the real and imaginary components of the modes, respectively, and the *z* axis represents the dimension of the latent state (i.e., from 1 to 25). For complex-conjugate modes, we just show the eigenvalue with positive imaginary component in the pair without loss of information. We call this diagram the eigenvalue-dimension diagram. The distance of two modes is defined as the norm of the difference between their eigenvalues, which is equivalent to the Euclidean distance in the *x*–*y* plane of the eigenvalue-dimension diagram. Principal modes should appear as condensed vertical clusters of modes in the eigenvalue-dimension diagram. We define a condensed cluster of modes as a vertical cluster in which the distance of the members from the cluster centroid is significantly smaller than half of chance-level. We use the half ratio to make sure that the modes are located around the centroid within a circle whose diameter is less than chance-level.

After generating the eigenvalue-dimension diagram, we cluster the modes using K-means clustering (see below). We next compute the contribution of each mode cluster to behavior prediction and one-step-ahead prediction of neural activity. We finally compare the dynamical characteristics of these mode clusters across scales, experimental sessions and monkeys.

### K-means clustering of modes

We performed K-means to cluster the modes present in the network activity. To do this, after forming the non-clustered eigenvalue-dimension diagram, we first project all the modes onto the *x*−*y* plane. We then cluster the modes using K-means clustering. As complex conjugate and real modes have fundamentally different properties, we cluster them separately. The number of complex-conjugate modes converged for dimensions higher than 10 (Fig. 3 and Supplementary Fig. 1). Thus, for complex-conjugate modes, we set the number of clusters to the median of the number of complex-conjugate modes learned in dynamical models with order higher than 10. For real modes, we fix the number of clusters to be 4 as these modes are very weak in behavior and neural prediction and also very fast decaying and transient, and thus their more detailed clustering does not change any results or conclusions (Figs. 3 and 4, Supplementary Fig. 3). We next find the cluster centroids using 1D and 2D K-means clustering for real and complex-conjugate modes, respectively. After finding the cluster centroids, we find the closest cluster centroid for each mode and assign the mode to that cluster. To remove the modes that are simply learned owing to noise, for example, appear just at one dimension along the *z* axis, we discard modes that significantly deviate from the all cluster centroids. To do this, we compute the decay and frequency of each mode in the cluster and if either the relative frequency difference $\left( {\frac{{\left| {{\mathrm{frequnecy}}_{{\mathrm{mode}}} - {\mathrm{frequency}}_{{\mathrm{centroid}}}} \right|}}{{{\mathrm{frequency}}_{{\mathrm{centroid}}}}}} \right)$ or the relative decay difference $\left( {\frac{{\left| {{\mathrm{decay}}_{{\mathrm{mode}}} - {\mathrm{decay}}_{{\mathrm{centroid}}}} \right|}}{{{\mathrm{decay}}_{{\mathrm{centroid}}}}}} \right)$ is >50%, that mode is discarded. After finding all the memberships and cluster centroids, we plot the clustered eigenvalue-dimension diagram (see Fig. 2c).

### Monte Carlo bootstrap modal analyses when combining spike-LFP activity

For the Monte Carlo bootstrap analysis and to have more samples, we created channel sets from the non-overlapping pool of spike and LFP channels. For each experimental session (seven for Monkey J and four for Monkey C), we formed a channel set by randomly selecting five baseline spike channels and 25 additional randomly drawn non-overlapping LFP channels. We also formed channel sets containing five LFP channels and 25 non-overlapping spike channels. For each case, we repeated the process 20 times to obtain the Monte Carlo bootstrap channel sets. In total, we randomly created 280 and 160 channel sets for Monkey J and C, respectively.

Within this Monte Carlo bootstrap set-up, we examine how the prediction accuracy and distance of the estimated modes to the multiscale predictive mode change when combining spiking and LFP activity compared with when using them separately. To this end, we obtain the modes from the single-scale dynamical models learned from five baseline spike or LFP channels in each channel set. We next obtain the modes in multiscale dynamical models learned from combined spike-LFP activity of the channel set. For computational tractability, we fix the dimension of the latent state in the dynamical model to be 15. For each channel set and training fold, we compute the distance of the closest estimated mode to the multiscale predictive mode and its cross-validated prediction accuracy, performed for both separate and combined spike-LFP activity (Results). We set the ground-truth location of the multiscale predictive mode in this analysis as the mean of predictive mode centroids in spiking and LFP network activity when using all channels (mean of yellow and brown dots in Fig. 5b). After combining spiking and LFP activity, we compute: 1) how much the distance to the multiscale predictive mode improved (positive if distance reduced and negative if distance increased) and 2) the improvement in the estimated mode’s prediction accuracy. We then test whether the distance to the multiscale predictive mode reduced and whether this decrease was correlated with the improvement in prediction accuracy. To this end, we first *z* score the changes in distance and prediction accuracy in each experimental session, then pool them across experimental sessions and find the linear least-square fit between them.

For our control analyses, we formed 280 and 160 channel sets for Monkey J and C, where we now picked 10 spike or LFP channels as baseline single-scale channels instead of 5. We repeated the process above for these channel sets in Supplementary Fig. 14.

### Analysis of modes in frequency bands of LFP activity

To test whether the predictive mode is present in low-frequency or high-frequency bands of LFP activity, we repeated the modal analysis for different frequency band combinations of LFP activity. We use theta + alpha + beta as the low-frequency bands and gamma (gamma 1 + gamma 2 + gamma 3) as the high-frequency band of LFP activity. In addition to these 2 band combinations, we also consider four additional combinations of frequency bands of LFP activity: theta + alpha, beta, theta + alpha + gamma, and beta + gamma. To perform the analysis, for each experimental session and each frequency band combination, we obtain the eigenvalue-dimension diagram of the dynamical models learned from the frequency band combination. We then find the closest mode cluster to the multiscale predictive mode. We set the ground-truth location of this multiscale predictive mode as the mean of the predictive mode centroids over experimental sessions in spiking network activity shown in Fig. 5b (when all spike channels are used); this ensures that the location of the predictive mode is not dictated by any of LFP frequency bands to avoid confounding the results of the LFP frequency band analysis (Results and Fig. 5b). We then find the closest mode to the multiscale predictive mode for every frequency band combination, and find the cross-validated prediction accuracy of this closest mode and its distance to the multiscale predictive mode.

To test whether the predictive mode exists in both low- and high-frequency bands, we found the distances between the multiscale predictive mode and the estimated mode clusters in the low- and high-frequency bands and compared these distances with chance-level. We then tested the hypothesis that the closest estimated mode cluster’s prediction accuracy is correlated with its distance to the multiscale predictive mode. To this end, we pooled the estimated mode prediction accuracies (covariate 1) and their distances to the multiscale predictive mode (covariate 2) across all experimental sessions and all combinations. Similar to our Monte Carlo bootstrap analysis, we *z* scored both covariates in each experimental session, then pooled the *z* scored values across experimental sessions and found the linear least-square fit between the covariates.

### MVLR of behavior prediction accuracy versus decay and frequency deviation

We employed MVLR to study the effect of frequency and decay of the multiscale predictive mode on its behavior prediction accuracy. In the MVLR model, the dependent variable was behavior prediction accuracy and the independent variables were decay deviation and frequency deviation; decay deviation was computed as the absolute difference of each mode’s decay from the multiscale predictive mode’s decay, and frequency deviation was computed similarly. The multiscale predictive mode’s decay and frequency were set to the mean decay and mean frequency of the predictive modes across scales and experimental sessions (mean decay/frequency of all yellow and brown dots in Fig. 5b). Thus, the MVLR equation was ${\mathrm{behavior}}\,{\mathrm{prediciton}}\,{\mathrm{accuracy}} \sim a_0 + a_1 \times {\mathrm{frequency}}\,{\mathrm{deviation}} + b_1 \times {\mathrm{decay}}\,{\mathrm{deviation}}$. We performed MVLR for all the complex-conjugate principal modes after removing outliers using median absolute deviation method as MVLR is sensitive to outliers (only one mode was removed). In order to rule out the multicollinearity, we computed the variance inflation factor (VIF) between decay deviation and frequency deviation, which was 1.89. VIF provides a measure of how much the variance of the estimated coefficients are increased because of collinearity. As a rule of thumb VIF > 5 indicates a problematic collinearity, which does not apply to our case. We then investigated the fitted line, and the fitted MVLR coefficients and their significance.

### Perturbing the decay and frequency of the multiscale predictive mode

We recomputed the prediction accuracies for a perturbed version of the multiscale predictive mode to study the effects of its decay and frequency. To study the effect of decay, in each experimental session, we fixed the frequency of the multiscale predictive mode to its mean value across sessions (mean frequency of all yellow and brown dots in Fig. 5b) while perturbing its decay from 0.1−10 s. We kept all the other learned modes intact. The perturbed mode and the other fixed modes specified the state transition matrix (**A**). We then relearned the other state-space parameters by fixing the **A** to be this perturbed matrix in the maximization step of the EM algorithm while only updating the other parameters. We similarly studied the effect of frequency by fixing the decay of the multiscale predictive mode to its mean value across sessions and perturbing its frequency from 0.02 to 3 Hz. For simplicity, we fixed the dimension of the latent state to be 15. By repeating the prediction analyses, we asked whether the frequency and decay of the multiscale predictive mode were important in explaining behavior prediction accuracy.

### Statistical analysis

For all analysis in this work, significance was declared if *P* < 0.05 (after multiple comparison correction with FDR if needed). The Benjamini–Hochberg FDR is used to correct for multiple comparisons, i.e., the increased likelihood of rejecting the null hypothesis owing to multiple comparisons. All statistical tests other than the Monte Carlo bootstrap analysis tests are performed with non-parametric Wilcoxon signed-rank test for paired and non-parametric Wilcoxon rank sum tests for non-paired samples. However, we also performed a control analysis with the corrected resampled *t* test^84^ for these tests and found that our statistical significance results remained unchanged. For the Monte Carlo bootstrap analysis, statistical tests are performed and reported with the corrected resampled *t* test.

### Reporting summary

Further information on research design is available in the Nature Research Reporting Summary linked to this article.

## Supplementary information

Supplementary Information

Reporting Summary

## Data Availability

The data used to support the findings of this study are available within the article, its Supplementary Information files, the Source Data file or upon reasonable request from the corresponding author. Source data are provided with this paper.

## Source data

Source Data

## Code availability

The custom computer code used to support the results is available at https://github.com/ShanechiLab/multiscaleEM.

## References

[1] Georgopoulos AP, Kalaska JF, Caminiti R, Massey JT (1982). On the relations between the direction of two-dimensional arm movements and cell discharge in primate motor cortex. J. Neurosci..

[2] Moran DW, Schwartz AB (1999). Motor cortical representation of speed and direction during reaching. J. Neurophysiol..

[3] Fetz EE (1992). Are movement parameters recognizable coded in the activity of single neurons?. Behav. Brain Sci..

[4] Reimer, J. & Hatsopoulos, N. G. *The Problem of Parametric Neural Coding in the Motor System. Advances in Experimental Medicine and Biology.* 629, 243–259 (Springer, Boston, MA, 2009). . 10.1007/978-0-387-77064-2_12 PMC4480635 19227503

[5] Yu BM (2009). Gaussian-process factor analysis for low-dimensional single-trial analysis of neural population activity. J. Neurophysiol..

[6] Macke JH (2011). Empirical models of spiking in neuronal populations. Adv. Neural Inf. Process. Syst. NIPS.

[7] Churchland MM (2012). Neural population dynamics during reaching. Nature.

[8] Hall TM, deCarvalho F, Jackson A (2014). A common structure underlies low-frequency cortical dynamics in movement, sleep, and sedation. Neuron.

[9] Sadtler PT (2014). Neural constraints on learning. Nature.

[10] Vaidya M, Kording K, Saleh M, Takahashi K, Hatsopoulos NG (2015). Neural coordination during reach-to-grasp. J. Neurophysiol..

[11] Sussillo D, Churchland MM, Kaufman MT, Shenoy KV (2015). A neural network that finds a naturalistic solution for the production of muscle activity. Nat. Neurosci..

[12] Michaels JA, Dann B, Scherberger HH (2016). Neural population dynamics during reaching are better explained by a dynamical system than representational tuning. PLoS Comput. Biol..

[13] Gallego JA (2018). Cortical population activity within a preserved neural manifold underlies multiple motor behaviors. Nat. Commun..

[14] Golub MD (2018). Learning by neural reassociation. Nat. Neurosci..

[15] Pandarinath C (2018). Inferring single-trial neural population dynamics using sequential auto-encoders. Nat. Methods.

[16] Susilaradeya, D. et al. Extrinsic and intrinsic dynamics in movement intermittency. *eLife* **8**, e40145 (2019). 10.7554/eLife.40145 PMC6453565 30958267

[17] Cunningham JP, Yu BM (2014). Dimensionality reduction for large-scale neural recordings. Nat. Neurosci..

[18] Petreska, B. et al. Dynamical segmentation of single trials from population neural data. *Adv. Neural Inf. Process. Syst*. **24**, 756–764 (2011).

[19] Kao JC (2015). Single-trial dynamics of motor cortex and their applications to brain-machine interfaces. Nat. Commun..

[20] Michaels JA, Dann B, Intveld RW, Scherberger H (2015). Predicting reaction time from the neural state space of the premotor and parietal grasping network. J. Neurosci..

[21] Aghagolzadeh M, Truccolo W (2016). Inference and decoding of motor cortex low-dimensional dynamics via latent state-space models. IEEE Trans. Neural Syst. Rehabil. Eng..

[22] Kao JC, Ryu SI, Shenoy KV (2017). Leveraging neural dynamics to extend functional lifetime of brain-machine interfaces. Sci. Rep..

[23] Vargas-Irwin CE (2010). Decoding complete reach and grasp actions from local primary motor cortex populations. J. Neurosci..

[24] Bansal AK, Truccolo W, Vargas-Irwin CE, Donoghue JP (2012). Decoding 3D reach and grasp from hybrid signals in motor and premotor cortices: spikes, multiunit activity, and local field potentials. J. Neurophysiol..

[25] Schoffelen JM, Oostenveld R, Fries P (2005). Neuronal coherence as a mechanism of effective corticospinal interaction. Science.

[26] Bressler SL, Menon V (2010). Large-scale brain networks in cognition: emerging methods and principles. Trends Cogn. Sci..

[27] Pesaran, B. et al. Investigating large-scale brain dynamics using field potential recordings: analysis and interpretation. *Nat. Neurosci*. **21**, 903–919 (2018). 10.1038/s41593-018-0171-8 PMC7386068 29942039

[28] Pesaran B, Pezaris JS, Sahani M, Mitra PP, Andersen RA (2002). Temporal structure in neuronal activity during working memory in macaque parietal cortex. Nat. Neurosci..

[29] Mehring C (2003). Inference of hand movements from local field potentials in monkey motor cortex. Nat. Neurosci..

[30] Berens P, Keliris GA, Ecker AS, Logothetis NK, Tolias AS (2008). Comparing the feature selectivity of the gamma-band of the local field potential and the underlying spiking activity in primate visual cortex. Front. Syst. Neurosci..

[31] Belitski A, Panzeri S, Magri C, Logothetis NK, Kayser C (2010). Sensory information in local field potentials and spikes from visual and auditory cortices: time scales and frequency bands. J. Comput. Neurosci..

[32] Flint RD, Lindberg EW, Jordan LR, Miller LE, Slutzky MW (2012). Accurate decoding of reaching movements from field potentials in the absence of spikes. J. Neural Eng..

[33] Perel S (2015). Single-unit activity, threshold crossings, and local field potentials in motor cortex differentially encode reach kinematics. J. Neurophysiol..

[34] Stavisky SD, Kao JC, Nuyujukian P, Ryu SI, Shenoy KV (2015). A high performing brain-machine interface driven by low-frequency local field potentials alone and together with spikes. J. Neural Eng..

[35] Hsieh, H.-L. L., Wong, Y. T., Pesaran, B. & Shanechi, M. M. Multiscale modeling and decoding algorithms for spike-field activity. *J. Neural Eng*. **16**, 016018 (2018). 10.1088/1741-2552/aaeb1a 30523833

[36] Buzsáki G, Anastassiou CA, Koch C (2012). The origin of extracellular fields and currents-EEG, ECoG, LFP and spikes. Nat. Rev. Neurosci..

[37] Einevoll GT, Kayser C, Logothetis NK, Panzeri S (2013). Modelling and analysis of local field potentials for studying the function of cortical circuits. Nat. Rev. Neurosci..

[38] Scherberger H, Jarvis MR, Andersen RA (2005). Cortical local field potential encodes movement intentions in the posterior parietal cortex. Neuron.

[39] Heldman DA, Hokanson JA, Moran DW (2006). Local field potential spectral tuning in primary motor cortex. Soc. Neurosci. Abstr..

[40] Lebedev MA, Nicolelis MAL (2006). Brain-machine interfaces: past, present and future. Trends Neurosci..

[41] Shanechi MM (2019). Brain-machine interfaces from motor to mood. Nat. Neurosci..

[42] Abbaspourazad H, Hsieh H-LL, Shanechi MM (2019). A multiscale dynamical modeling and identification framework for spike-field activity. IEEE Trans. Neural Syst. Rehabil. Eng..

[43] Wong, Y. T., Putrino, D., Weiss, A. & Pesaran, B. Utilizing movement synergies to improve decoding performance for a brain machine interface. *Conf. IEEE Eng. Med. Biol. Soc. EMBS* 2013, 289–292 (2013). 10.1109/EMBC.2013.6609494 PMC4180097 24109681

[44] Putrino D, Wong YT, Weiss A, Pesaran B (2015). A training platform for many-dimensional prosthetic devices using a virtual reality environment. J. Neurosci. Methods.

[45] Bundy DT, Pahwa M, Szrama N, Leuthardt EC (2016). Decoding three-dimensional reaching movements using electrocorticographic signals in humans. J. Neural Eng..

[46] Smith AC, Brown EN (2003). Estimating a state-space model from point process observations. Neural Comput..

[47] Ghahramani Z (1996). Parameter estimation for linear dynamical systems. Tech. Rep..

[48] He, J. & Fu, Z. *Modal analysis*. *Environmental Fluid Dynamics* (2013). 10.1016/B978-0-12-088571-8.01001-9.

[49] Sani OG (2018). Mood variations decoded from multi-site intracranial human brain activity. Nat. Biotechnol..

[50] Cisek P, Kalaska JF (2005). Neural correlates of reaching decisions in dorsal premotor cortex: specification of multiple direction choices and final selection of action. Neuron.

[51] Ray S, Crone NE, Niebur E, Franaszczuk PJ, Hsiao SS (2008). Neural correlates of high-gamma oscillations (60–200 Hz) in macaque local field potentials and their potential implications in electrocorticography. J. Neurosci. J. Soc. Neurosci..

[52] Pastor-Bernier, A., Tremblay, E. & Cisek, P. Dorsal premotor cortex is involved in switching motor plans. *Front. Neuroeng.* **5**, 5 (2012). 10.3389/fneng.2012.00005 PMC3318308 22493577

[53] Ahmadipour, P., Yang, Y., Chang, E. F. & Shanechi, M. M. Adaptive tracking of human ECoG network dynamics. *J. Neural Eng.* https://doi.org/10.1088/1741-2552/abae42 (2020). 10.1088/1741-2552/abae42 33624610

[54] Wise SP, Boussaoud D, Johnson PB, Caminiti R (1997). PREMOTOR AND PARIETAL CORTEX: corticocortical connectivity and combinatorial computations. Annu. Rev. Neurosci..

[55] Nachev P, Kennard C, Husain M (2008). Functional role of the supplementary and pre-supplementary motor areas. Nat. Rev. Neurosci..

[56] Shanechi MM (2012). Neural population partitioning and a concurrent brain-machine interface for sequential motor function. Nat. Neurosci..

[57] Batista AP, Andersen RA (2001). The parietal reach region codes the next planned movement in a sequential reach task. J. Neurophysiol..

[58] Waldert S, Lemon RN, Kraskov A (2013). Influence of spiking activity on cortical local field potentials. J. Physiol..

[59] Katzner S (2009). Local origin of field potentials in visual cortex. Neuron.

[60] Bolus, M. F., Willats, A. A., Whitmire, C. J., Rozell, C. J. & Stanley, G. B. Design strategies for dynamic closed-loop optogenetic neurocontrol in vivo. *J. Neural Eng*. **15**, 026011 (2018). 10.1088/1741-2552/aaa506 PMC5957547 29300002

[61] Yang Y, Connolly AT, Shanechi MM (2018). A control-theoretic system identification framework and a real-time closed-loop clinical simulation testbed for electrical brain stimulation. J. Neural Eng..

[62] Yang, Y. et al. Modelling and prediction of the dynamic responses of large-scale brain networks during direct electrical stimulation. *Nat. Biomed. Eng*. (in press). 10.1038/s41551-020-00666-w 33526909

[63] Sani, O. G., Abbaspourazad, H., Wong, Y. T., Pesaran, B. & Shanechi, M. M. Modeling behaviorally relevant neural dynamics enabled by preferential subspace identification. *Nat. Neurosci*. 1–10 (2020) 10.1038/s41593-020-00733-0. 10.1038/s41593-020-00733-0 33169030

[64] Wu W, Kulkarni JE, Hatsopoulos NG, Paninski L (2009). Neural decoding of hand motion using a linear state-space model with hidden states. IEEE Trans. Neural Syst. Rehabil. Eng..

[65] Charles, A., Asif, M. S., Romberg, J. & Rozell, C. Sparsity penalties in dynamical system estimation. in *2011 45th Annual Conference on Information Sciences and Systems, CISS 2011* (2011) 10.1109/CISS.2011.5766179.

[66] Millard DC, Wang Q, Gollnick CA, Stanley GB (2013). System identification of the nonlinear dynamics in the thalamocortical circuit in response to patterned thalamic microstimulation in vivo. J. Neural Eng..

[67] Hsieh H-L, Shanechi MM (2018). Optimizing the learning rate for adaptive estimation of neural encoding models. PLoS Comput. Biol..

[68] Yang Y (2019). Developing a personalized closed-loop controller of medically-induced coma in a rodent model. J. Neural Eng..

[69] Lee, D. K. et al. Neural encoding and production of functional morphemes in the posterior temporal lobe. *Nat. Commun*. **9**, 1877 (2018). 10.1038/s41467-018-04235-3 PMC5951905 29760465

[70] Yang Y, Sani O, Chang EF, Shanechi MM (2019). Dynamic network modeling and dimensionality reduction for human ECoG activity. J. Neural Eng..

[71] Bighamian R, Wong YT, Pesaran B, Shanechi MM (2019). Sparse model-based estimation of functional dependence in high-dimensional field and spike multiscale networks. J. Neural Eng..

[72] Yang Y, Shanechi MM (2016). An adaptive and generalizable closed-loop system for control of medically induced coma and other states of anesthesia. J. Neural Eng..

[73] Shanechi MM, Orsborn AL, Carmena JMJM (2016). Robust brain-machine interface design using optimal feedback control modeling and adaptive point process filtering. PLoS Comput. Biol..

[74] Eden UT, Frank LM, Barbieri R, Solo V, Brown EN (2004). Dynamic analysis of neural encoding by point process adaptive filtering. Neural Comput.

[75] Truccolo W (2005). A point process framework for relating neural spiking activity to spiking history, neural ensemble, and extrinsic covariate effects. J. Neurophysiol..

[76] Shanechi MM (2017). Rapid control and feedback rates enhance neuroprosthetic control. Nat. Commun..

[77] Sadras N, Pesaran B, Shanechi MM (2019). A point-process matched filter for event detection and decoding from population spike trains. J. Neural Eng..

[78] Wang C, Shanechi MM (2019). Estimating multiscale direct causality graphs in neural spike-field networks. IEEE Trans. Neural Syst. Rehabil. Eng..

[79] Markowitz DA, Wong YT, Gray CM, Pesaran B (2011). Optimizing the decoding of movement goals from local field potentials in macaque cortex. J. Neurosci..

[80] So K, Dangi S, Orsborn AL, Gastpar MC, Carmena JM (2014). Subject-specific modulation of local field potential spectral power during brain{-}machine interface control in primates. J. Neural Eng..

[81] Peeters B, De Roeck G (1999). Reference-based stochastic subspace identification for output-only modal analysis. Mech. Syst. Signal Process..

[82] Truccolo W, Hochberg LR, Donoghue JP (2009). Collective dynamics in human and monkey sensorimotor cortex: predicting single neuron spikes. Nat. Neurosci..

[83] Rule ME, Vargas-Irwin C, Donoghue JP, Truccolo W (2015). Contribution of LFP dynamics to single-neuron spiking variability in motor cortex during movement execution. Front. Syst. Neurosci..

[84] Nadeau C, Bengio Y (2003). Inference for the generalization error. Mach. Learn..

