# Supplementary material for: Multiscale low-dimensional motor cortical state dynamics predict naturalistic reach-and-grasp behavior
Source: Nat Commun. 2021 Jan 27;12:607. doi: 10.1038/s41467-020-20197-x (PMC7840738; doi:10.1038/s41467-020-20197-x)
Supplement: Supplementary file 1 — Supplementary Information [file 41467_2020_20197_MOESM1_ESM.pdf]

Supplementary information

Multiscale low-dimensional motor cortical state dynamics predict naturalistic reach-and-grasp behavior

Hamidreza Abbaspourazad[1], Mahdi Choudhary[2], Yan Tat Wong[3], Bijan Pesaran[2] and Maryam M. Shanechi[1, 4, 5 *]

[1]Ming Hsieh Department of Electrical and Computer Engineering, Viterbi School of Engineering, University of Southern California, Los Angeles, CA 90089 USA.

[2]Center for Neural Science, New York University, New York, NY 10003 USA.

[3]Department of Physiology, Electrical and Computer Systems Engineering, Monash University, VIC 3800 Australia.

[4]Neuroscience Graduate Program, University of Southern California, Los Angeles, CA 90089 USA.

[5]Department of Biomedical Engineering, University of Southern California, Los Angeles, CA 90089 USA.

*Corresponding Author: shanechi@usc.edu

Supplementary Figures

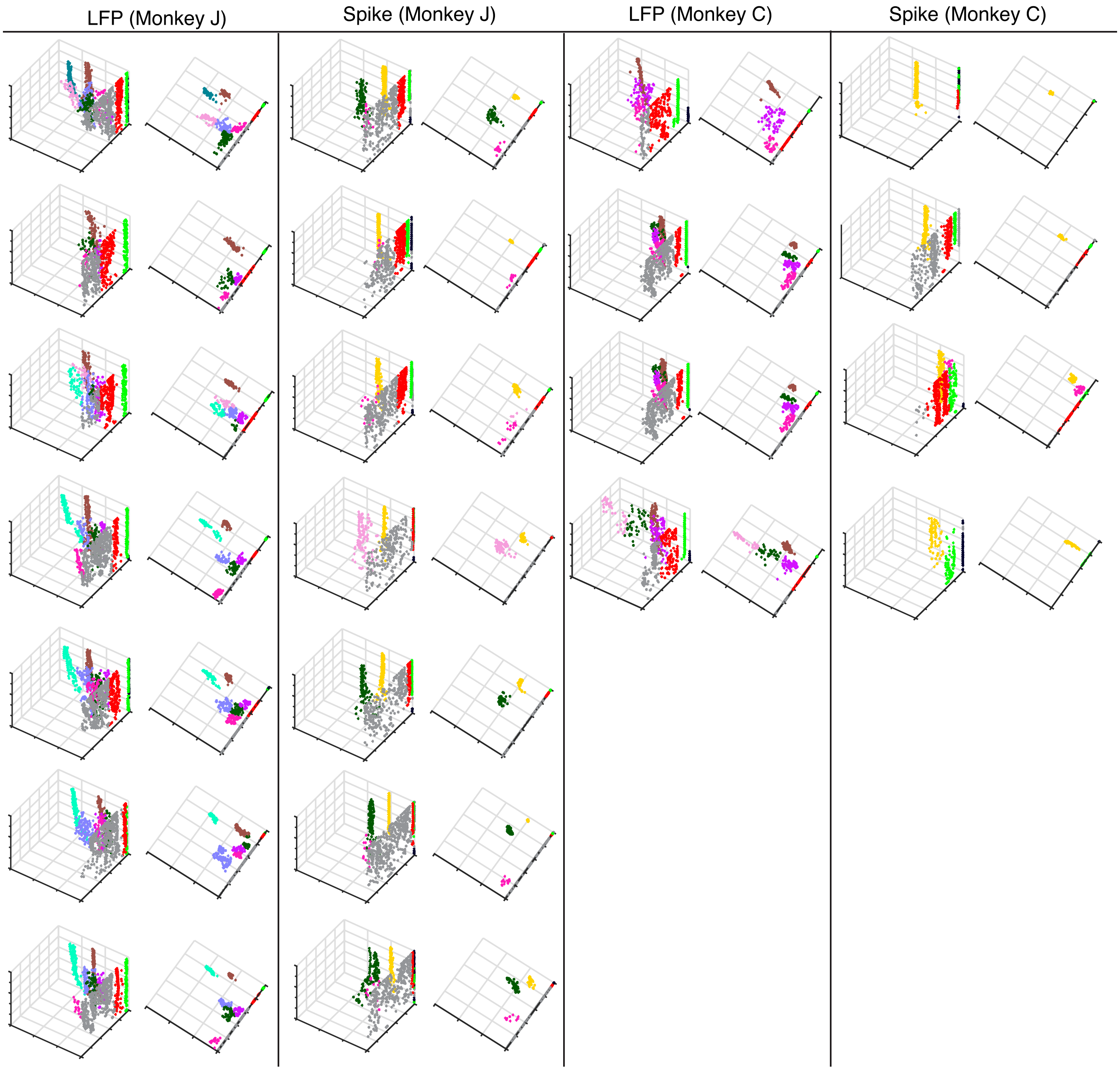

Supplementary Figure 1. Eigenvalue-dimension diagram for spiking and LFP network activity for all experimental sessions. Axes ranges and labels are similar to **Fig. 3**. The predictive mode is shown in yellow for spiking network activity and in brown for LFP network activity. Each row corresponds to one experimental session for monkey J (left two columns) and Monkey C (right two columns). Note that the

color of the modes other than the predictive mode is arbitrarily assigned in each session. Source data are provided as a Source Data file.

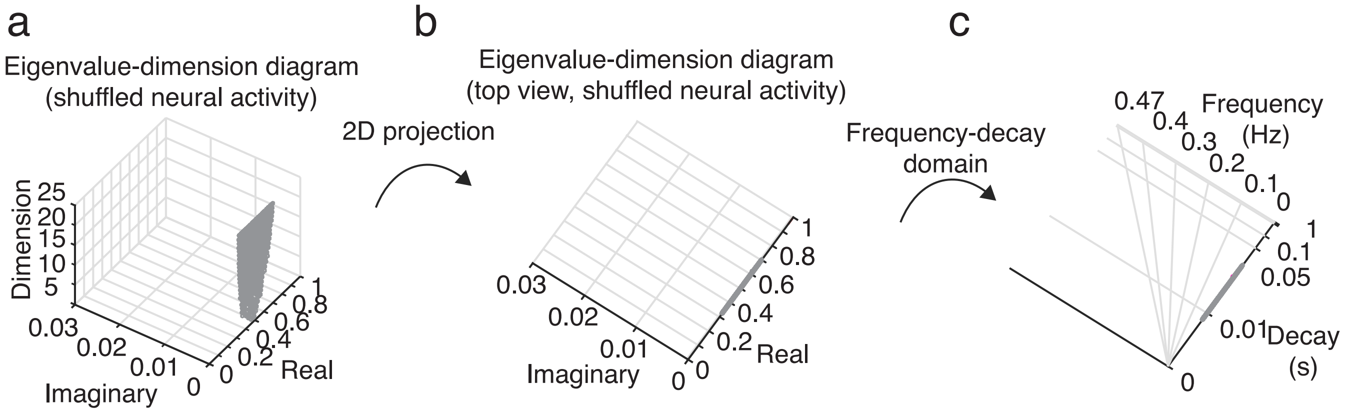

Supplementary Figure 2. Shuffled neural activity did not exhibit any complex conjugate principal modes unlike spiking and LFP network activity in our data. **a** Eigenvalue-dimension diagram for the shuffled neural activity for the sample session shown in **Fig. 3**. Shuffled neural activity did not have any complex conjugate modes and the range was very far from the range of neural modes in our data. **b** The top view of eigenvalue-dimension diagram is shown. **c** For interpretability, the frequency-decay domain is shown. The range of the decay is around 0.01 to 0.05 s, which is significantly faster decaying compared to the multiscale predictive mode decays (~1 s). Thus, unlike spiking and LFP activity, modes of shuffled activity decayed quickly in time. Grey lines show the contours on which frequency or decay is constant. Source data are provided as a Source Data file.

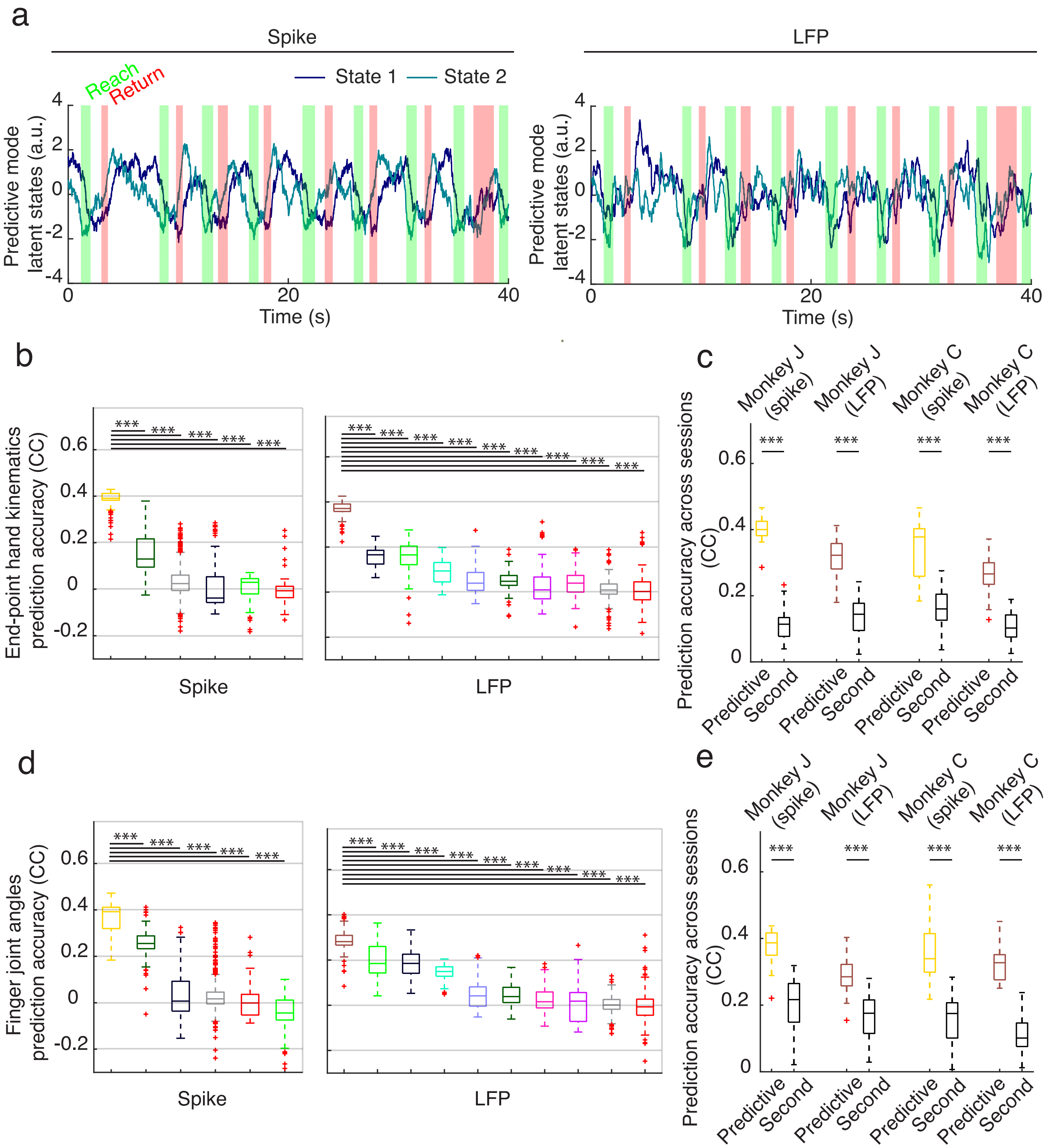

Supplementary Figure 3. Extended analyses of the predictive mode. **a** The predictive mode's two latent states are shown for both spiking network activity (left) and LFP network activity (right) in a sample experimental session. We extracted the reach and return periods from the temporal profiles of movement kinematics. This is done by extracting stationary periods when velocity and acceleration are

below certain thresholds and subsequently identifying movement initiation given increased velocity after stationary periods. Reach period (green) is defined as the period starting at when the subjects initiate the movement towards the target until they reach it. Similarly, return period (red) is defined as the period starting at when they initiate the backward movement towards the resting position until they reach it. **b** The predictive mode for arm joint angles was also the predictive mode for end-point hand kinematics (position and velocity in x, y and z dimensions) as well as finger joint angles ($P < 4.6 \times 10^{-22}$, $N_s >$ 97 and 99 cluster members for the yellow and brown clusters in **Fig. 3**, one-sided Wilcoxon rank sum test). Box plot of the prediction accuracy for the finger joint angles and 3D end-point hand kinematics for mode clusters in spiking and LFP network activity for the session shown in **Fig. 4a** and **Fig. 4b** are shown. Figure convention for box plot and significance is similar to **Figs. 3** and **4**. **c** In both monkeys and for both spiking and LFP network activity, the prediction accuracy of the end-point hand kinematics using the predictive mode is significantly better than that using the second best mode shown by black box plots ($P < 4.8 \times 10^{-5}$, $N_s =$ 35 and 20 cross-validation folds across sessions for Monkeys J and C, one-sided Wilcoxon signed-rank test). **d** Similar to (**b**) for finger joint angles ($P < 3.3 \times 10^{-13}$, $N_s >$ 97 and 99 cluster members for the yellow and brown clusters in **Fig. 3**, one-sided Wilcoxon rank sum test). **e** Similar to (**c**) for finger joint angles ($P < 4.8 \times 10^{-5}$, $N_s =$ 35 and 20 cross-validation folds across sessions for Monkeys J and C, one-sided Wilcoxon signed-rank test). Source data are provided as a Source Data file.

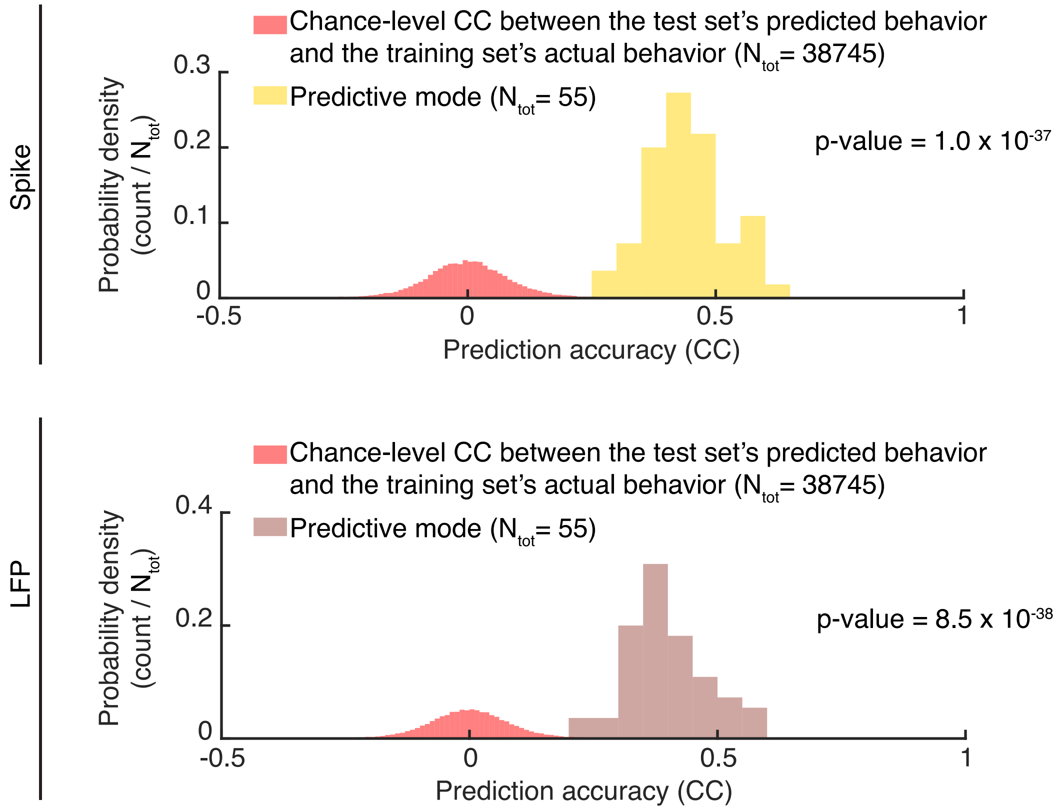

Supplementary Figure 4. The prediction accuracy of the predictive mode is significantly higher than the null distribution obtained by comparing the test set's predicted behavior to time-shifted segments of actual behavior of an equal length in the training set. Similar to a prior work[1], in the training set, we shift a window that has the same length as the test set every 250ms and take the equal-length behavior segments to be the behavior time-series in these windows. This process allows us to get a Monte Carlo estimate of the correlation coefficient between the predicted behavior in the test set and actual behavior in the training set across all sessions and monkeys. The distribution of these chance-level prediction accuracies in red are compared with the distribution of the predictive mode's prediction accuracies (yellow for spiking and brown for LFP). The predictive mode's prediction accuracy is significantly higher than this chance-level for both spiking network activity (top panel: $P = 1.0 \times 10^{-37}, N_s > 55$ cross-validation folds across sessions, one-sided Wilcoxon rank sum test) and LFP network activity (bottom panel: $P = 8.5 \times 10^{-38}, N_s > 55$ cross-validation folds across sessions, one-sided Wilcoxon rank sum test). Source data are provided as a Source Data file.

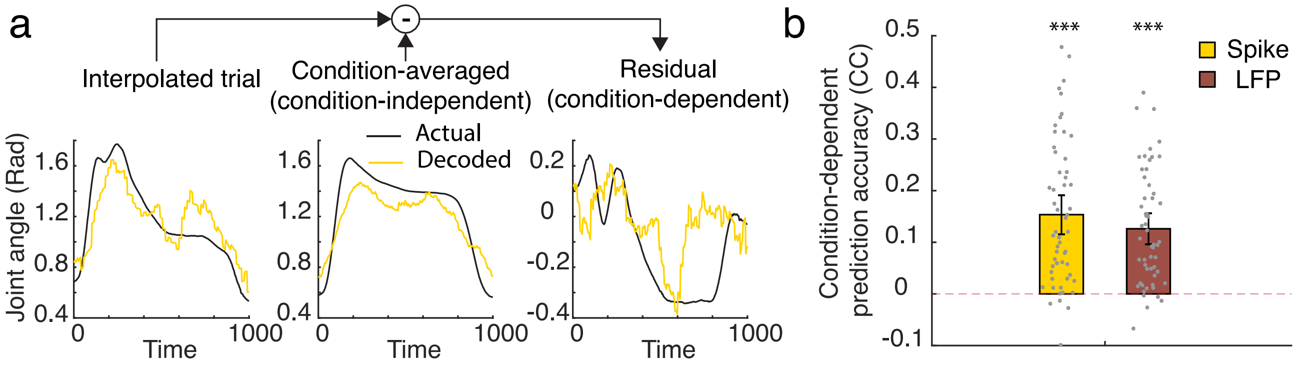

Supplementary Figure 5. Despite being very low-dimensional and being learned unsupervised with respect to movement, the predictive mode could also predict some condition-dependent residual movement in addition to the condition-averaged/independent movement. **a** Example movement trajectories of an interpolated trial, the condition-averaged (condition-independent) signal and residual (condition-dependent) signal are shown for shoulder elevation. We defined one trial as one full reach, grasp and return movement and extracted the trials as explained in **Supplementary Fig. 3a**. As the trials could have different lengths, we interpolated the signals for each trial to have a fixed length (*left*). We then computed the condition-averaged signals by averaging the actual movement across trials and by averaging the decoded movement with the predictive mode across trials (*middle*). By subtracting the condition-averaged signals from the interpolated signals, we get the residual signals for the actual and decoded movements in each trial (*right*). **b** The decoded movement's residual was correlated with the actual movement's residual: the Pearson's correlation coefficients (CC) between the two residual signals (in the same cross-validation analysis) were significantly positive (For spiking activity: $P = 8.3 \times 10^{-10}, N_s = 55$ cross-validation folds across sessions; For LFP activity: $P = 5.8 \times 10^{-10}, N_s = 55$ cross-validation folds across sessions; one-sided Wilcoxon singed-rank test). Figure convention for bars and asterisks is similar to **Fig. 3** and each grey dot represents residual prediction accuracy in one cross-validation fold. Source data are provided as a Source Data file.

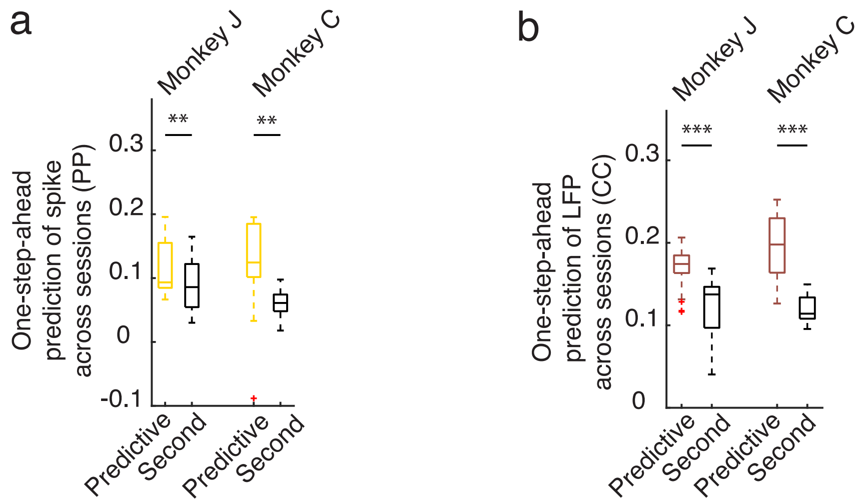

Supplementary Figure 6. One-step-ahead spiking and LFP prediction of mode clusters are shown across sessions. The predictive mode (of behavior) was also dominantly predictive of spiking and LFP network activity. Figure convention similar to **Figs. 3** and **4**. **a** Across monkeys, the predictive mode for behavior also had significantly better one-step-ahead prediction of spiking activity than the second best mode (second best in terms of one-step-ahead prediction of spiking activity; $P < 1.3 \times 10^{-3}$, $N_s = 35$ and 20 cross-validation folds across sessions for Monkeys J and C, one-sided Wilcoxon signed-rank test). **b** Similar to (**a**) for LFP activity ($P < 4.8 \times 10^{-5}$, $N_s = 35$ and 20 cross-validation folds across sessions for Monkeys J and C, one-sided Wilcoxon signed-rank test). Source data are provided as a Source Data file.

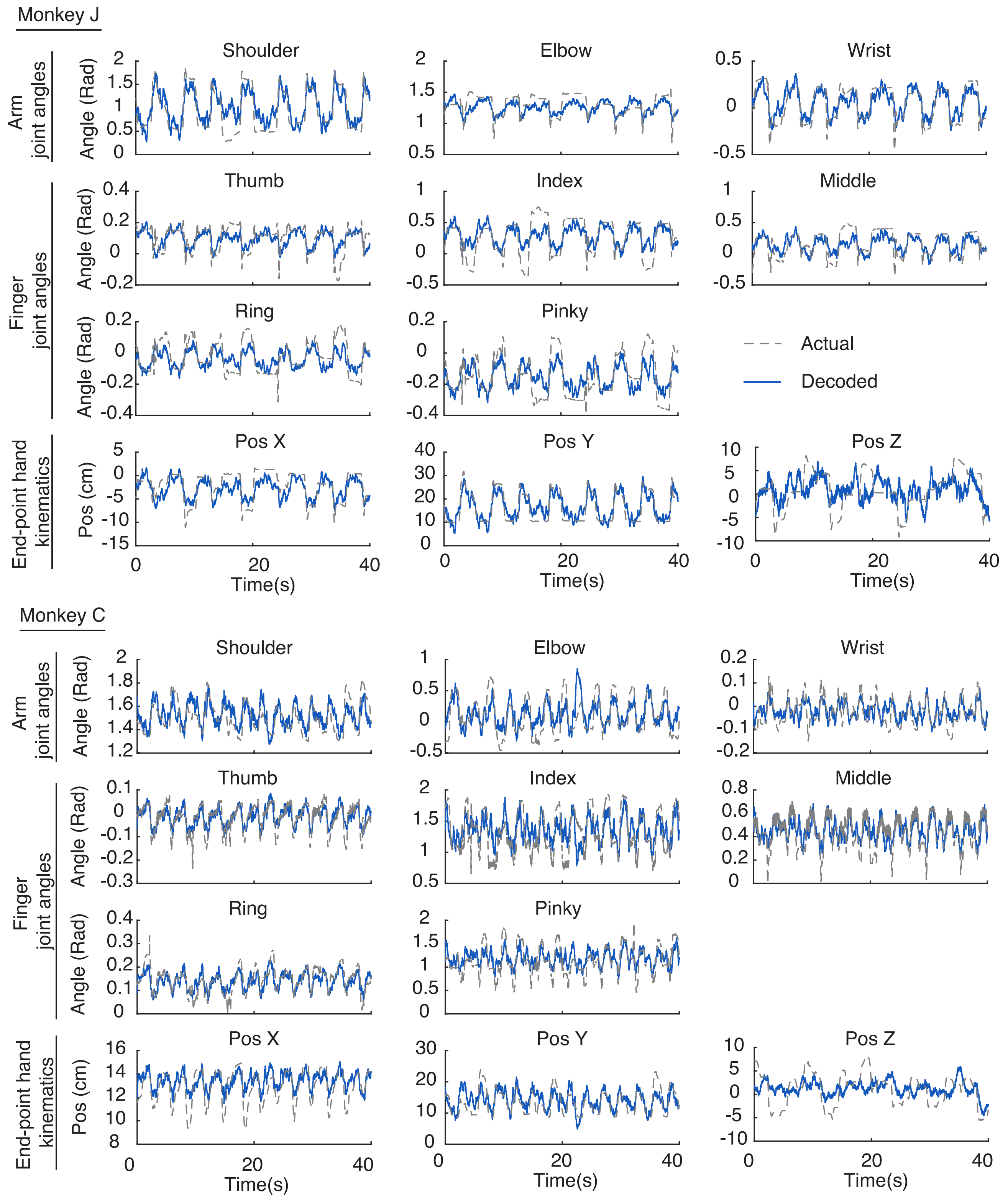

Supplementary Figure 7. Representative decoded trajectories with the multiscale predictive mode are shown for Monkey J (top) and Monkey C (bottom). We divided the joint angles into 8 groups: shoulder,

elbow, wrist, thumb, index, middle, ring, and pinky. In each group, we selected a representative joint angle such that it had the largest activity ratio among the joint angles in the group, where activity ratio is defined as the standard deviation of the variable divided by the peak-to-peak range. We show the actual and decoded trajectories for the representative set of joint angles and end-point hand kinematics. The representative joint angles for Monkey J were shoulder elevation, elbow flexion, wrist deviation, thumb proximal flexion, index proximal flexion, middle proximal flexion, ring proximal abduction and pinky proximal abduction. For monkey C, the representative joint angles were shoulder elevation, pro supination, wrist deviation, thumb middle flexion, index distal flexion, middle middle flexion, ring proximal abduction and pinky proximal abduction. Source data are provided as a Source Data file.

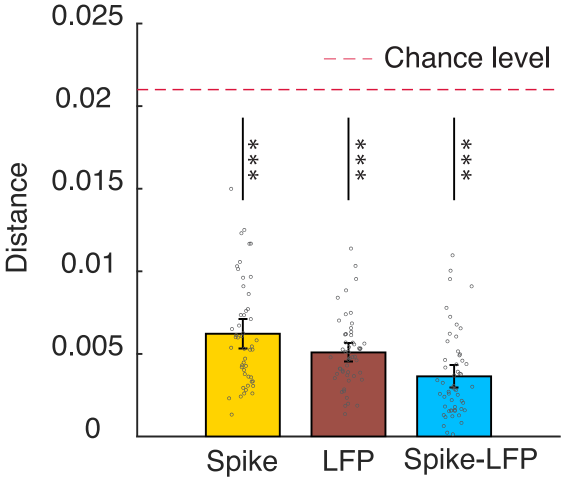

Supplementary Figure 8. The predictive mode, even when relearned from neural activity excluding the activity prior to movement initiation, remained largely consistent. Bars represent the distances between the relearned and original predictive modes in spiking, LFP and combined spike-LFP activity (**Fig. 5b**), which were significantly smaller than chance-level distance. Each grey dot represents the distance in one cross-validation fold in one session. Figure convention is similar to **Fig. 3**. In this analysis, we investigated the effect of signals to the motor cortex to initiate/plan the movements – for example signals due to presenting the movement goal or due to initiating the movement – by removing the neural activity prior to movement initiation, relearning the state-space model and re-identifying the predictive mode. To do this, we first extracted the reach and return periods from the temporal profiles of movement (similar to **Supplementary Fig. 3**). We then removed the neural activity in a 500 ms window prior to movement (reach) initiation. For LFP activity, we also removed the 300 ms window after movement initiation since LFP power features are calculated in causal 300 ms windows and this choice ensured that LFP power features did not contain any information from the 500 ms window prior to movement initiation. We relearned the state-space models from neural activity excluding these periods and tested whether the location of the predictive mode changed. We fixed the dimension of the latent state to 15, which is a dimension at which the eigenvalue-dimension diagrams had already converged to steady clusters. We found that the location of the predictive mode remained largely unchanged for spiking, LFP and combined spike-LFP activity: the distance of the relearned predictive mode to the original predictive modes shown in **Fig. 5b** was only 0.006 ± 0.003, 0.005 ± 0.002 and 0.004 ± 0.003 (mean $\pm$ std) for spiking, LFP and combined spike-LFP activity, respectively; these distances were significantly smaller than chance-level ($P < 3.9 \times 10^{-10}, N_s = 55$ cross-validation folds across sessions, one-sided Wilcoxon signed-rank test). Source data are provided as a Source Data file.

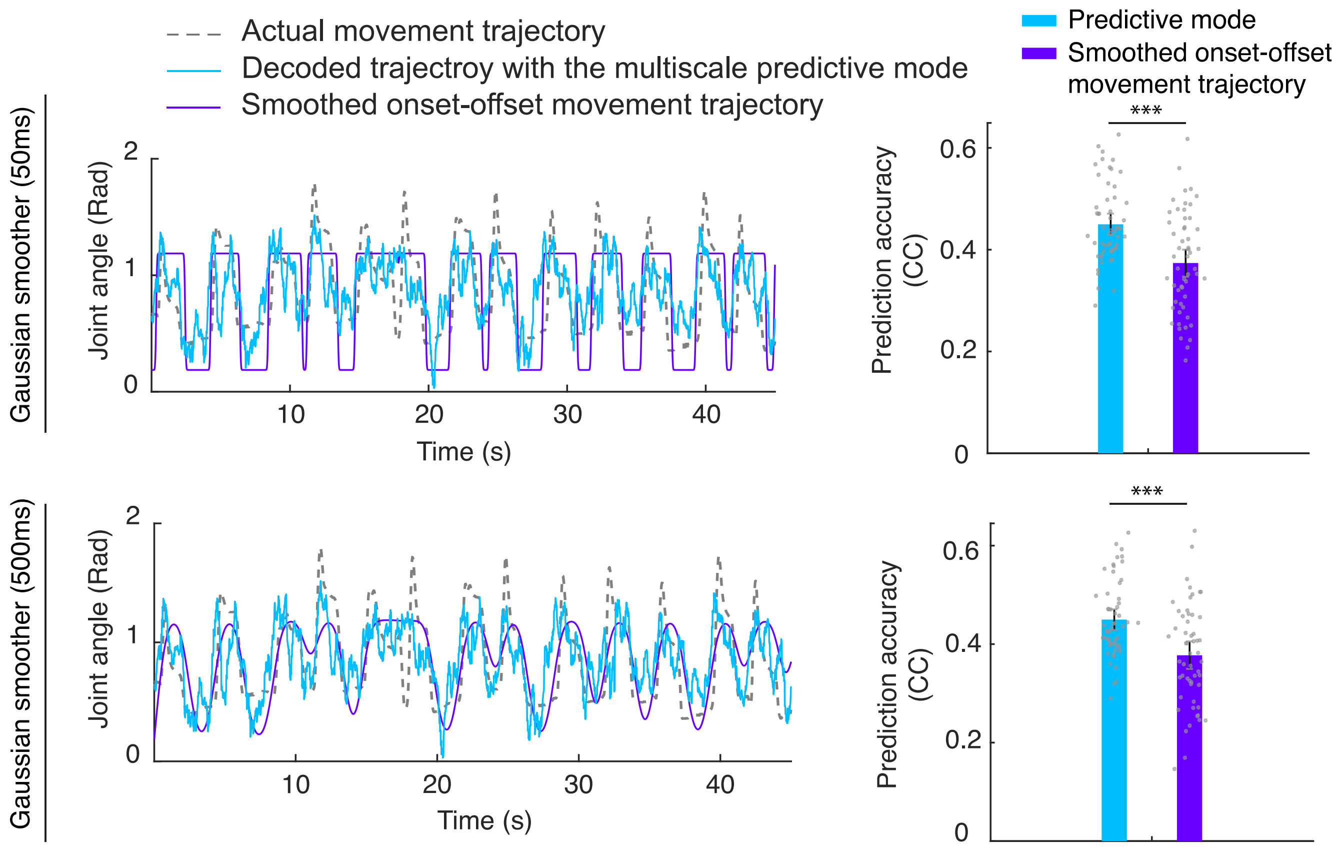

Supplementary Figure 9. The multiscale predictive mode has significantly higher prediction accuracy than the smoothed onset-offset movement trajectories. We first constructed an onset-offset movement trajectory that knows the exact times at which movement starts and ends. It turns "on" when subjects are moving and turns "off" when subjects are resting. To extract the smoothed onset-offset movement trajectories, we then applied a non-causal 50ms Gaussian kernel (top) and 500ms Gaussian kernel (bottom) to the onset-offset movement trajectories. For an example joint angle, shoulder elevation, we plot the actual movement, its decoded trajectory with the multiscale predictive mode in neural activity, and the smoothed onset-offset movement trajectory obtained from the movement itself – with perfect knowledge of onset-offset events – rather than from neural activity (left). Across all experimental sessions, the multiscale predictive mode had significantly higher prediction accuracies compared to the smoothed onset-offset movement trajectory (right; in both cases: $P < 1.3 \times 10^{-6}$, $N_s = 55$ cross-validation folds across sessions, one-sided Wilcoxon signed-rank test). Each grey dot represents the average joint angles prediction accuracy for one cross-validation fold in one experimental session. Note that this result held despite the fact that the onset-offset movement trajectories had perfect knowledge of the onset-offset events based on the actual movement. Figure conventions for bar plot and significance asterisks are similar to **Fig. 3**. Source data are provided as a Source Data file.

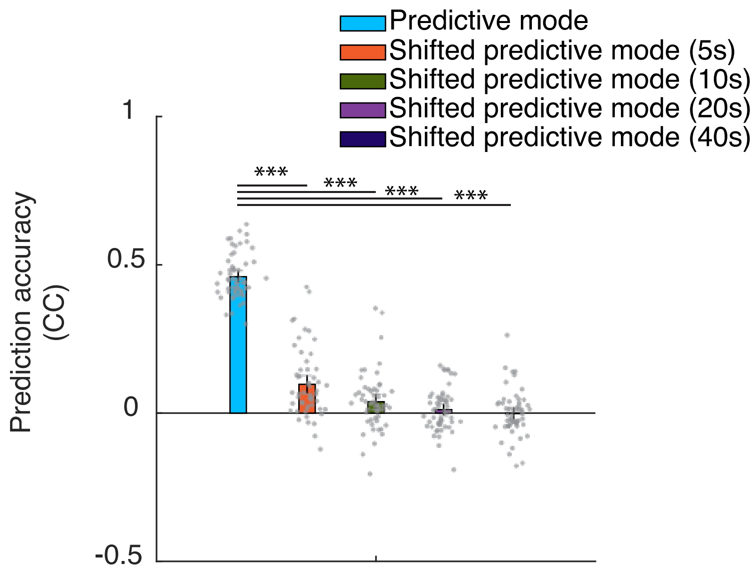

Supplementary Figure 10. The prediction accuracy of the multiscale predictive mode is not due to the autocorrelations in the neural activity and/or behavior. For this control analysis, we shifted the multiscale predictive mode's time-series in time by different amounts of 5s, 10s, 20s and 40s. We then relearned the linear regression matrix in the training set and used it to compute the predicted behavior in the test set with the shifted mode. This analysis maintains the autocorrelations in both neural activity and behavior regardless of the shift amount. The predictive mode's prediction accuracy is significantly higher than the prediction accuracy of the shifted predictive modes ($P < 5.7 \times 10^{-11}$, $N_s = 55$ cross-validation folds across sessions, one-sided Wilcoxon signed-rank test). Also, the shifted predictive mode's prediction accuracy approaches 0 as the shift approaches 40 seconds. Each grey dot represents the average joint angles prediction accuracy for one cross-validation fold in one experimental session. Figure conventions for bar plot and significance asterisks is similar to **Fig. 3**. Source data are provided as a Source Data file.

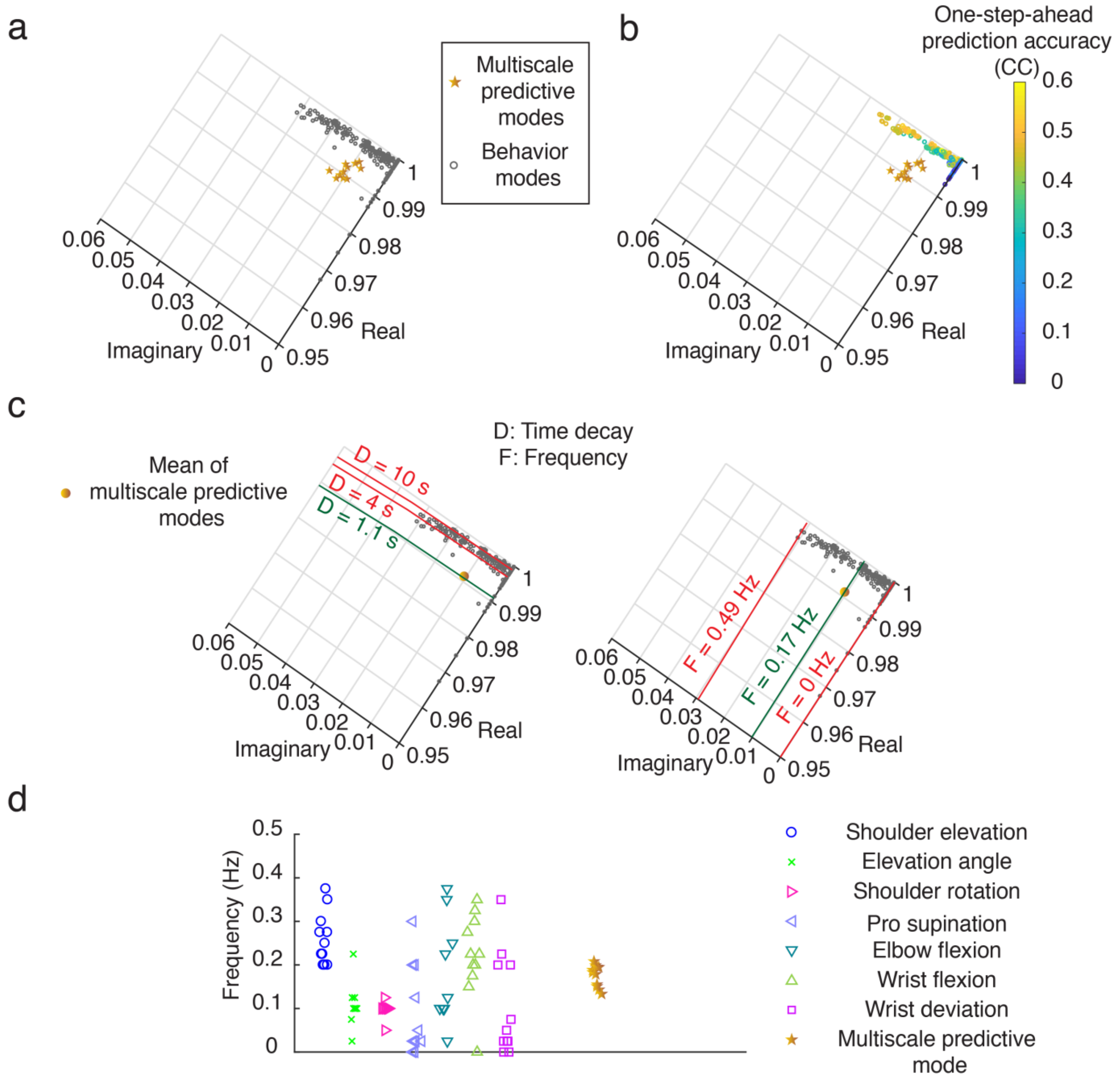

Supplementary Figure 11. The multiscale predictive mode was not simply due to direct representation or replication of behavior modes in neural activity. **a** The modes of behavior dynamics (pooled across all sessions) were different from the multiscale predictive mode in neural activity. To compute the modes in behavior dynamics, we extracted the eigenvalues of the matrix that directly describes how the joint angles $\mathbf{z}_t$ evolve in time, i.e. eigenvalues of $\mathbf{A}_{\text{kin}}$ in $\mathbf{z}_t = \mathbf{A}_{\text{kin}}\mathbf{z}_{t-1}$ (**Supplementary Note 2**). The yellow-brown stars represent the multiscale predictive modes (pooled across all sessions) computed as the mean of spiking and LFP activity mode centroids of each session in **Fig. 5b**. **b** In

addition to the direct matrix $\mathbf{A}_{\text{kin}}$ we also learned a latent state-space model for joint angles as we did for neural activity and looked at the eigenvalues of its state transition matrix – i.e. its modes (Equations (17) and (18) in **Supplementary Note 2**). These modes were very similar to direct behavior modes found in (**a**), yet again different from the multiscale predictive mode in neural activity. Behavior mode's color indicates the correlation coefficient (CC) between its one-step-ahead prediction of joint angles with their true values as shown in the color bar (shows their prevalence in behavior dynamics). We observed that the one-step-ahead prediction of the joint angles converged to a high accuracy at a latent state dimension of 7, suggesting that this dimension was sufficient for explaining the joint angle dynamics. Therefore, for simplicity, we only illustrate the modes of the model with a 7D latent state. **c** The decay (left) and frequency (right) for the behavior modes vs. the multiscale predictive mode in neural activity. The left and right figures are replicates and just highlight the decay or frequency values, respectively. The yellow-brown dot represents the mean location of the multiscale predictive modes across all sessions. The red and green lines show the contours on which either frequency or decay are constant. The decay of the behavior modes was between ~4-10 s while the decay of the multiscale predictive mode was 1.1 ± 0.49 s and significantly smaller than the behavior modes ($P = 3.5 \times 10^{-12}$, $N_s > 22$ spiking and LFP sessions, one-sided Wilcoxon rank sum test). In addition, the frequency of the behavior modes varied in a significantly larger range of 0–0.49 Hz compared with the multiscale predictive mode frequency, which was at 0.17 ± 0.03 Hz ($P = 7.1 \times 10^{-9}$, $N_s > 22$ spiking and LFP sessions, one-sided F-test for equal variances). **d** As yet another control, we computed the power spectral density (PSD) of joint angles using Welch's method with 50 s Hamming windows with 50% overlap. We then computed the peak frequency of joint angle PSDs across all experimental sessions as shown in the figure by the different colors. Consistent with our behavior modal analysis in (**a**-**c**), the PSD peak frequency of behavior across experimental sessions had a wider range compared with the multiscale predictive modes (yellow), which were located consistently at around 0.17Hz. Source data are provided as a Source Data file.

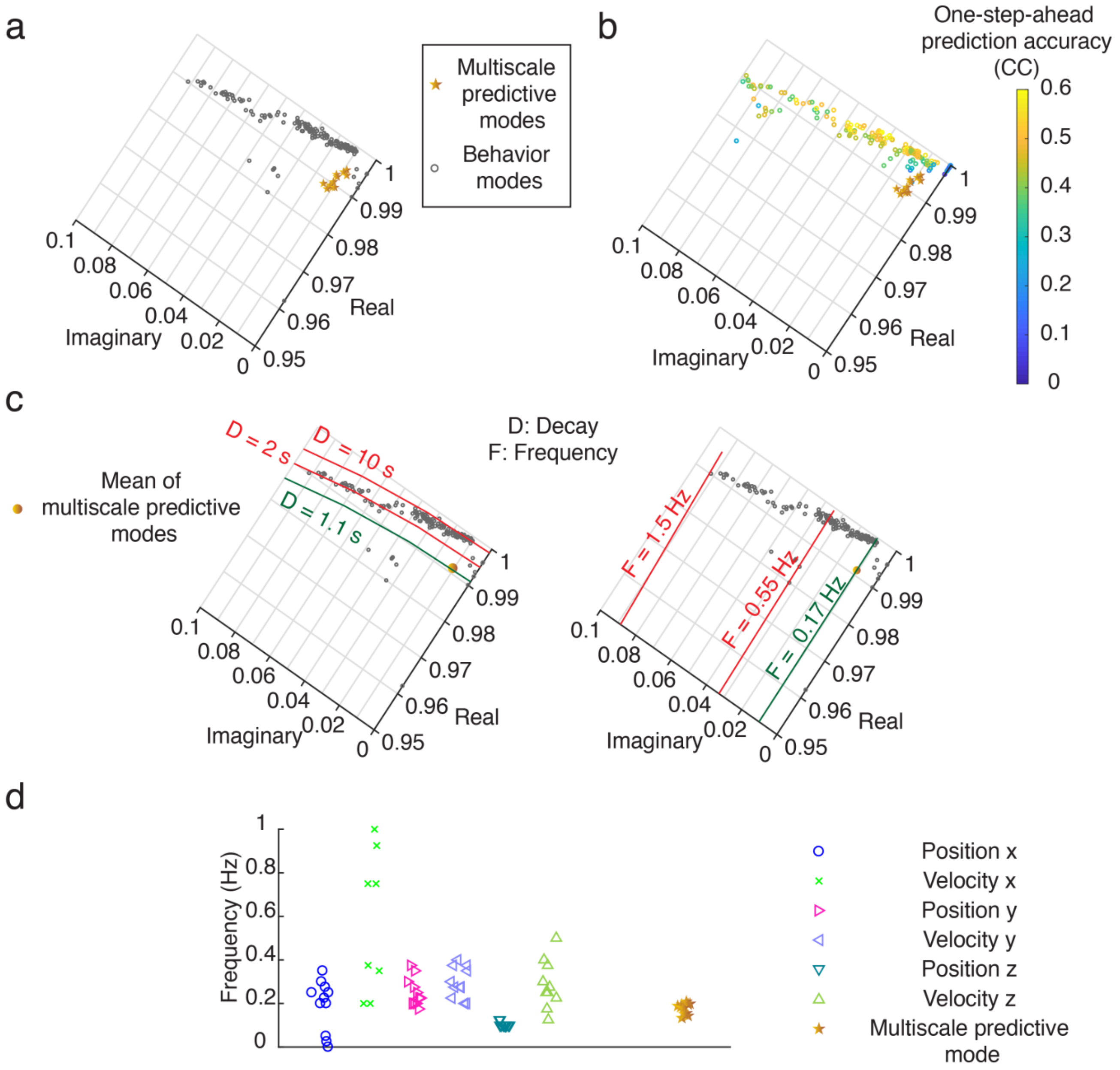

Supplementary Figure 12. Same as **Supplementary Fig. 11** but for 3D end-point hand kinematics (position and velocity in x, y and z directions). Note that the imaginary axis (or y axis) for the plots in (**a**-**d**) is different from the imaginary axis (or y axis) in **Supplementary Fig. 11**, as modes of hand kinematics had larger range of frequencies compared to joint angles. Similar to **Supplementary Fig. 11**, behavior modes in end-point hand kinematics had larger decays (in the range of 2–10s; $P = 1.5 \times 10^{-9}$, $N_s > 22$ spiking and LFP sessions, one-sided Wilcoxon rank sum test) and wider frequency ranges (in the range of 0.17–1.5 Hz; $P = 1.6 \times 10^{-17}$, $N_s > 22$ spiking and LFP sessions, one-sided F-

test for equal variances) compared to the multiscale predictive mode in neural activity. Source data are provided as a Source Data file.

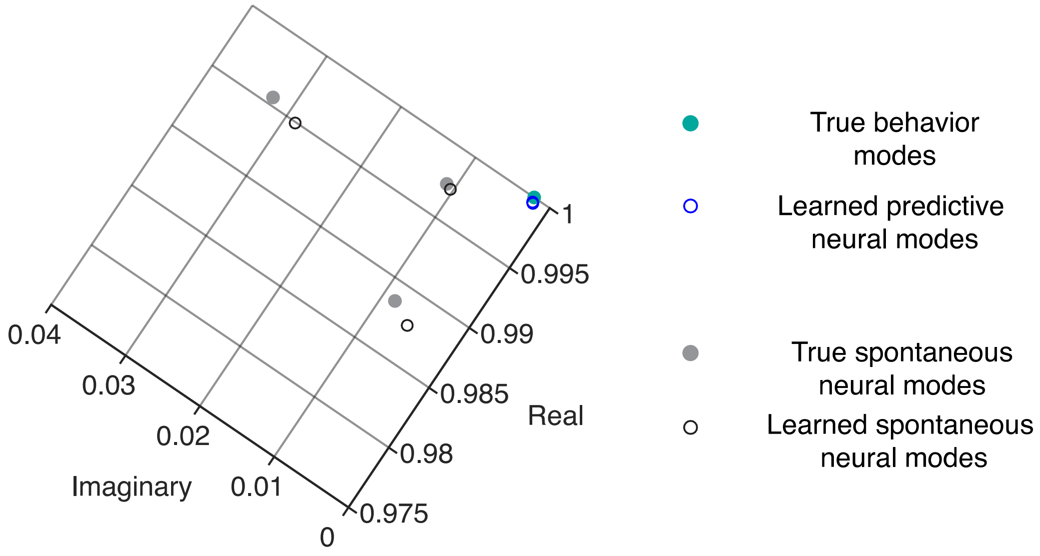

Supplementary Figure 13. A simulation for a hypothetical scenario where behavior is directly encoded in neural activity and additionally neural activity also exhibits other spontaneous behavior-irrelevant dynamics, i.e. non-predictive modes for behavior. We applied the exact same learning algorithm and modal analysis to this scenario for combined spike-field activity. In this case, the predictive modes in neural activity identified by our analyses were very close to the modes in behavior (**Supplementary Note 3**)**.** This was in contrast to the real data where we observed different modes in behavior dynamics (whether for joint angle or end-point hand kinematics) and in neural dynamics (**Supplementary Figs. 11** and **12**). In the simulation, complex conjugate modes which were learned and classified as the predictive modes in simulated neural activity had very similar dynamical characteristics as the true modes in simulated behavior. Other spontaneous non-predictive modes were learned accurately as well (normalized error $= 2.4\ \%$). This simulation shows that if neural activity was simply representing behavior, our analyses would have revealed the same modes in behavior and neural activity, unlike what we observe in our data. These results further confirm that the multiscale predictive mode is not simply due to direct representation of behavior modes in neural activity. Source data are provided as a Source Data file.

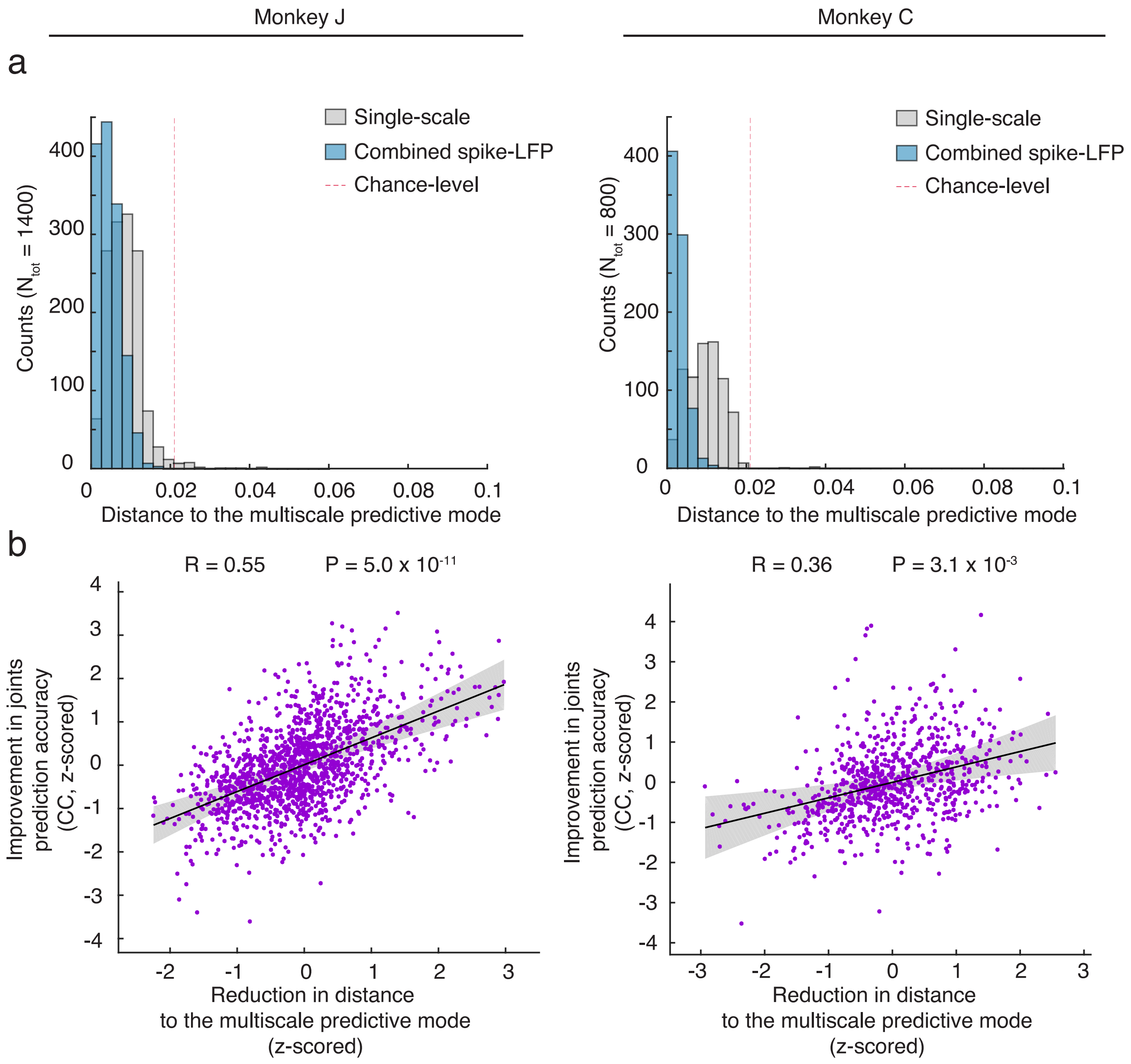

Supplementary Figure 14. Similar to **Fig. 6**, when the baseline number of single-scale channels (whether spike or LFP) in channel sets is fixed to be 10. **a** The estimated mode's distance to the multiscale predictive mode decreased significantly from single-scale to combined spike-LFP activity (Monkey J: $P = 8.2 \times 10^{-14}, N_s = 1400$ Monte Carlo samples; Monkey C : $P = 6.5 \times 10^{-24}, N_s = 800$ Monte Carlo samples, one-sided corrected resampled paired t-test, see **Methods**). **b** We found that there was a significant positive correlation between the reduction in distance and improvement in joint angle trajectory prediction accuracy ($P < 3.1 \times 10^{-3}$, $N_s = 1400$ and 800 Monte Carlo samples in Monkeys J and C, two-sided corrected resampled paired t-test). Similar to **Fig. 6**, black line is the linear least-square fit representing the mean prediction accuracy for different distances and the

shaded area is the 95% confidence bound of that mean. Source data are provided as a Source Data file.

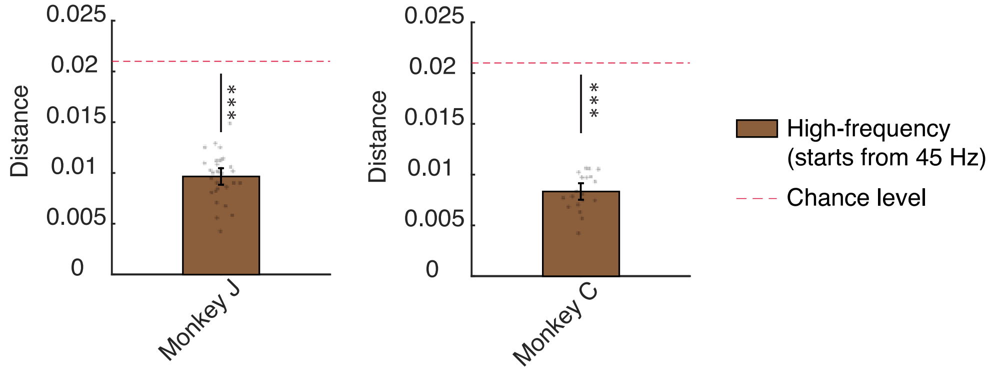

Supplementary Figure 15. The multiscale predictive mode was present in the high-frequency bands of LFP activity in the control analysis where high-frequency bands had 11 Hz spectral gap from low-frequency bands and started from 45 Hz whereas low-frequency bands ended at 34Hz. Figure convention is similar to **Fig. 7b** and shows that the mean distance of the closest estimated principal mode cluster to the multiscale predictive mode was significantly smaller than chance-level ($P < 4.8 \times 10^{-5}$, $N_s =$ 35 and 20 cross-validation folds across sessions for Monkeys J and C, one-sided Wilcoxon signed-rank test). Source data are provided as a Source Data file.

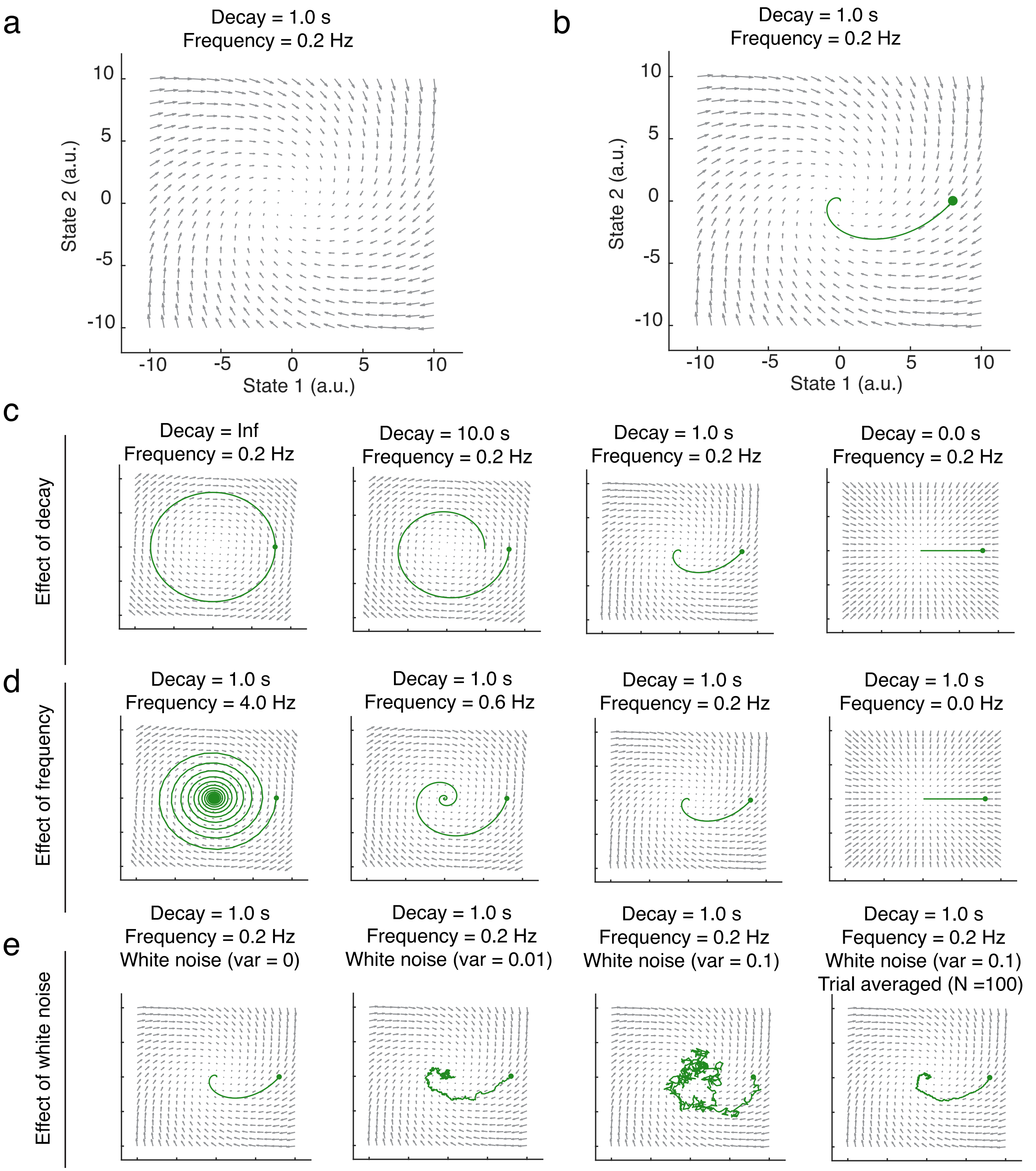

Supplementary Figure 16. Illustration of the effect of decay and frequency of the multiscale predictive mode. We construct a 2-dimensional state-space equation $\mathbf{x}_{t+1} = \mathbf{A}\mathbf{x}_t$ whose $\mathbf{A}$ is fully specified by the complex-conjugate eigenvalues corresponding to the multiscale predictive mode. We then plot the

vector field for this state-space model[2] and simulate a sample state trajectory for a fixed time interval (5 s) in response to an impulse excitation (initial condition). We also vary the decay and frequency of the mode by changing the eigenvalues of $\mathbf{A}$. **a** The vector field corresponding to the multiscale predictive mode exhibits rotations. State space is shown in arbitrary units (a.u.). **b** A sample state trajectory (shown by a green trajectory) from initial point (shown by a green dot) also shows rotations. **c** Mode decay determines how quickly the state decays to the fixed point of the dynamical system in response to an impulse excitation (initial condition). As we decrease the decay value from left to right, the state decays faster to the fixed point. **d** Mode frequency determines how fast the state trajectory rotates in response to an impulse excitation (initial condition). As we decrease the frequency from left to right, the state trajectory exhibits a smaller number of rotations for a fixed time interval in response to an impulse (initial condition). This can also be thought of as faster rotations for larger frequency values. **e** White noise in $\mathbf{x}_{t+1} = \mathbf{A}\mathbf{x}_t + \mathbf{w}_t$ only adds uncertainty to the single-trial state trajectory as shown by increasing the white noise variance term in $\mathbf{W}$ in the left three subplots in this panel (var = variance). Even when the noise is large, trial-averaged state trajectory still exhibits rotations (right subplot in this panel, N = number of trials). Source data are provided as a Source Data file.

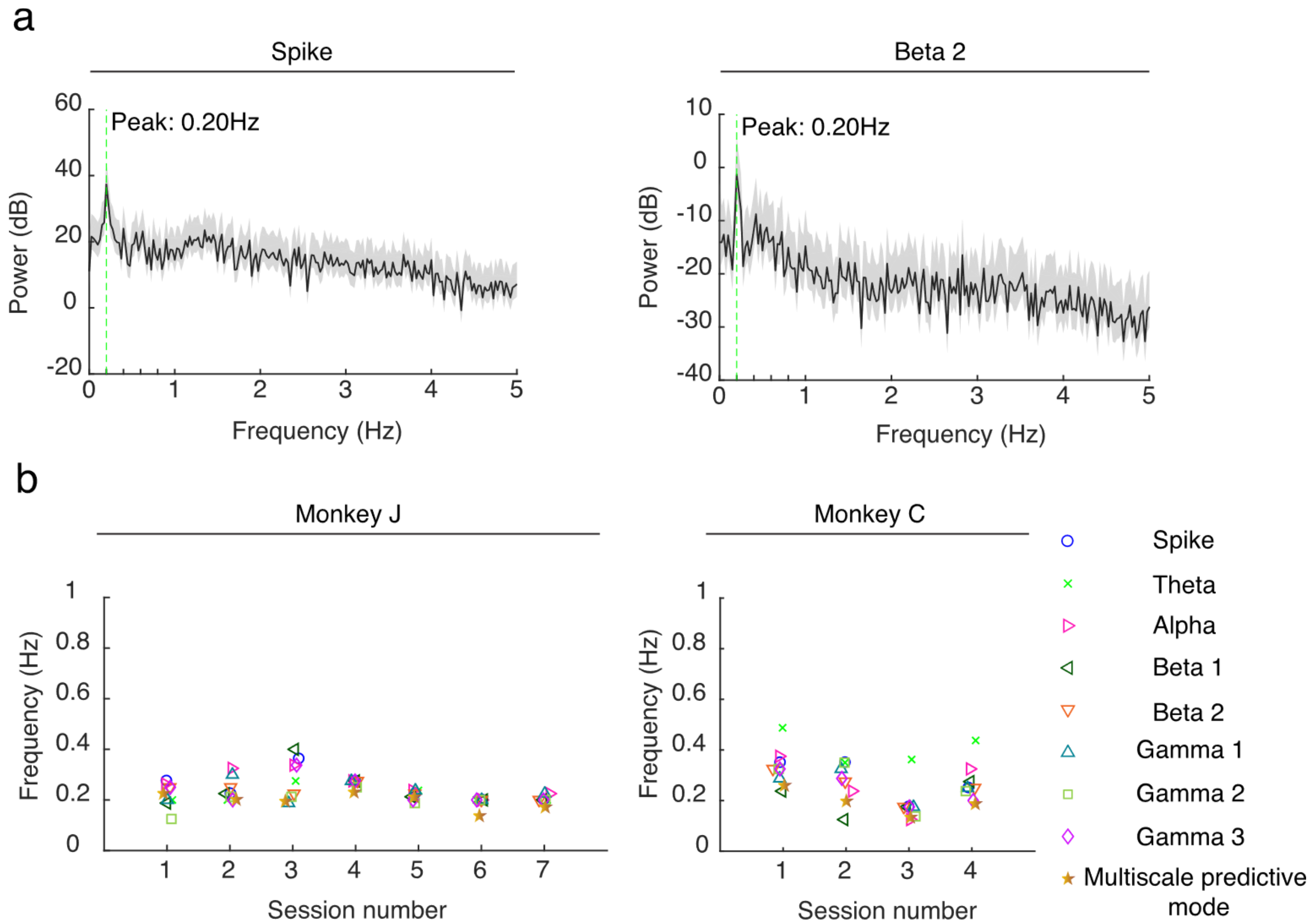

Supplementary Figure 17. Frequency characteristic of the multiscale predictive mode (frequency ~0.2 Hz) is close to the peak frequency in the power spectral density (PSD) of single-channel spiking and LFP activities. **a** PSD of spiking activity from a sample channel and PSD of the LFP beta 2 frequency band activity of another sample channel (both derived by Welch's method 50 s Hamming window with 50% overlap). There existed a dominant peak around frequency 0.2 Hz for both channels, again suggesting that the multiscale predictive mode frequency captures key dynamics of neural activity during naturalistic movements. We note that the frequency in the PSD is the frequency of the temporal variations in the power feature time-series and is unrelated to the frequency band of these features (we first compute the power feature in its corresponding band and then treat it as just a time-series from which we compute the PSD). For instance, when we say gamma 1 band for an LFP channel has a peak frequency in its PSD of around 0.2 Hz, it means the log-power feature computed from 35-55 Hz band of the LFP channel has *temporal variations* with peak frequency of 0.2 Hz. **b** Peak frequencies of PSD for spiking activity and 7 frequency bands of LFP activity were close to the multiscale predictive mode frequency. These frequencies were only 5.4 Hz apart on average across sessions. Each colored dot is the median of PSD peak frequency across channels for one session. Multiscale predictive mode frequency is computed as the mean frequency of the predictive mode centroids in spiking and LFP

activity in each session shown in **Fig. 5b**. The multiscale predictive mode frequency component was close to the PSD peak frequencies of the single-channel spiking and LFP features. Source data are provided as a Source Data file.

Supplementary Tables

Behavior variable	Monkey J				Monkey C			
	Min	Max	Mean	Std	Min	Max	Mean	Std
Engagement-to-rest ratio	1.33	2.28	1.66	0.31	1.32	4.74	2.88	1.46
Distance spanned by the 3D targets (cm)	18.14	25.76	22.26	3.08	14.21	27.39	18.87	5.82
Pos x (cm)	-14.16	8.47	-1.11	3.05	-2.97	29.69	11.07	3.21
Pos y (cm)	9.5	32.97	16.58	6.2	8.38	32.93	12.99	4.01
Pos z (cm)	-12.61	19.38	3.85	5.31	-11.59	15.84	0.57	4.56
Vel x (cm/s)	-62.95	53.53	0	9.62	-103.32	78.67	0	7.74
Vel y (cm/s)	-71.3	125.33	0	17.6	-62.35	92.95	0.01	12.14
Vel z (cm/s)	-97.77	95.31	0	11.35	-73.31	69.85	0	8.83
Shoulder elevation (Rad)	0.23	1.96	0.88	0.41	0.79	2.12	1.52	0.19
Elevation angle (Rad)	-0.71	1.82	1.13	0.31	0.8	1.83	1.5	0.11
Shoulder rotation (Rad)	-0.98	0.78	-0.07	0.27	-0.41	1.06	0.38	0.27
Elbow flexion (Rad)	0.46	2.22	1.44	0.2	0.27	2.4	0.89	0.3
Pro supination (Rad)	-0.49	1.71	1.14	0.19	-0.67	1.09	0.42	0.42
Wrist flexion (Rad)	-0.84	0.47	0.06	0.17	-0.77	0.91	-0.25	0.18
Wrist deviation (Rad)	-0.25	0.34	0.03	0.09	-0.24	0.35	-0.03	0.06
Thumb prox. flex. (Rad)	-0.98	0.32	-0.03	0.17	-0.54	0.67	-0.08	0.09
Thumb prox. abduct.	-0.6	0.65	-0.12	0.11	-0.56	0.74	0.1	0.12
Thumb middle flex. (Rad)	-0.35	1.63	0.08	0.27	-0.3	1.02	0.25	0.17
Thumb distal flex. (Rad)	-0.47	1.43	0.1	0.2	-0.41	1.48	0.21	0.22

Index prox. flex. (Rad)	-0.95	1.47	0.3	0.34	-0.95	1.01	0.37	0.32
Index prox. abduct. (Rad)	-0.42	0.72	0.08	0.15	-0.59	0.43	-0.03	0.14
Index middle flex. (Rad)	-0.31	1.64	0.18	0.24	-0.29	1.1	0.25	0.2
Index distal flex. (Rad)	0.08	2.03	1.02	0.24	0.31	2.1	1.16	0.34
Middle prox. flex. (Rad)	-0.64	1.21	0.2	0.26	-0.8	0.55	-0.01	0.21
Middle prox. abduct. (Rad)	-0.35	0.45	0.04	0.1	-0.46	0.43	-0.01	0.08
Middle middle flex. (Rad)	-0.28	1.59	0.43	0.19	-0.3	1	0.32	0.22
Middle distal flex. (Rad)	0.34	2.07	1.47	0.19				
Ring prox. flex. (Rad)	-0.81	1.29	0.22	0.26	-1.11	1.06	0.15	0.31
Ring prox. abduct. (Rad)	-0.42	0.49	0.04	0.12	-0.5	0.37	0	0.12
Ring middle flex. (Rad)	-0.29	1.67	0.05	0.19	-0.35	1.29	-0.28	0.06
Ring distal flex. (Rad)	0.14	1.92	1.07	0.22				
Pinky prox. flex. (Rad)	-1.03	1.18	-0.03	0.27	-0.79	1.09	-0.02	0.23
Pinky prox. abduct. (Rad)	-0.63	0.38	-0.09	0.14	-0.73	0.7	-0.15	0.14
Pinky middle flex. (Rad)	-0.32	1.81	0.37	0.29	-0.8	0.94	0.11	0.17
Pinky distal flex. (Rad)	-0.35	1.47	0.58	0.19	0.24	2.11	1.36	0.28

Supplementary Table 1. Behavior variable statistics for both subjects during experimental sessions. Engagement-to-rest ratio is defined as the total amount of time spent by the subjects to perform reach, grasp and return movements divided by the total amount of time they voluntarily spent at resting position. Distance spanned by the 3D targets (wand locations that the monkeys reached to) is defined as the 3D distance between the farthest targets in each experimental session. Two of joint angles (Middle distal flexion and Ring distal flexion) were missing in Monkey C due to technicalities. Joint angles names are shortened for compactness (prox.: proximal, flex.: flexion and abduct.: abduction). Source data are provided as a Source Data file.

Supplementary Note 1. Contribution of a given mode to behavior prediction and one-step-ahead prediction of spiking and LFP network activity

Here, we expand on the equations to compute the contribution of a given mode in the behavior prediction and one-step-ahead prediction of spiking and LFP activity. In the following, we consider the general case of a complex conjugate mode i, which has an associated complex conjugate eigenvalue pair denoted by e_{i_1} and e_{i_2}, located at the i_1^{th} and i_2^{th} diagonal entries of matrix $\mathbf{E}$ in equation $\mathbf{A} = \mathbf{U}\mathbf{E}\mathbf{U}^{-1}$. For a given real mode r_1 with associated eigenvalue e_{r_1}, which is located at the r_1^{th} diagonal entry of matrix $\mathbf{E}$, a similar procedure can be performed.

To later handle the bias of linear models, we define the operator $\cdot^{\{1\}}$ that returns the first column of a matrix and the operator $\cdot^{\{-1\}}$ which returns a matrix obtained by removing the first column of the original matrix. These two operators can be applied to any matrix (or any row vector) with more than one column.

Behavior prediction

We can rewrite equation (6) with the expansion in (9) as:

$$\hat{\mathbf{z}}_t = \hat{\mathbf{L}}\tilde{\mathbf{x}}_{t|t} = \hat{\mathbf{L}}^{\{1\}} + \hat{\mathbf{L}}^{\{-1\}}\mathbf{x}_{t|t} = \hat{\mathbf{L}}^{\{1\}} + \sum_{j=1}^{n_x}\left[\mathrm{col}_j\left(\hat{\mathbf{L}}^{\{-1\}}\mathbf{U}\right)\mathrm{row}_j\left(\mathbf{x}_{t|t}^{\mathrm{modal}}\right)\right]. \quad (10)$$

Given the above equation, we can compute the contribution of mode i in behavior prediction, denoted by $\hat{\mathbf{z}}_{i,t}$, by writing down the terms in the above sum that correspond to the ith mode as:

$$\hat{\mathbf{z}}_{i,t} = \hat{\mathbf{L}}^{\{1\}} + \mathrm{col}_{i_1}\left(\hat{\mathbf{L}}^{\{-1\}}\mathbf{U}\right)\mathrm{row}_{i_1}\left(\mathbf{x}_{t|t}^{\mathrm{modal}}\right) + \mathrm{col}_{i_2}\left(\hat{\mathbf{L}}^{\{-1\}}\mathbf{U}\right)\mathrm{row}_{i_2}\left(\mathbf{x}_{t|t}^{\mathrm{modal}}\right). \quad (11)$$

The term $\hat{\mathbf{L}}^{\{1\}}$ is used for every mode to handle the bias in the equation.

One-step-ahead prediction of spiking activity

The one-step-ahead predicted spiking probability with the expansion in (9) can be written as:

$$\lambda_{\mathrm{c}}\left(\tilde{\mathbf{x}}_{t|t-1}\right) = \exp\left(\boldsymbol{\alpha}_c \tilde{\mathbf{x}}_{t|t-1}\right)$$
$$= \exp\left(\boldsymbol{\alpha}_{\mathrm{c}}^{\{1\}} + \boldsymbol{\alpha}_{\mathrm{c}}^{\{-1\}} \mathbf{x}_{t|t-1}\right) = \exp\left(\boldsymbol{\alpha}_{\mathrm{c}}^{\{1\}} + \sum_{j=1}^{n_x} \left[\mathrm{col}_j\left(\boldsymbol{\alpha}_c^{\{-1\}} \boldsymbol{U}\right) \mathrm{row}_j\left(\mathbf{x}_{t|t-1}^{\mathrm{modal}}\right)\right]\right), \tag{12}$$

$$\left\{\widehat{N}_{\mathrm{prob}}^{c}\right\} = \left\{\lambda_{\mathrm{c}}\left(\tilde{\mathbf{x}}_{t|t-1}\right)\Delta \mid t \leq T\right\}$$
$$= \left\{\exp\left(\boldsymbol{\alpha}_{\mathrm{c}}^{\{1\}} + \sum_{j=1}^{n_x} \left[\mathrm{col}_j\left(\boldsymbol{\alpha}_c^{\{-1\}} \mathbf{U}\right) \mathrm{row}_j\left(\mathbf{x}_{t|t-1}^{\mathrm{modal}}\right)\right]\right)\Delta \mid t \leq T\right\}.$$

Given the above equation, we can compute the contribution of mode i in one-step-ahead prediction of spiking probability, denoted by $\widehat{N}_{i,\mathrm{prob}}^{c}$, by writing down the terms in the above sum that correspond to the ith mode as:

$$\left\{\widehat{N}_{i,\mathrm{prob}}^{c}\right\}$$
$$= \left\{\exp\left(\boldsymbol{\alpha}_c^{\{1\}} + \mathrm{col}_{i_1}\left(\boldsymbol{\alpha}_c^{\{-1\}} \mathbf{U}\right) \mathrm{row}_{i_1}\left(\mathbf{x}_{t|t-1}^{\mathrm{modal}}\right) + \mathrm{col}_{i_2}\left(\boldsymbol{\alpha}_c^{\{-1\}} \mathbf{U}\right) \mathrm{row}_{i_2}\left(\mathbf{x}_{t|t-1}^{\mathrm{modal}}\right)\right)\Delta \middle| t \leq T\right\}. \tag{13}$$

The term $\boldsymbol{\alpha}_c^{\{1\}}$ is used for every mode to handle the bias in the equation. Then we can define the contribution of mode i to one-step-ahead prediction of spiking activity as the AUC ROC corresponding to this spiking probability contribution in equation (13).

One-step-ahead prediction of LFP activity

We can rewrite the one-step-ahead prediction of LFP activity with the expansion in (9) as:

$$\hat{\mathbf{y}}_{t|t-1} = \mathbf{C}\tilde{\mathbf{x}}_{t|t-1} = \mathbf{C}^{\{1\}} + \mathbf{C}^{\{-1\}}\mathbf{x}_{t|t-1} = \mathbf{C}^{\{1\}} + \sum_{j=1}^{n_x}\left[\mathrm{col}_j\left(\mathbf{C}^{\{-1\}}\mathbf{U}\right)\mathrm{row}_j\left(\mathbf{x}_{t|t-1}^{\mathrm{modal}}\right)\right],$$

$$\{\hat{Y}\} = \{\hat{\mathbf{y}}_{t|t-1} \mid t \in \mathbb{T}_{\mathrm{f}}\} = \{\mathbf{C}^{\{1\}} + \sum_{j=1}^{n_x}\left[\mathrm{col}_j\left(\mathbf{C}^{\{-1\}}\mathbf{U}\right)\mathrm{row}_j\left(\mathbf{x}_{t|t-1}^{\mathrm{modal}}\right)\right] \mid t \in \mathbb{T}_{\mathrm{f}}\}. \quad (14)$$

Given the above equation, we can compute the contribution of mode i in one-step-ahead prediction of LFP activity, denoted by $\{\hat{Y}_i\}$, by writing down the terms in the above sum that correspond to the ith mode:

$$\{\hat{Y}_i\} = \{\mathbf{C}^{\{1\}} + \mathrm{col}_{i_1}\left(\mathbf{C}^{\{-1\}}\mathbf{U}\right)\mathrm{row}_{i_1}\left(\mathbf{x}_{t|t-1}^{\mathrm{modal}}\right) + \mathrm{col}_{i_2}\left(\mathbf{C}^{\{-1\}}\mathbf{U}\right)\mathrm{row}_{i_2}\left(\mathbf{x}_{t|t-1}^{\mathrm{modal}}\right) \mid t \in \mathbb{T}_{\mathrm{f}}\} \quad (15)$$

The term $\mathbf{C}^{\{1\}}$ is used for every mode to handle the bias in the equation.

Supplementary Note 2. Modal analysis of behavior dynamics

To extract the modes of dynamics in behavior $\mathbf{z}_t$, we employed two different approaches. First, we looked at the eigenvalues of matrix $\mathbf{A}_{\text{kin}}$ learned directly from the behavior trajectory data in the following equation:

$$\mathbf{z}_t = \mathbf{A}_{\text{kin}}\mathbf{z}_{t-1}. \tag{16}$$

We simply get $\mathbf{A}_{\text{kin}}$ using linear least-squares estimation (LLSE). Eigenvalues of $\mathbf{A}_{\text{kin}}$ represent the modes of dynamics in behavior trajectories.

As the second approach, we extract the modes of behavior dynamics using exactly the same procedure used to extract the mode of neural dynamics. In particular, we extract the modes of behavior dynamics by building a dynamical model where the behavior (joint angles or end-point hand kinematics) are the observations of the latent state-space formulation (similar to neural activity being the observations). We form the state equation as:

$$\mathbf{o}_{t+1} = \mathbf{A}_{\text{b}}\mathbf{o}_t + \mathbf{q}_t, \tag{17}$$

where $\mathbf{o}_t$ is the column vector of latent states, $\mathbf{A}_{\text{b}}$ is the state transition matrix and $\mathbf{q}_t$ is a zero-mean white Gaussian noise with covariance matrix $\mathbf{Q}_{\text{b}}$. The observation equation is a linear model given by

$$\mathbf{z}_t = \mathbf{C}_{\text{b}}\mathbf{o}_t + \mathbf{m}_t, \tag{18}$$

where $\mathbf{z}_t$ is the column vector of behavior (either joint angles or end-point hand kinematics), $\mathbf{C}_{\text{b}}$ is the observation matrix and $\mathbf{m}_t$ is a zero-mean white Gaussian noise with covariance matrix $\mathbf{M}_{\text{b}}$. We find the

parameters ($\{\mathbf{A}_{\mathrm{b}}, \mathbf{Q}_{\mathrm{b}}, \mathbf{C}_{\mathrm{b}}, \mathbf{M}_{\mathrm{b}}\}$) of this dynamical model using the EM algorithm for linear Gaussian observations and perform a similar modal analysis as explained for neural activity. We also compute the contribution of modes to prediction of behavior to understand the importance of each mode in behavior dynamics, again exactly similarly to what we did for neural activity.

Supplementary Note 3. Simulation of a dynamical latent state-space model with direct representation of behavior in neural activity

To see what our algorithm outputs in a hypothetical scenario where neural activity directly represents behavior, we performed a simulation analysis. For the sake of simplicity, we simulated a task in which the behavior is the 4D hand kinematics (position and velocity) in x-y coordinates. We simulated 200 trials of the task where subjects performed reaches to random targets located on a 10×10 grid similar to the set-up explained in our prior theoretical work[3]. In our simulation, combined spike-LFP activity encodes the latent state. To form a latent state that contains a direct representation of behavior, we constructed the latent state to consist of the hand kinematics (i.e., contains behavior modes) in addition to some other dimensions corresponding to spontaneous modes that are unrelated to behavior. In particular, we fixed the latent state dimension to 10, of which 4 dimensions were assigned to behavior (position and velocity in x and y coordinates) and the other 6 dimensions formed 3 spontaneous complex conjugate modes. We simulated 30 spike channels and 150 LFP features using equations (1) – (4) during a 2D movement generated with an optimal feedback-control model— details of the simulation can be found elsewhere[3].

We followed the same procedure as we did in our data analyses to get the true modes in behavior dynamics from the simulated hand kinematics $\mathbf{z}_t$; in particular, we used linear least squares estimation (LLSE) on simulated hand kinematics to get the $\mathbf{A}_{\text{kin}}$ in equation: $\mathbf{z}_t = \mathbf{A}_{\text{Kin}}\mathbf{z}_{t-1}$ (similar to approach 1 in **Supplementary Note 2**). We also computed the true spontaneous neural modes by finding the eigenvalues of the true state transition matrix ($\mathbf{A}$) in (1), which we had simulated data from.

We then used the simulated neural activity to learn its modes. We used the multiscale EM algorithm to learn the dynamical model from simulated spike-LFP network activity and compute its modes. We then compared these learned modes from neural activity to (i) the true modes in behavior and (ii) the true

spontaneous neural modes in neural activity. To do this, we classified the learned modes from neural activity using K-means clustering based on their prediction accuracy of behavior into two groups: (i) Learned predictive neural modes, which we compared to true modes in behavior; (ii) Learned spontaneous neural modes, which we compared to true spontaneous neural modes. In our prior theoretical work, we validated this approach of finding the two mode groups through extensive numerical simulations[3].

Supplementary References

1. Vargas-Irwin, C. E. *et al.* Decoding complete reach and grasp actions from local primary motor cortex populations. *J. Neurosci.* **30**, 9659–9669 (2010).
2. Perko, L. *Differential Equations and Dynamical Systems*. **7**, (Springer New York, 2001).
3. Abbaspourazad, H., Hsieh, H.-L. & Shanechi, M. M. A Multiscale Dynamical Modeling and Identification Framework for Spike-Field Activity. *IEEE Trans. Neural Syst. Rehabil. Eng.* **27**, 1–1 (2019).
