# Supplementary material for: Multiscale low-dimensional motor cortical state dynamics predict naturalistic reach-and-grasp behavior
Source: Nat Commun. 2021 Jan 27;12:607. doi: 10.1038/s41467-020-20197-x (PMC7840738; doi:10.1038/s41467-020-20197-x)
Supplement: Supplementary file 2 — Reporting Summary [file 41467_2020_20197_MOESM2_ESM.pdf]

nature research

Corresponding author(s): Maryam M. Shanechi

Last updated by author(s): 11/3/2020

Reporting Summary

Nature Research wishes to improve the reproducibility of the work that we publish. This form provides structure for consistency and transparency in reporting. For further information on Nature Research policies, see our Editorial Policies and the Editorial Policy Checklist.

Statistics

For all statistical analyses, confirm that the following items are present in the figure legend, table legend, main text, or Methods section.

n/a	Confirmed	
☐	☒	The exact sample size (n) for each experimental group/condition, given as a discrete number and unit of measurement
☐	☒	A statement on whether measurements were taken from distinct samples or whether the same sample was measured repeatedly
☐	☒	The statistical test(s) used AND whether they are one- or two-sided<br>*Only common tests should be described solely by name; describe more complex techniques in the Methods section.*
☐	☒	A description of all covariates tested
☐	☒	A description of any assumptions or corrections, such as tests of normality and adjustment for multiple comparisons
☐	☒	A full description of the statistical parameters including central tendency (e.g. means) or other basic estimates (e.g. regression coefficient) AND variation (e.g. standard deviation) or associated estimates of uncertainty (e.g. confidence intervals)
☐	☒	For null hypothesis testing, the test statistic (e.g. F, t, r) with confidence intervals, effect sizes, degrees of freedom and P value noted<br>*Give P values as exact values whenever suitable.*
☐	☒	For Bayesian analysis, information on the choice of priors and Markov chain Monte Carlo settings
☐	☒	For hierarchical and complex designs, identification of the appropriate level for tests and full reporting of outcomes
☐	☒	Estimates of effect sizes (e.g. Cohen's d, Pearson's r), indicating how they were calculated

Our web collection on statistics for biologists contains articles on many of the points above.

Software and code

Policy information about availability of computer code

Data collection	Kinematic data were acquired using the Cortex software package (version 5.3) to track retroreflective markers in 3D (Motion Analysis, Inc USA). Joint angles were solved from the 3D marker data using a Rhesus macaque musculoskeletal model via the SIMM toolkit (version 4.0, MusculoGraphics Inc., USA).
Data analysis	Custom code (MATLAB version R2017a) for the multiscale EM algorithm is available online at https://github.com/ShanechiLab/multiscaleEM

For manuscripts utilizing custom algorithms or software that are central to the research but not yet described in published literature, software must be made available to editors and reviewers. We strongly encourage code deposition in a community repository (e.g. GitHub). See the Nature Research guidelines for submitting code & software for further information.

Data

Policy information about availability of data

All manuscripts must include a data availability statement. This statement should provide the following information, where applicable:

- Accession codes, unique identifiers, or web links for publicly available datasets
- A list of figures that have associated raw data
- A description of any restrictions on data availability

The data used to support the findings of this study are available within the article, its Supplementary Information files, the Source Data file or upon reasonable request from the corresponding author. Source data are provided with this paper.

Field-specific reporting

Please select the one below that is the best fit for your research. If you are not sure, read the appropriate sections before making your selection.

☒ Life sciences ☐ Behavioural & social sciences ☐ Ecological, evolutionary & environmental sciences

For a reference copy of the document with all sections, see nature.com/documents/nr-reporting-summary-flat.pdf

Life sciences study design

All studies must disclose on these points even when the disclosure is negative.

Sample size	A sample size of two non-human primate (NHP) subjects was used. Demonstration in two NHPs is standard for NHP electrophysiology studies and similar to those reported in previous publications (a list of such publications is provided in Online Methods). All results held for all subjects.
Data exclusions	No data was excluded from the study.
Replication	Results were replicated in two subjects for each experimental task and all attempts at replication were successful.
Randomization	Not relevant for this study. Identical analyses were performed on data from each subject and the results were reported for each subject. There was no grouping of subjects.
Blinding	Not relevant for this study. There was no group allocation.

Reporting for specific materials, systems and methods

We require information from authors about some types of materials, experimental systems and methods used in many studies. Here, indicate whether each material, system or method listed is relevant to your study. If you are not sure if a list item applies to your research, read the appropriate section before selecting a response.

Materials & experimental systems

n/a	Involved in the study	
☒	☐	Antibodies
☒	☐	Eukaryotic cell lines
☒	☐	Palaeontology and archaeology
☐	☒	Animals and other organisms
☒	☐	Human research participants
☒	☐	Clinical data
☒	☐	Dual use research of concern

Methods

n/a	Involved in the study	
☒	☐	ChIP-seq
☒	☐	Flow cytometry
☒	☐	MRI-based neuroimaging

Animals and other organisms

Policy information about studies involving animals; ARRIVE guidelines recommended for reporting animal research

Laboratory animals	Two adult male rhesus macaques (macaca mulatta): Monkey J (5 years old) and Monkey C (8 years old).
Wild animals	This study did not involve wild animals.
Field-collected samples	This study did not involve field-collected samples.
Ethics oversight	All surgical and experimental procedures were performed in compliance with the National Institute of Health Guide for Care and Use of Laboratory Animals and were approved by the New York University Institutional Animal Care and Use Committee.

Note that full information on the approval of the study protocol must also be provided in the manuscript.
